# The global, regional, national burden of nasopharyngeal cancer and its attributable risk factors (1990–2019) and predictions to 2035

**DOI:** 10.1002/cam4.4783

**Published:** 2022-04-27

**Authors:** Yexun Song, Wenwei Cheng, Heqing Li, Xiajing Liu

**Affiliations:** ^1^ Department of Otolaryngology‐Head Neck Surgery The Third Xiangya Hospital of Central South University Changsha China; ^2^ The Third Xiangya Hospital of Central South University Changsha China; ^3^ Xiangya School of Public Health Central South University Changsha China; ^4^ Graduate School of Guilin Medical University Guilin China

**Keywords:** epidemiology, global burden of disease, nasopharyngeal cancer, prediction, risk factors

## Abstract

We aim to report the latest incidence, mortality, and disability‐adjusted life‐years (DALYs) between 1990 and 2019, by age, sex, sociodemographic index (SDI), and provide predictions to 2035. We use estimates from Global Burden of Disease, Injuries, and Risk Factors Study 2019 to analyze the incidence, mortality, and DALYs. All the estimates were shown as counts and age‐standardized rates (ASR). In 2019, there were more than 176,501 (156,046 to 199,917) incidence cases, with ASRs of 2.1 (1.9 to 2.4). Nasopharyngeal cancer (NPC) accounted for 71,610 (65,442 to 77,625) deaths, with ASRs of 0.9 (0.8 to 0.9). NPC was also responsible for 2.34 million (2,139,753 to 2,536,657) DALYs, with ASRs of 28.0 (25.7 to 30.4). The count of all the new cases increased from 1990 to 2019. At the regional level, the highest age‐standardized incidence rates were found in East Asia, the highest age‐standardized death and DALY rates were shown in Southeast Asia. At the national level, the age‐standardized incidence rates were highest in Singapore, and the age‐standardized death and DALY rates were highest in Malaysia. The total numbers and rates of all the estimates were significantly higher among males than females across most of the age groups. The considerable burden of NPC was attributable to alcohol use, smoking, and occupational exposure to formaldehyde. A total of six GBD regions and 88 countries are projected to experience an increase in NPC ASRs between 2019 and 2035, respectively. Despite the current decline in age‐standardized mortality and DALY rates globally, the age‐standardized incidence rate has increased from 1990 to 2019, and continues to increase between 2020 and 2035, indicating that nasopharyngeal cancer remains a major health challenge worldwide. Prevention strategies should focus on modifiable risk factors, especially among males in East Asia.

## Graphical abstract

The development of nasopharyngeal cancer will cause immensely medical, health and economic burdens that must be considered. We investigated the latest incidence, death, and DALYs due to nasopharyngeal cancer and its risk factors from 1990 to 2019 and provide predictions to 2035. Despite the current and predicted decline in age‐standardized incidence globally, the absolute number of estimates continue to increase. Prevention programs should concentrate on modifiable risk factors, especially among the males across all age groups.

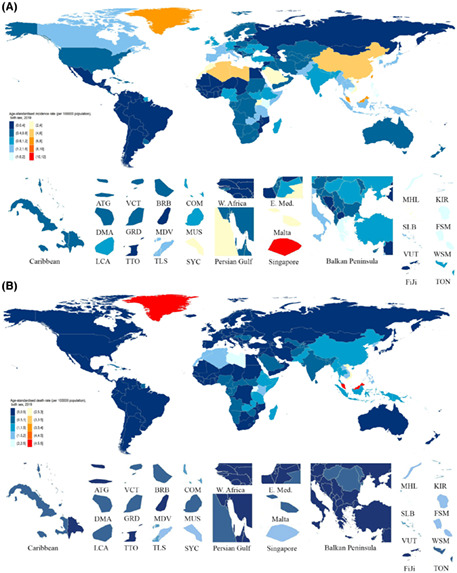

## 1 | INTRODUCTION

Nasopharyngeal cancer (NPC) is a type of head and neck malignancy arising from the nasopharyngeal epithelium, which is characterized by a distinct geographical distribution in East and Southeast Asia.^1^, ^2^ According to the data of International Agency for Research on Cancer, there were approximately 129,000 new cases with an ASR of 3.0 for NPC, accounting for 0.7% of all the cancers diagnosed in 2018.^3^ Thus, the NPC will cause immense medical, health and economic burdens that must be considered, especially for developing countries in Southeast Asia.

The etiology and pathogenesis of NPC remain obscure, and the significantly geographical distribution of NPC has stimulated research on potential risk factors. Many factors, including Epstein–Barr virus (EBV) infection, alcohol, smoking, and environmental factors, contributed to the development of NPC.^4^, ^5^ All the potential risk factors for NPC need to be further verified by a large amount of clinical data at global levels.

The prognosis of NPC is not particularly satisfactory because it is mainly detected at the late stage of the disease due to its concealed location. Until now, although there was significant improvement in the early diagnosis and treatment strategy for NPC, the social and economic burden is still increasing. Further knowledge about the epidemiological characteristics of NPC is needed to reallocate limited health resources, which is useful for the prevention, diagnosis, and therapy of NPC.

The Global Burden of Diseases, Injuries, and Risk Factors Study (GBD) 2019 is the most comprehensive dataset assessing the burden of diseases, injuries, and risk factors; the estimates were finalized and became accessible in 2020.^6^ Our study is the first to investigate the global burden from 1990 to 2019 and predict future trends from 2020 to 2035 in nasopharyngeal cancer, aiming to illustrate temporal changes in the past and future.^7^, ^8^, ^9^

## 2 MATERIALS AND METHODS

### 2.1 Study data

The data in our study were from the GBD database by query tool (http://ghdx.healthdata.org/gbd‐results‐tool). The methodology of the GBD 2019 supported by the IHME and its main improvements compared with previous cycles have been introduced in previous publications.^7^, ^8^, ^9^, ^10^ Detailed information about the estimation and prediction process were described in the Data S1.

### 2.2 Statistical analysis

We used counts and ASR per 100,000 population with 95% uncertainty intervals (UIs) to evaluate the burden of NPC. Incidence, prevalence, deaths, YLDs, YLLs, and DALYs were metrics used to quantify the burden of NPC and reported by age, sex, year, location, and SDI. We calculated the annual percentage change of ASRs to describe the ASR trends. The GBD 2019 used the comparative risk‐assessment framework to quantify associations between diseases and 87 risk factors.^11^, ^12^

All statistics were based on the R program (version 4.0.3) or SPSS 25.0 (IBM Corporation, New York, USA). *P* values less than 0.05 were considered statistically significant.

## 3 RESULTS

### 3.1 Global level

**FIGURE 1:**
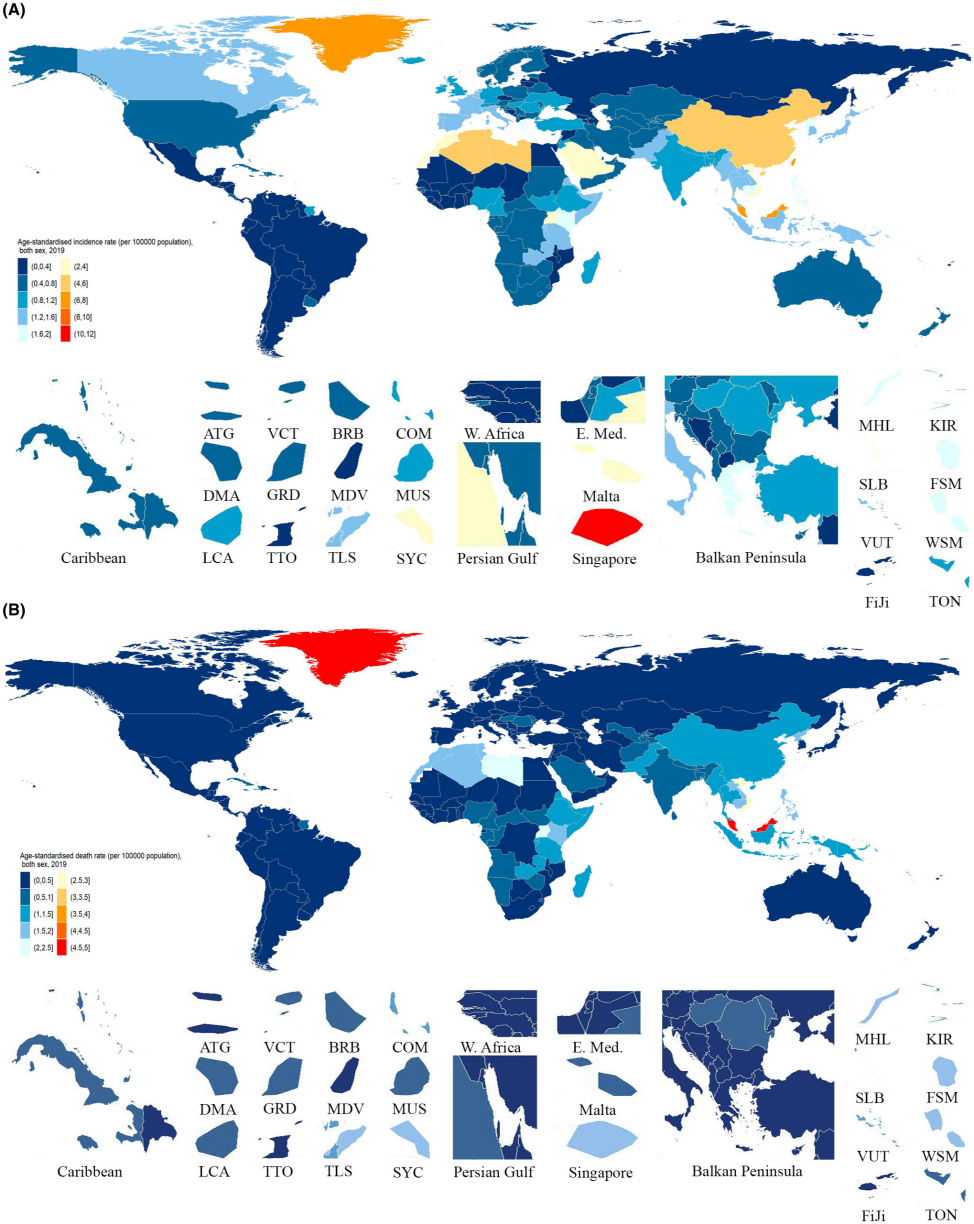
Age‐standardized incidence (A) and death (B) rates of NPC per 100,000 population both sex, 2019. ATG, Antigua and Barbuda; BRB, Barbados; COM, Comoros; DMA, Dominica; FSM, Federated States of Micronesia; GRD, Grenada; KIR, Kiribati; LCA, Saint Lucia; MDV, Maldives; MHL, Marshall Islands; MUS, Mauritius; SLB, Solomon Islands; VCT, Saint Vincent and the Grenadines; SYC, Seychelles; TLS, Timor‐Leste; TON, Tonga; TTO, Trinidad and Tobago; VUT, Vanuatu; WSM, Samoa

Nasopharyngeal cancer was the underlying cause of an estimated 176,501 (95% UI 156046 to 199,917) new cases and 71,610 (65,442 to 77,625) deaths globally in 2019 (Figure S2 and S3). The age‐standardized incidence rate was 2.1 (1.9 to 2.4), and the age‐standardized mortality rate was 0.9 (0.8 to 0.9) (Figure 1). A total of 2.34 million (2,139,753 to 2,536,657) DALYs with an age‐standardized rate of 28.0 (25.7 to 30.4) were observed in NPC (Table S1).

Between 1990 and 2019, the global age‐standardized incidence rate increased by 37.1% (18.2% to 59.4%), the mortality rate decreased by −31.3% (−38.9% to −22.5%), and the DALY rate decreased by −32.9% (−40.6% to −23.8%) (Table S1). From 1990 to 2019, the total number of new cases increased by 161.4% (124.4% to 204.5%) from 67,518 (61,729 to 72,995) to 176,501 (156,046 to 199,917), the mortality counts increased by 34.0% (19.3% to 51.8%) from 53,459 (48,875 to 57,906) to 71,610 (65,442 to 77,625), and total DALYs increased by 24.2% (9.7% to 41.1%) from 1.88 million (1,715,976 to 2,050,611) to 2.34 million (2,139,753 to 2,536,657) (Tables S5).

### 3.2 Regional level

The largest incidence numbers of NPC in 2019 were in East Asia (113,395, 95% UI 93188 to 135,377), South Asia (15,778, 13,772 to 18,199), and Southeast Asia (13,120, 11,396 to 15,082). The lowest incidence numbers were in Andean Latin America (102, 81 to 125), Oceania (109, 79 to 143), and Southern Sub‐Saharan Africa (228, 177 to 291). The largest numbers of NPC deaths in 2019 were in East Asia (30,103, 25,229 to 35,361), South Asia (14,665, 12,957 to 16,861), and Southeast Asia (11,259, 9866 to 12,847). The lowest death numbers were in Andean Latin America (89, 72 to 109), Oceania (103, 75 to 134), and Australasia (133, 119 to 148). East Asia (958,331, 810,443 to 1,124,172), South Asia (508,458, 449,583 to 583,996), and Southeast Asia (364,166, 316,978 to 418,042) had the largest number of DALYs due to NPC, whereas these numbers were lowest in Andean Latin America (2547, 2027 to 3164), Oceania (3566, 2561 to 4741), and Australasia (3733, 3358 to 4127) (Table S1). In all regions, the incidence, mortality, and DALY numbers were higher among males than females in 2019, except for the incidence in North Africa and the Middle East, Australasia (Figure S7).

Among the 21 regions included in the GBD 2019, East Asia (5.6, 4.7 to 6.7), Southeast Asia (2.0, 1.7 to 2.2), and high‐income Asia Pacific (1.7, 1.5 to 2.0) had the highest NPC age‐standardized incidence rates, whereas these rates were lowest in Andean Latin America (0.2, 0.1 to 0.2), Central Latin America (0.2, 0.2 to 0.3), and the Tropical Latin America (0.3, 0.3 to 0.3). Southeast Asia (1.7, 1.5 to 2.0), East Asia (1.4, 1.2 to 1.7), and Oceania (1.3, 0.9 to 1.6) had the highest age‐standardized death rates, whereas these rates were lowest in Andean Latin America (0.2, 0.1 to 0.2), Southern Latin America (0.2, 0.2 to 0.2), and the Central Latin America (0.2, 0.2 to 0.2). Southeast Asia (52.3, 45.7 to 60.0), East Asia (46.3, 39.3 to 54.0), and Eastern Sub‐Saharan Africa (38.4, 28.9 to 47.0) had the highest DALY rates in 2019, whereas Andean Latin America (4.3, 3.4 to 5.3), Central Latin America (5.3, 4.5 to 6.4), and Southern Latin America (5.5, 5.1 to 6.0) had the lowest DALY rates. The age‐standardized incidence, death, and DALY rates in 2019 were higher for males in all GBD regions, except for the incidence in Australasia, North Africa, and the Middle East (Figure S6).

The percentage change in age‐standardized incidence, mortality, and DALY rates varied significantly in the 21 GBD regions among males and females from 1990 to 2019. Consistent with the percentage change trends, the incidence rate increased in most of the regions, but the death and DALY rates declined in most of the regions (Figure S14).

### 3.3 National level

China had the highest incidence counts (110,433, 90,342 to 132,397), followed by India (12,212, 10,384 to 14,415) and Japan (3985, 3238 to 4847) (Figure S2 and Table S7). China also had the highest number of death cases (28,659, 23,780 to 34,066), followed by India (11,358, 9717 to 13,434) and Indonesia (3217, 2507 to 4134) (Figure S3 and Table S7). Further, China had the highest number of DALYs (912,107, 760,829 to 1,081,932), followed by India (388,094, 331,090 to 459,149) and Indonesia (100,797, 78,200 to 131,184) (Table S7).

The highest incidence rates were found in Singapore (10.8, 95% UI 8.3 to 14.2), Taiwan (Province of China) (7.1, 5.3 to 9.7), and Malaysia (6.1, 4.6 to 7.8) in 2019. The lowest incidence rates were found in Niger (0.1, 0.1 to 0.2), Mozambique (0.1, 0.1 to 0.2), and Mali (0.1, 0.1 to 0.2). In 2019, the age‐standardized mortality rates were highest in Malaysia (4.8, 3.6 to 6.1), Greenland (4.7, 3.8 to 5.7), and Brunei Darussalam (2.9, 2.5 to 3.4). In contrast, the lowest age‐standardized mortality rates were found in Finland (0.1, 0.1 to 0.1), Sweden (0.1, 0.1 to 0.1), and Norway (0.1, 0.1 to 0.1) (Figure 1). The highest estimated age‐standardized DALY rates in 2019 were observed in Malaysia (152.3, 115.7 to 195.1), Greenland (137.7, 109.3 to 171.5), and Brunei Darussalam (89.8, 76.4 to 105.6). Conversely, Finland (3.2, 2.8 to 3.8), Sweden (3.3, 3.0 to 3.6), and Chile (3.3, 3.0 to 3.8) had the lowest age‐standardized DALY rates in 2019 (Table S7).

From 1990 to 2019, the percentage change in age‐standardized incidence rates differed substantially among countries, with Cabo Verde (216.4%, 143.6% to 307.1%), Romania (196.0%, 132.3% to 272.0%), and Cyprus (159.2%, 101.1% to 234.0%) showing the largest increases. In contrast, Kuwait (−45.3%, −57.1% to −29.4%), Greenland (−45.3%, −57.5% to −29.7%), and Latvia (−40.8%, −55.7% to −22.8%) showed the largest decreases in age‐standardized incidence rates. The percentage change in age‐standardized mortality rates also differed among countries. The largest increases were seen in Cabo Verde (185.8%, 121.4% to 263.1%), Romania (123.9%, 78.7% to 175.0%), and Jamaica (77.0%, 33.9% to 128.6%). But, the largest declines during this period were found in Singapore (−68.3%, −72.3% to −63.1%), France (−63.8%, −68.8% to −58.3%), and Estonia (−57.6%, −69.8% to −42.4%). Similarly, the percentage change in age‐standardized DALY rates from 1990 to 2019 also differed among countries. The largest increases were seen in Cabo Verde (196.2%, 123.8% to 284.0%), Romania (118.1%, 70.9% to 168.3%), and Jamaica (87.1%, 40.3% to 143.9%). In contrast, the largest declines were observed in Singapore (−69.2%, −73.5% to −63.8%), France (−63.6%, −69.0% to −57.4%), and Kuwait (−59.8%, −68.9% to −48.0%) (Table S7).

### 3.4 Age and sex patterns

**FIGURE 2:**
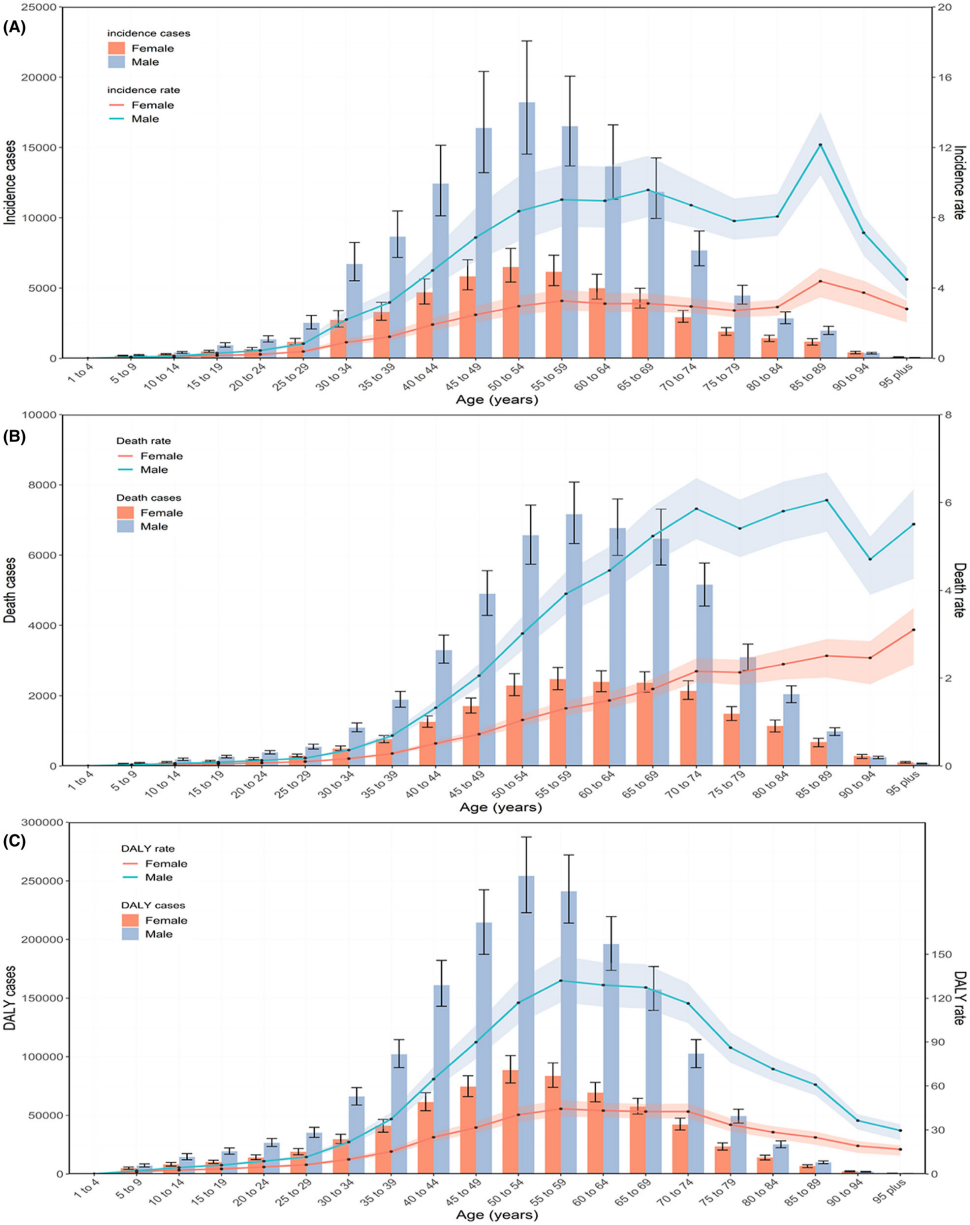
Global counts and age‐standardized incidence (A), death (B) and DALY (C) rates of NPC by age and sex, 2019. Error bars indicate the 95% uncertainty intervals (95% UI) for incidence (A), death (B) and DALYs (C). Shading indicates the upper and lower limits of the 95% UI

In 2019, most of the global numbers and rates of incidence, death, and DALY were higher in males than females, except for incidence, death, and DALY cases in the 90–94 years and 95 plus years groups, showing a significant gender gap that started from 25–29 years to 85–89 years (Figure 2).

The global number of incidence cases, death cases, and DALYs followed a normal distribution; this pattern was similar between males and females, whereas it peaked at 55–59 years for death cases and 50–54 years for incidence cases and DALYs. The trends in the global age‐standardized incidence, death, and DALY rates varied significantly in a nonlinear manner with increasing age between males and females (Figure 2).

### 3.5 Burden of NPC by sociodemographic index

**FIGURE 3:**
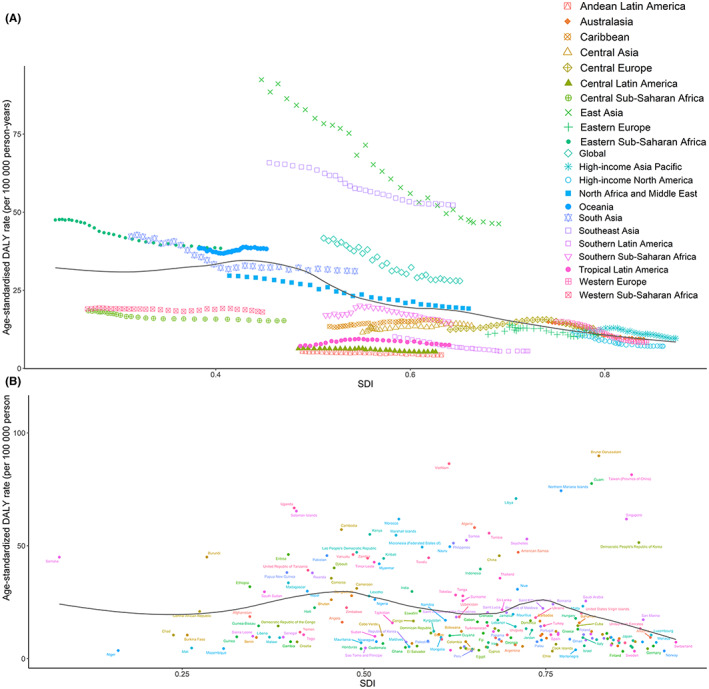
Age‐standardized DALY rates for NPC for 21 Global Burden of Disease regions (A) from 1990 to 2019 and for 204 countries and territories (B) in 2019, both by SDI. The black line represents the expected age‐standardized DALY rates based solely on SDI

Figure 3A presents the trends in the DALY rate across SDI by region between 1990 and 2019. The expected pattern was nonlinear, peaking at an SDI value of approximately 0.45. Most of the regions showed a decreasing trend in age‐standardized DALY rates in the study period, including East Asia, which had the largest decreases. Central Asia, the Caribbean, and Central Europe showed increases in observed age‐standardized DALY rates, with increasing SDI values.

At the national level, the expected pattern was nonlinear, with two peaks at SDI values of approximately 0.45 and 0.75. The age‐standardized DALY rate in countries, including Malaysia, Greenland, and Vietnam, was significantly higher than the expected levels, whereas others, such as Germany, Egypt, and Niger, were lower than the expected levels based on the SDI (Figure 3B).

### 3.6 Attributable risks

**FIGURE 4:**
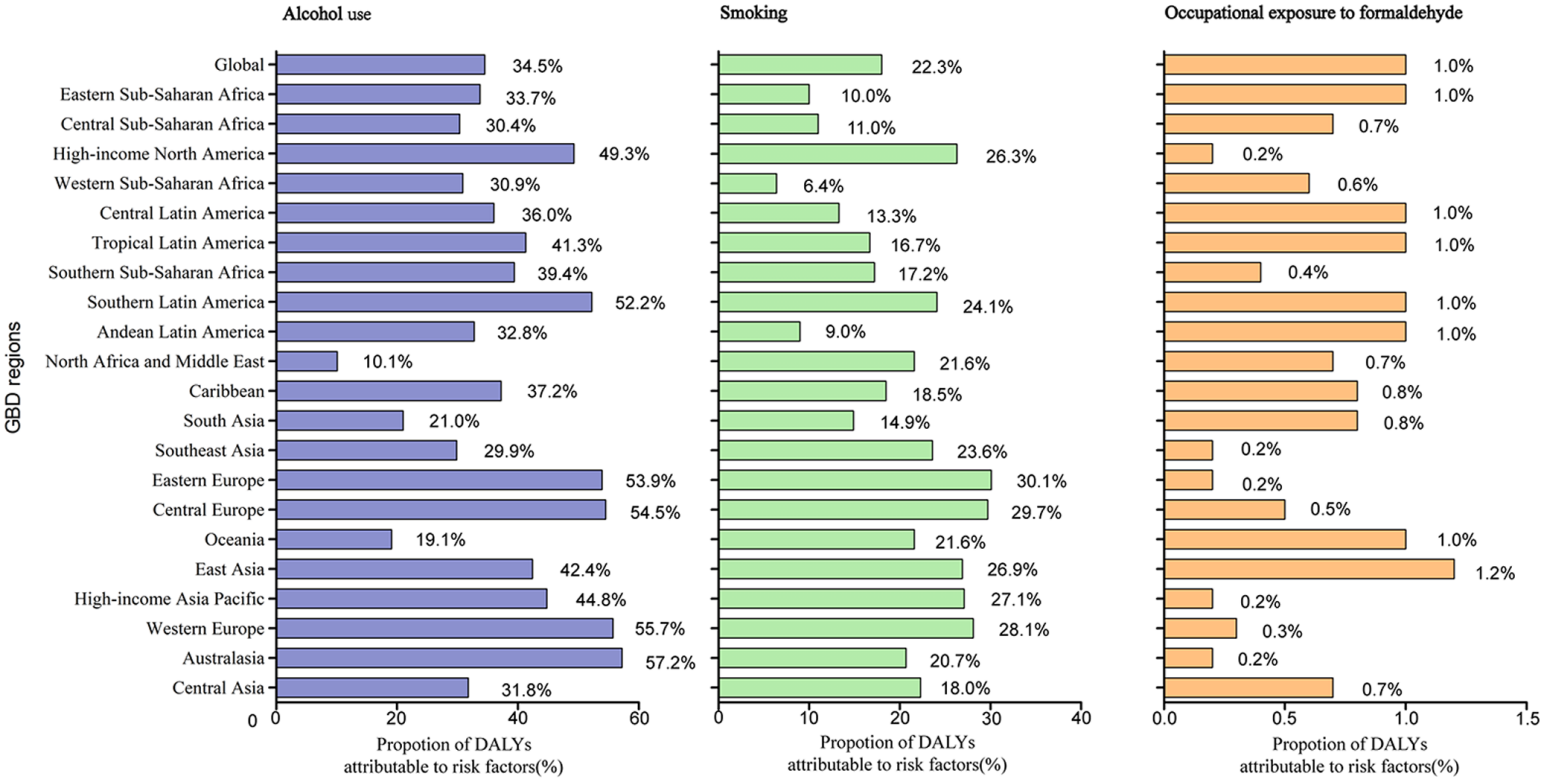
Percentage of NPC age‐standardized DALYs attributable to alcohol, smoking, occupational exposure to formaldehyde

The global DALYs were mainly attributable to three risk factors, including 34.5% (95% UI 27.4% to 41.2%) attributable to alcohol use, 22.3% (15.7% to 28.4%) to tobacco smoking and 1.0% (0.7% to 1.3%) to occupational exposure to formaldehyde. At the regional level, the impact of alcohol use was highest in Australasia (57.2%, 47.5% to 66.0%) and lowest in North Arica and the Middle East (10.1%, 6.5% to 13.6%). Likewise, the impact of tobacco smoking was highest in Eastern Europe (30.1%, 21.5% to 37.8%) and lowest in Western Sub‐Saharan Africa (6.4%, 3.7% to 9.4%) (Figure 4). The global pattern was different across age groups. The highest percentages of globally attributable DALYs were in the 50–54‐year age group for alcohol use, 65–69‐year age group for smoking, and 25–29‐year age group for occupational exposure to formaldehyde (Figure S22).

### 3.7 Predicted trends

#### 3.7.1 Global level

**FIGURE 5:**
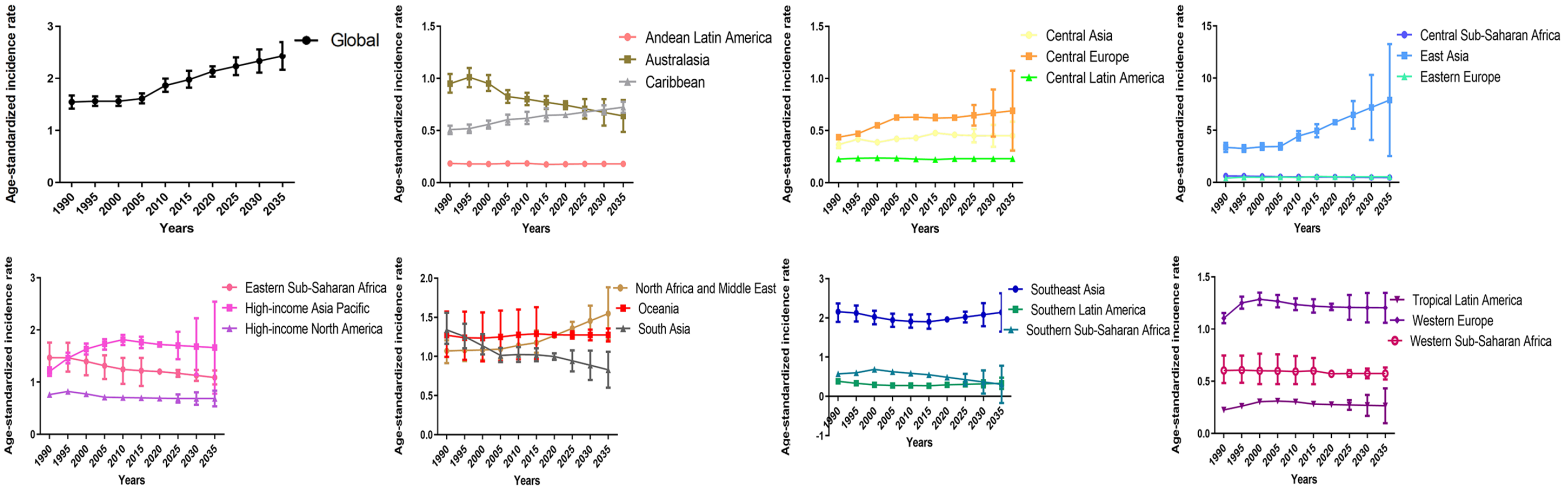
The temporal trends of age‐standardized incidence rates of NPC between 1990 and 2035 at the global level and regional level

The globally projected age‐standardized incidence rate is 2.4 (2.2 to 2.7) in 2035, which increased by 14.3% from 2019 to 2035 (Figure 5). The total number of new cases are 272,980 (187,195 to 358,765) in 2035, which increase by 54.7% from 2019 to 2035 (Table S9).

#### 3.7.2 Regional level

Figure 5 and Table S9 shows the trends in NPC incidence between 2019 and 2035. From 2019 to 2035, the age‐standardized incidence rates for NPC will increase in six of the 21 GBD regions, especially in East Asia (40.7% increase between 2019 and 2035), North Africa and Middle East (24.3% increase between 2019 and 2035), Southern Latin America (15.3% increase between 2019 and 2035). From 2019 to 2035, the age‐standardized incidence rates will decrease in 13 of the 21 GBD regions, especially in Southern Sub‐Saharan Africa (−38.5% decrease between 2019 and 2035), South Asia (−17.6% decrease between 2019 and 2035), and Australasia (−14.7% decrease between 2019 and 2035). The age‐standardized incidence rates in Central Latin America remain stable between 2019 and 2035.

#### 3.7.3 National level

Tables S7 and S10 presents the trends in predicted incidence for 204 countries in 2019 and 2035. There are 81 countries will experience decreasing trend for NPC incidence rates between 2019 and 2035, especially in South Africa (−51.6% decrease between 2019 and 2035), Dominican Republic (−49.6% decrease between 2019 and 2035), and Greenland (−40.3% decrease between 2019 and 2035). There are 88 countries will experience increasing trends in incidence rates between 2020 and 2035, especially in Malaysia (63.7% increase between 2019 and 2035), China (41.2% increase between 2019 and 2035), and Maldives (36.7% increase between 2019 and 2035).

## 4 DISCUSSION

The present research provided the latest comprehensive analysis of the global burden of NPC in 204 countries between 1990 and 2019, and its temporal trends and attributable risk factors. Meanwhile, we predict the NPC incidence rate from 2020 to 2035. These estimates aim to highlight the significant global burden of nasopharyngeal cancer.

Worldwide, an estimated 971,935 individuals lived with NPC, and 176,501 new cases occurred in 2019, contributing 71,610 deaths and 2,235,096 DALYs. The global age‐standardized incidence rates of NPC increased from 1990 to 2019; during the same period, the total counts of new cases, deaths, and DALYs due to NPC increased significantly as a result of population growth and aging.^13^, ^14^, ^15^ Meanwhile, the incidence rates of NPC continue to increase between 2019 and 2035. These estimates significantly highlighted the global burden of NPC. Additionally, compared with estimates of NPC based on GBD 2017 datasets,^16^ we added an analysis of attributable risk factors of NPC and found three potential modifiable risk factors, which were alcohol consumption, tobacco smoking, and occupational exposure to formaldehyde.

Historically, NPC has been considered a geographically distributed tumor in East Asian and Southeast Asian countries.^1^, ^2^, ^17^ East Asia made a significant contribution to the global number of patients with NPC, accounting for 72.4% of the total counts. In addition, East Asia has the highest age‐standardized incidence rate of NPC in 2019, and the rate in East Asia continues to increase between 2020 and 2035, followed by Southeast Asia and the high‐income Asia Pacific, whereas the lowest age‐standardized incidence rate of NPC was Andean Latin America, Central Latin America, and Tropical Latin America. These results indicated that the incidence of NPC had no connection with the degree of economic development. This geographical heterogeneity suggested that the differences in the incidence of NPC might be due to genetics, ethnicities, environmental factors, and lifestyle, which highlights the need for further research.

At the national level, the highest number of incidence cases, deaths, and DALYs were found in China, together with India, accounting for more than 50% of the global total numbers. This can be explained by those developing countries, including China and India, have a large population base.^18^, ^19^ Notably, we found that Singapore, as high‐income Asian Pacific country, had the highest age‐standardized incidence rate due to NPC, with low age‐standardized death and DALY rates. The high incidence rate together with the low death rate in developed countries emphasize the important role of medical level and resources in the survival and quality of life of NPC patients.^20^ It is worth mentioning that both age‐standardized death and DALY rates were highest in Greenland and Brunei Darussalam. Meanwhile, all the incidence, mortality, and DALY rates were high in Malaysia. The abovementioned data indicated that raising cancer awareness, detection, and control of risk factors were also important, as well as screening asymptomatic patients.^21^, ^22^, ^23^ Key interventions to decrease mortality due to NPC include improvements in radiotherapy, chemotherapy, and immunotherapy.^24^, ^25^, ^26^

As presented here and published in previous studies,^27^, ^28^ the global burden due to NPC was higher in males than in females across most of the age groups, which indicated that educational, preventive, and treatment approaches should be focused on these specific groups. We also analyzed the association between DALY rates and SDI at the regional and national levels. At the regional level, the expected pattern was nonlinear, peaking at an SDI value of approximately 0.45. At the national level, the expected pattern was nonlinear, with two peaks at SDI values of approximately 0.45 and 0.75. Most of the regions showed a decreasing trend in age‐standardized DALY rates in the study period, but we noticed that the DALY rates of many regions and countries were higher than expected levels across all SDI values, so countries at all development levels need to highlight preventive strategies.

Strategies to prevent NPC should focus on modifying risk factors and defining high‐risk groups by policy makers. Our research found three risk factors including alcohol use, cigarette smoking, and occupational exposure to formaldehyde. Previous studies reported that cigarette smoking was positively correlative with the development of NPC, which was highest among long‐term smokers.^29^, ^30^ Therefore, it is now well accepted that the government should prioritize the further implementation of anti‐smoking legislation,^31^, ^32^ followed by popularizing advertising bans and educational programs about smoking.^33^

It was reported that approximately 3.2% of all deaths in the world are related to the consumption of alcoholic beverages.^34^ Multiple studies reported that alcohol consumption was positively correlative with the development of NPC.^35^, ^36^ Therefore, tougher policies on reducing alcohol consumption will be more effective in reducing the corresponding health and life loss.^37^

Occupational exposure to formaldehyde occurs in many workplaces. However, the role of formaldehyde in the risk of NPC is controversial. A multicenter study supported the hypothesis that occupational exposure to formaldehyde increased the risk of NPC.^38^ Another study showed that no association was found between NPC and formaldehyde.^39^ Our findings showed that occupational exposure to formaldehyde had a low effect on the etiology of NPC, but we still supported the ban on activities where exposure to formaldehyde‐containing materials remains.

There were several limitations in our research. First, the quality and quantity of data from the GBD highly determined the accuracy of our study. Second, the Center for Disease Control and Prevention in some underdeveloped areas were not available to provide accurate and timely data. Third, the influence of race was not risk factor and treatment target of NPC, was not included in the GBD datasets.

This research systematically analyzed the global burden of nasopharyngeal cancer and its attributable risk factors from 1990 to 2019. Despite the decline in age‐standardized mortality and DALY rates globally, the age‐standardized incidence rate increased from 1990 to 2019, and continues to increase between 2020 and 2035, indicating that nasopharyngeal cancer remains a major health problem worldwide. Prevention strategies should focus on modifiable risk factors, especially among males in East Asia. Improvement of cancer awareness and early detection and treatment strategies by the governmental entities and ministers of health are still needed.

## CONFLICT OF INTEREST

The authors declare no conflict of interest.

## AUTHOR CONTRIBUTIONS

YXS and WWC designed the study, XJL analyzed the data, Heqing Li performed the statistical analyses, YXS and XJL drafted the initial manuscript. All authors reviewed the drafted manuscript for critical content.

## ETHICAL APPROVAL STATEMENT

Not applicable.

## PATIENT CONSENT STATEMENT

Not applicable.

## Supporting information


Figure S1‐S22

Data S1

Table S1‐S12

## Data Availability

The data were obtained through an online query tool from the website of IHME (http://ghdx.healthdata.org/).
