# Supplementary material for: The global, regional, national burden of nasopharyngeal cancer and its attributable risk factors (1990–2019) and predictions to 2035
Source: Cancer Med. 2022 Apr 27;11(22):4310–20. doi: 10.1002/cam4.4783 (PMC9678109; doi:10.1002/cam4.4783)
Supplement: Supplementary file 1 — Figure S1‐S22 [file CAM4-11-4310-s003.docx]

**FIGURE S1.** Age-standardized prevalence rate of NPC per 100000 population for both sexes, in 2019.


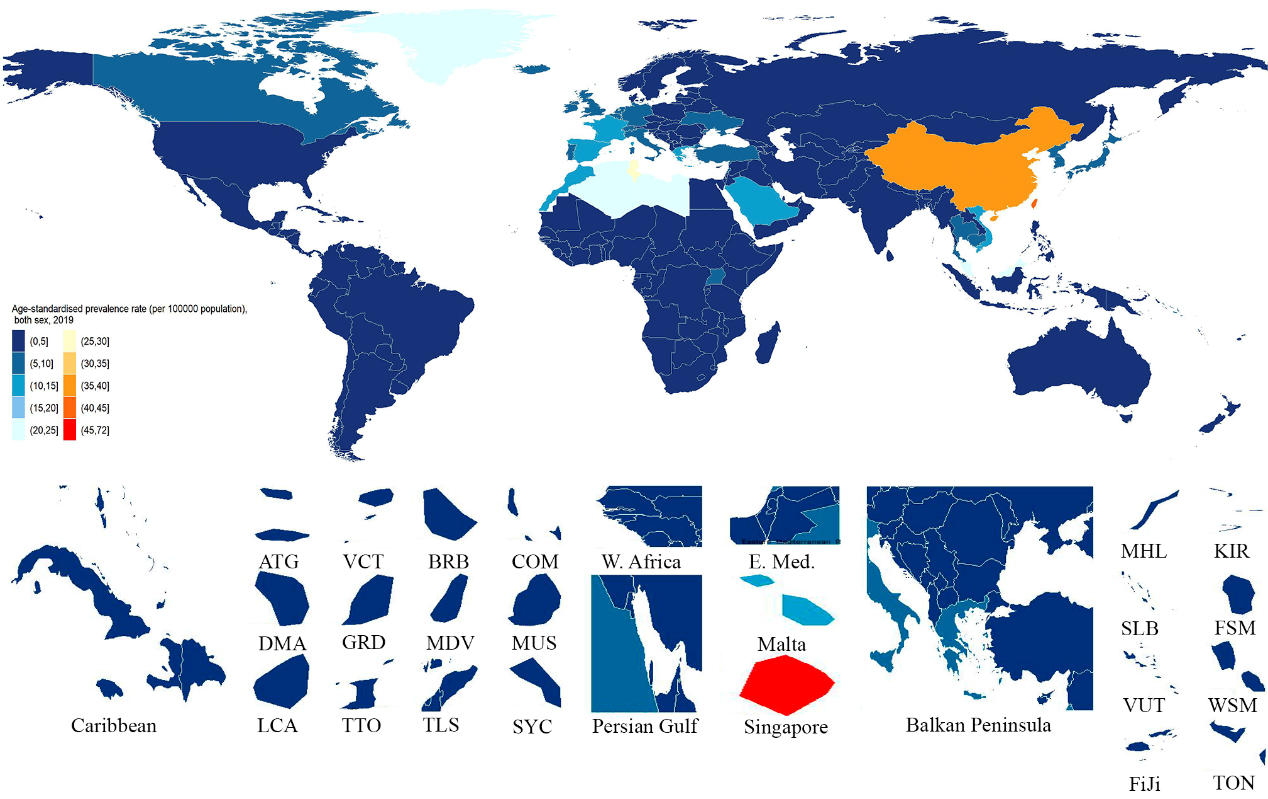


ATG, Antigua and Barbuda; BRB, Barbados; COM, Comoros; DMA, Dominica; FSM, Federated States of Micronesia; GRD, Grenada; KIR, Kiribati; LCA, Saint Lucia; MDV, Maldives; MHL, Marshall Islands; MUS, Mauritius; SLB, Solomon Islands; VCT, Saint Vincent and the Grenadines; SYC, Seychelles; TLS, Timor-Leste; TON, Tonga; TTO, Trinidad and Tobago; VUT, Vanuatu; WSM, Samoa.

**FIGURE S2** Incident counts of NPC for both sexes, in 2019.


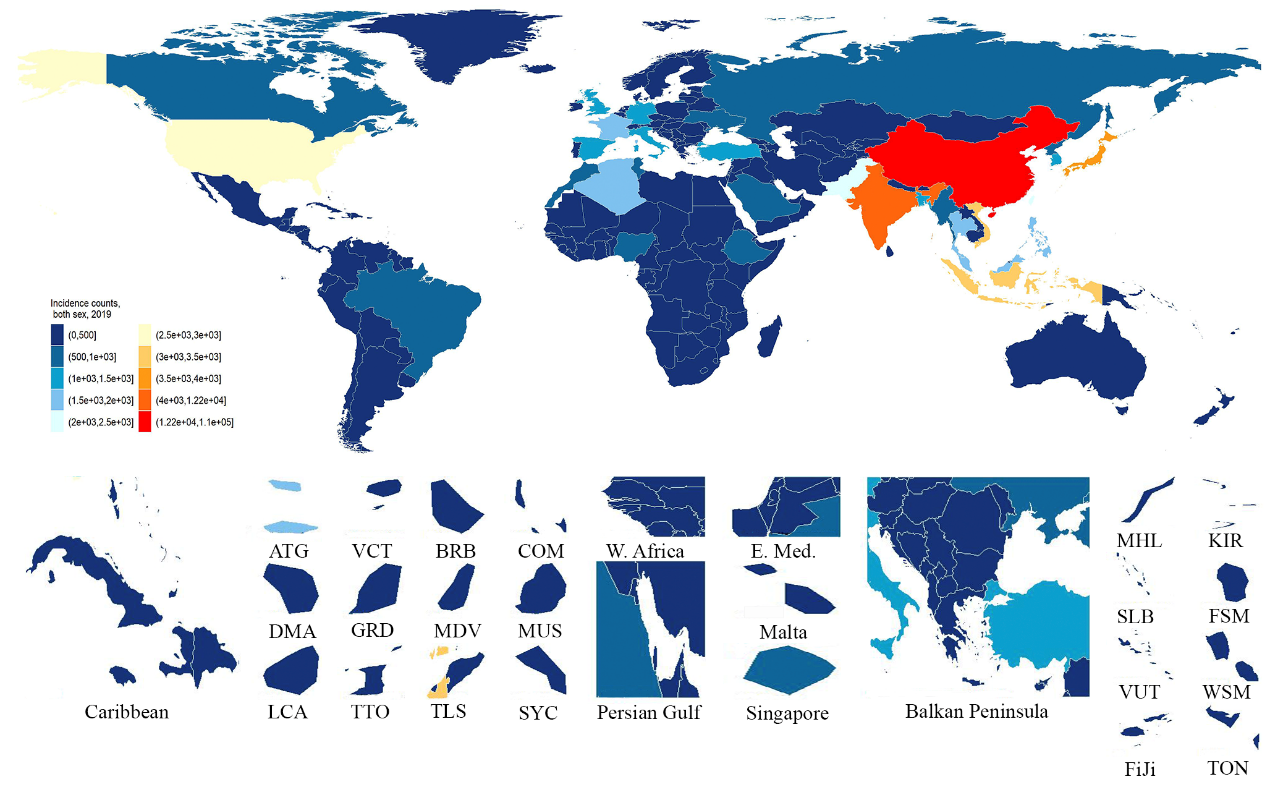


ATG, Antigua and Barbuda; BRB, Barbados; COM, Comoros; DMA, Dominica; FSM, Federated States of Micronesia; GRD, Grenada; KIR, Kiribati; LCA, Saint Lucia; MDV, Maldives; MHL, Marshall Islands; MUS, Mauritius; SLB, Solomon Islands; VCT, Saint Vincent and the Grenadines; SYC, Seychelles; TLS, Timor-Leste; TON, Tonga; TTO, Trinidad and Tobago; VUT, Vanuatu; WSM, Samoa.

**FIGURE S3** Death counts of NPC for both sexes, in 2019.


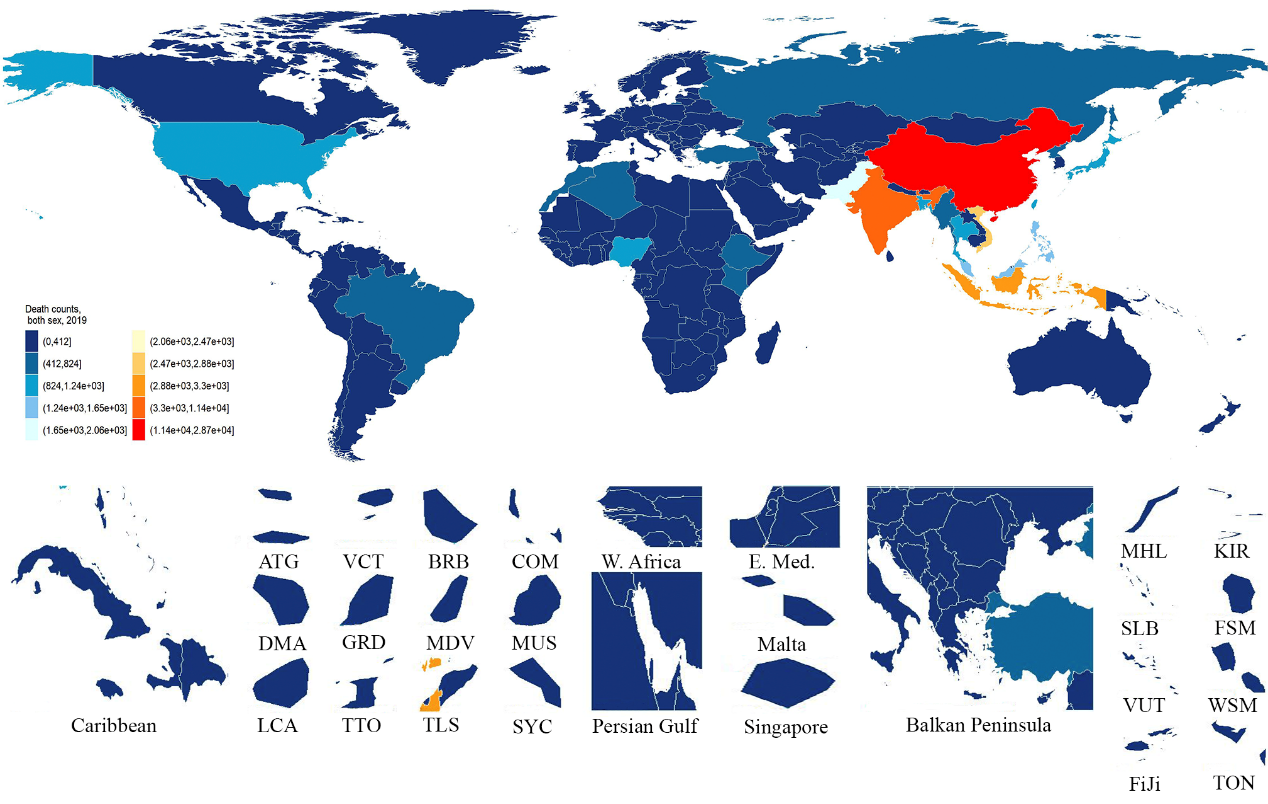


ATG, Antigua and Barbuda; BRB, Barbados; COM, Comoros; DMA, Dominica; FSM, Federated States of Micronesia; GRD, Grenada; KIR, Kiribati; LCA, Saint Lucia; MDV, Maldives; MHL, Marshall Islands; MUS, Mauritius; SLB, Solomon Islands; VCT, Saint Vincent and the Grenadines; SYC, Seychelles; TLS, Timor-Leste; TON, Tonga; TTO, Trinidad and Tobago; VUT, Vanuatu; WSM, Samoa.

**FIGURE S4** Prevalence counts of NPC for both sexes, in 2019.


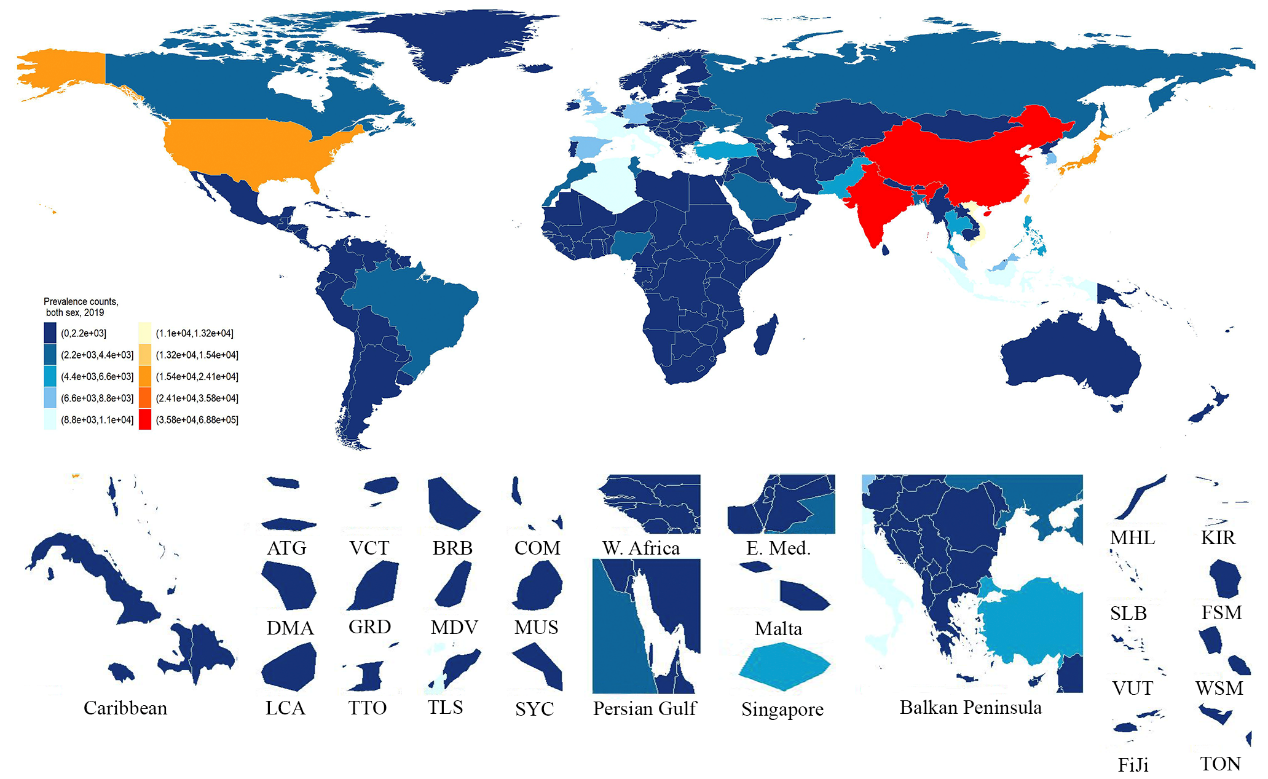


ATG, Antigua and Barbuda; BRB, Barbados; COM, Comoros; DMA, Dominica; FSM, Federated States of Micronesia; GRD, Grenada; KIR, Kiribati; LCA, Saint Lucia; MDV, Maldives; MHL, Marshall Islands; MUS, Mauritius; SLB, Solomon Islands; VCT, Saint Vincent and the Grenadines; SYC, Seychelles; TLS, Timor-Leste; TON, Tonga; TTO, Trinidad and Tobago; VUT, Vanuatu; WSM, Samoa.

**FIGURE S5.** The 204 countries and territories distribution by SDI quintile, 2019.


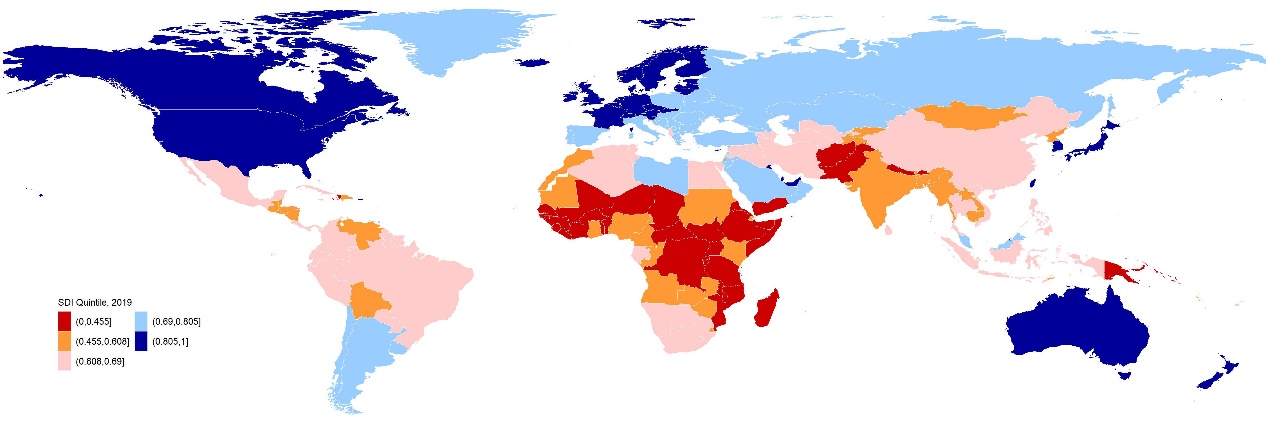


SDI, Socio-Demographic Index.

**FIGURE S6** Age-standardized incidence (A), death (B) and DALY (C) rates of NPC per 100000 population for 21 Global Burden Disease regions by sex, 2019.

**
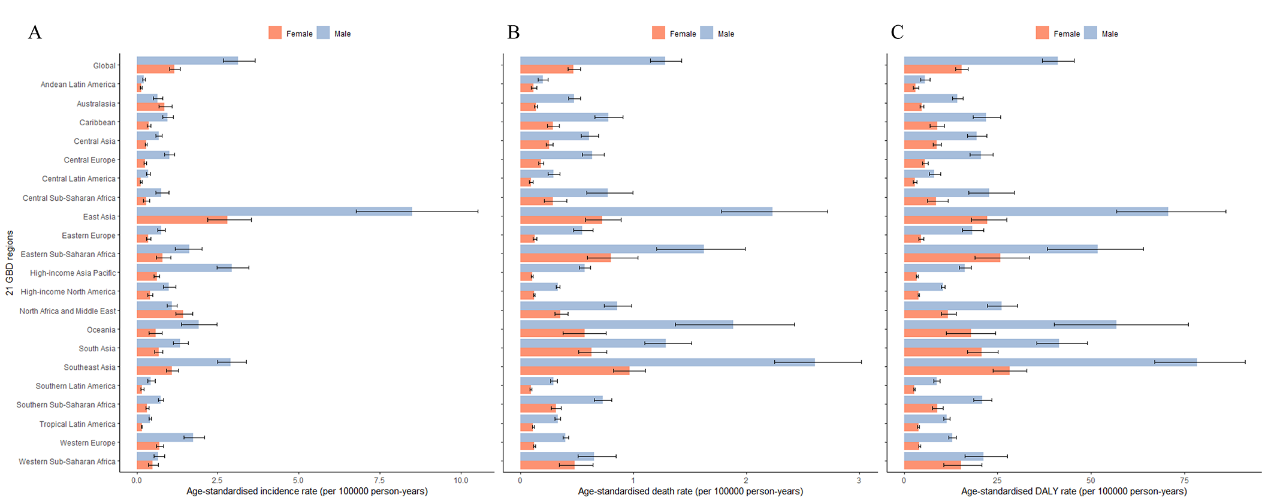
**

Error bars indicate the 95% uncertainty intervals (95% UI) for Age-standardized incidence (A), death (B) and DALYs (C).

DALYs, disability adjusted life years.

**FIGURE S7** Incident counts (A), death counts(B) and DALYs (C) of NPC for 21 Global Burden Disease regions by sex, 2019.


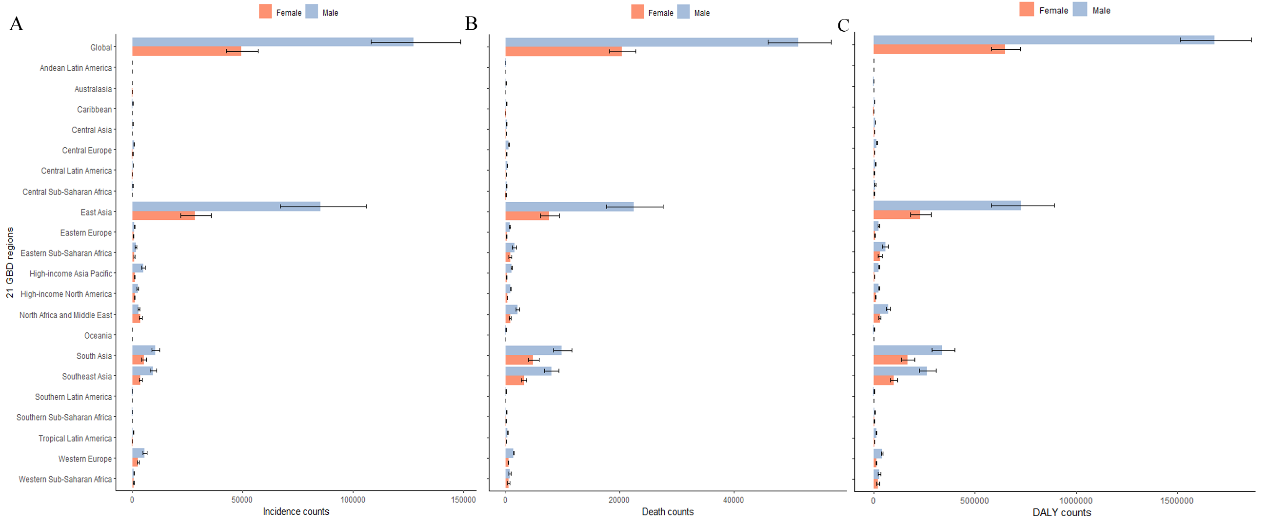


Error bars indicate the 95% uncertainty intervals (95% UI) for incidence (A), death (B) and DALYs (C).

DALYs, disability adjusted life years.

**FIGURE S8** Age-standardized YLD rates of NPC per 100000 population for 21 Global Burden Disease regions by sex, 2019.


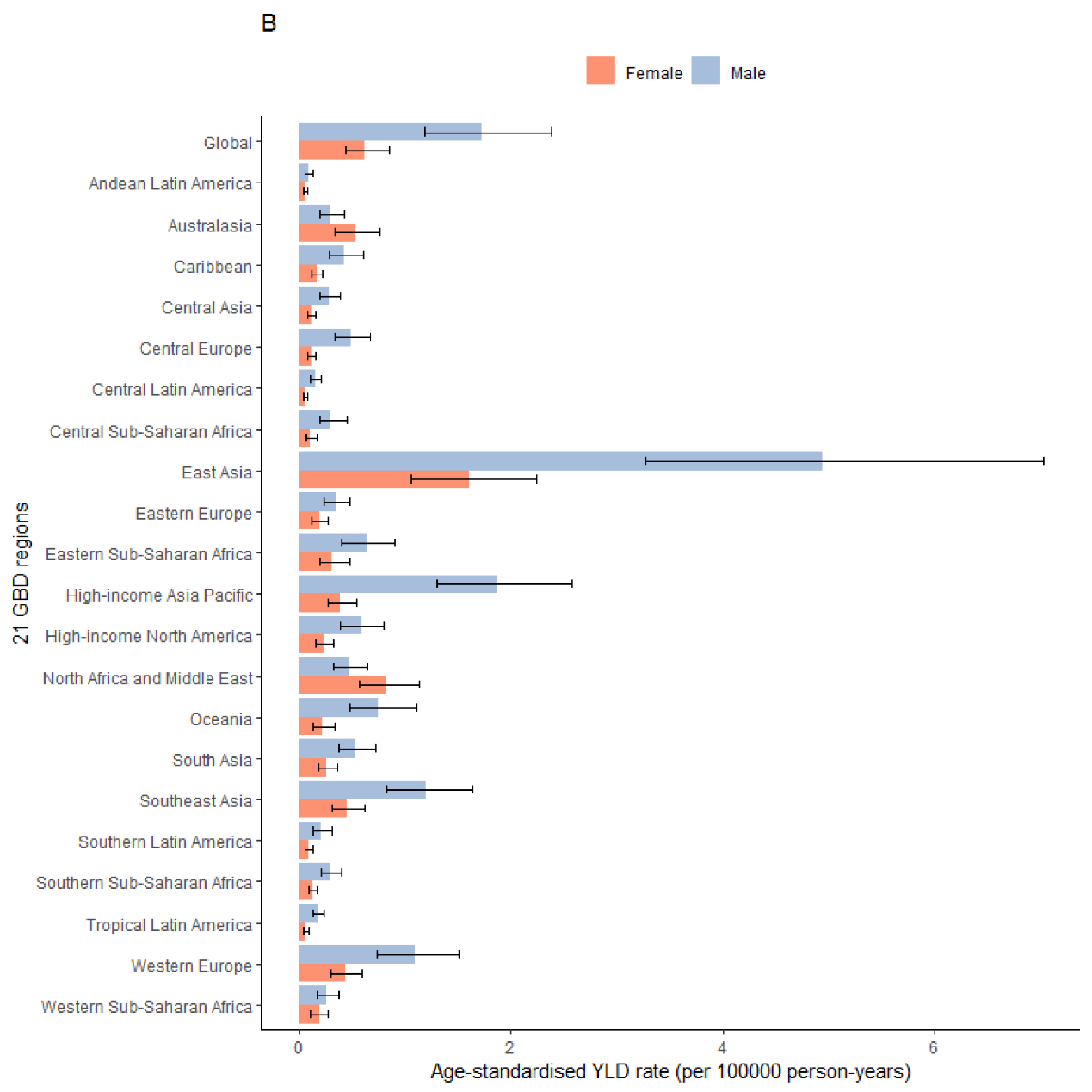


Error bars indicate the 95% uncertainty intervals (95% UI) for YLDs.

YLDs, years lived with disability.

**FIGURE S9** Age-standardized YLL rates of NPC per 100000 population for 21 Global Burden Disease regions by sex, 2019.


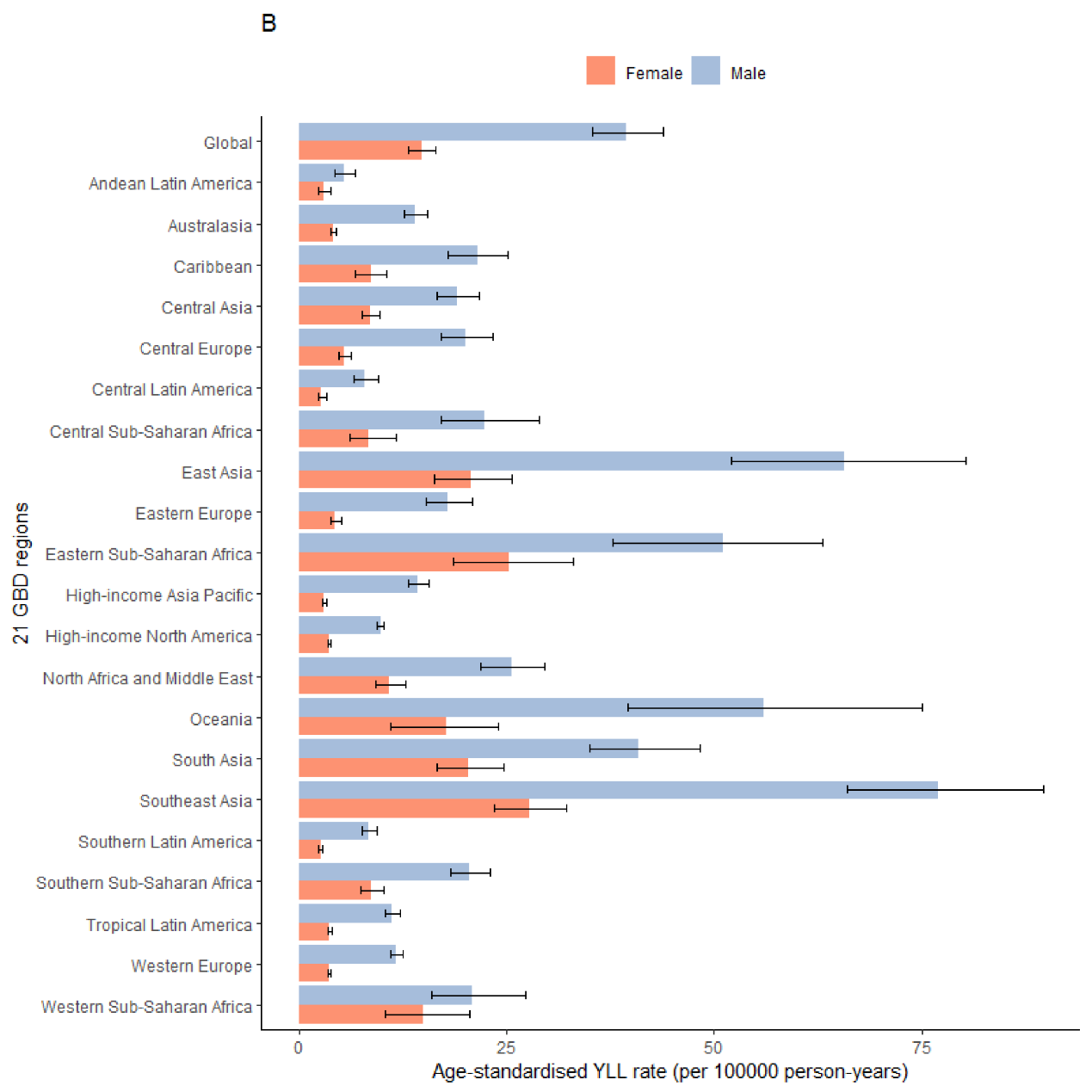


Error bars indicate the 95% uncertainty intervals (95% UI) for YLLs.

YLLs, years of life lost.

**FIGURE S10** Age-standardized prevalence rates of NPC per 100000 population for 21 Global Burden Disease regions by sex, 2019.


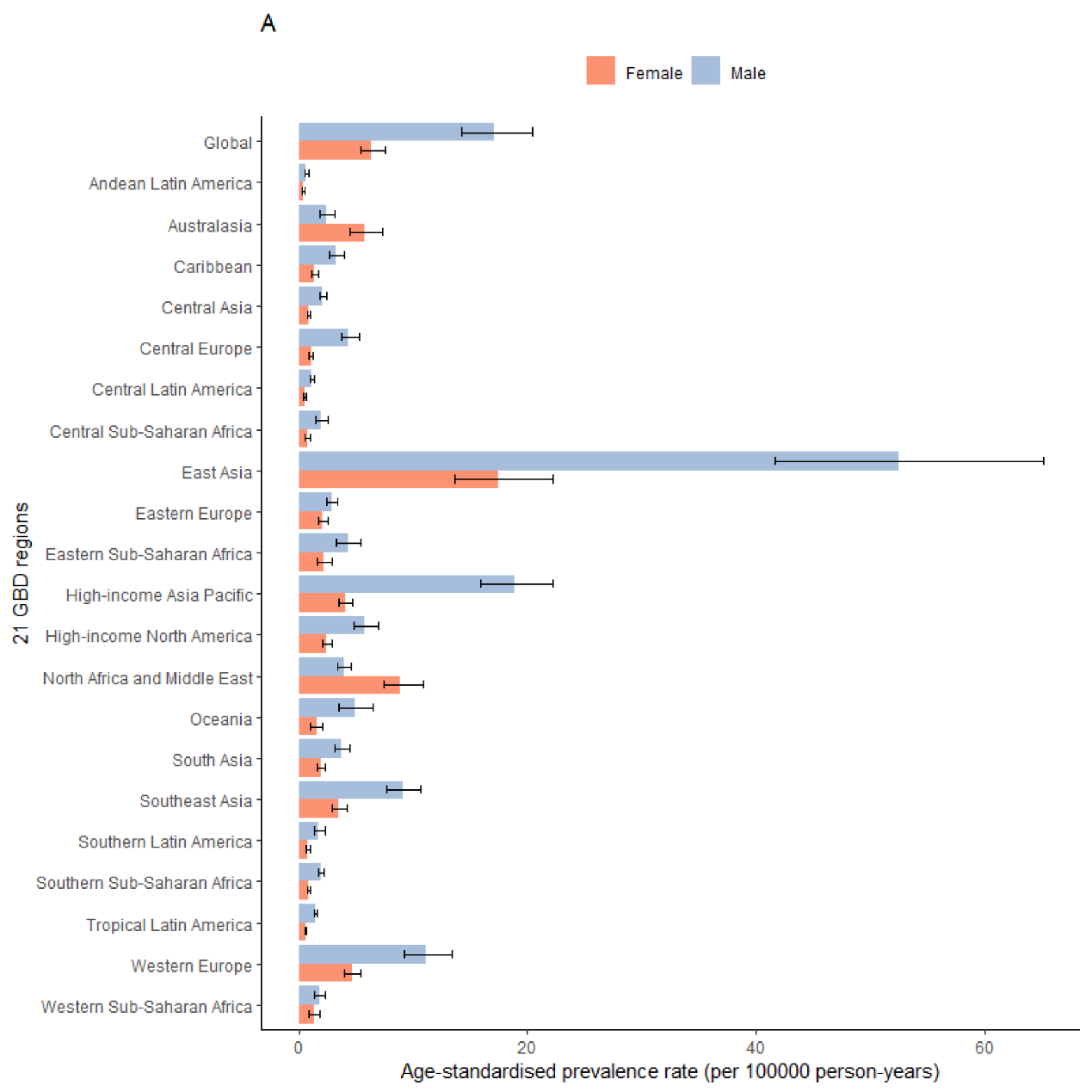


Error bars indicate the 95% uncertainty intervals (95% UI) for prevalence.

**FIGURE S11** Global counts and age-standardized prevalence rates of NPC per 100000 population by age and sex, 2019.


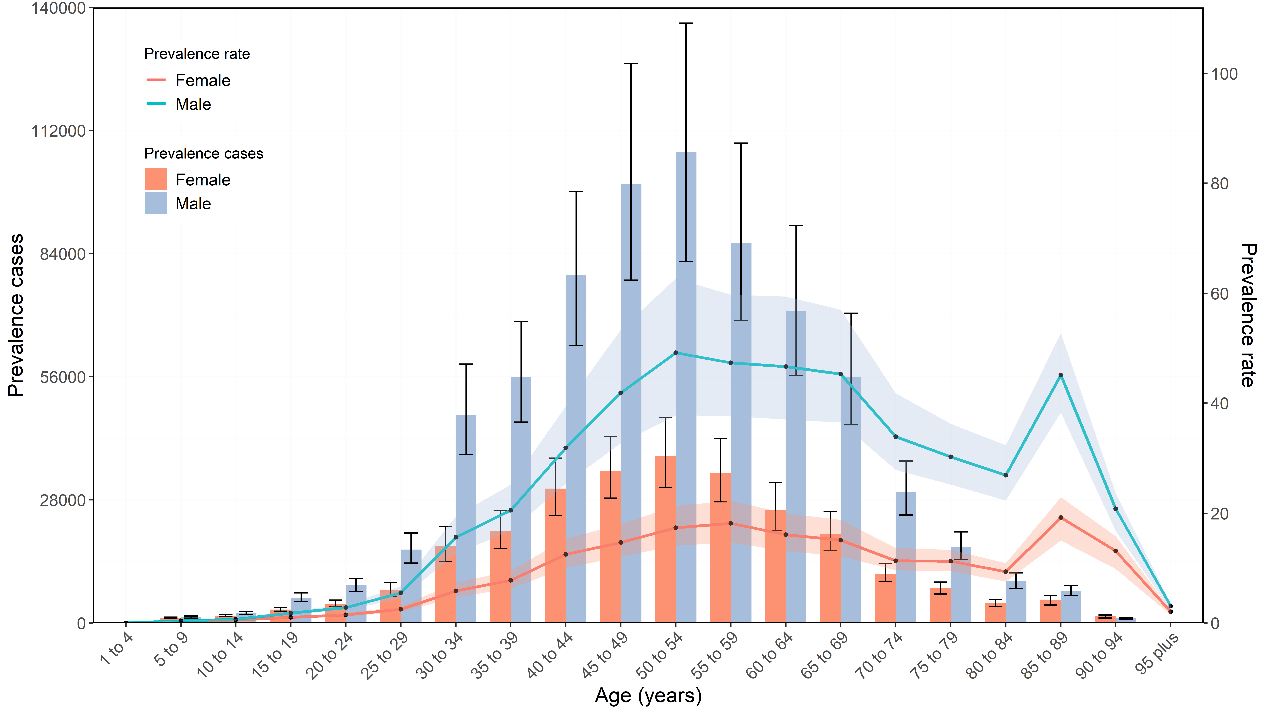


Error bars indicate the 95% uncertainty intervals (95% UI) for prevalence. Shading indicates the upper and lower limits of the 95% UI.

**FIGURE S12** Global counts and age-standardized YLD rates of NPC per 100000 population by age and sex, 2019.


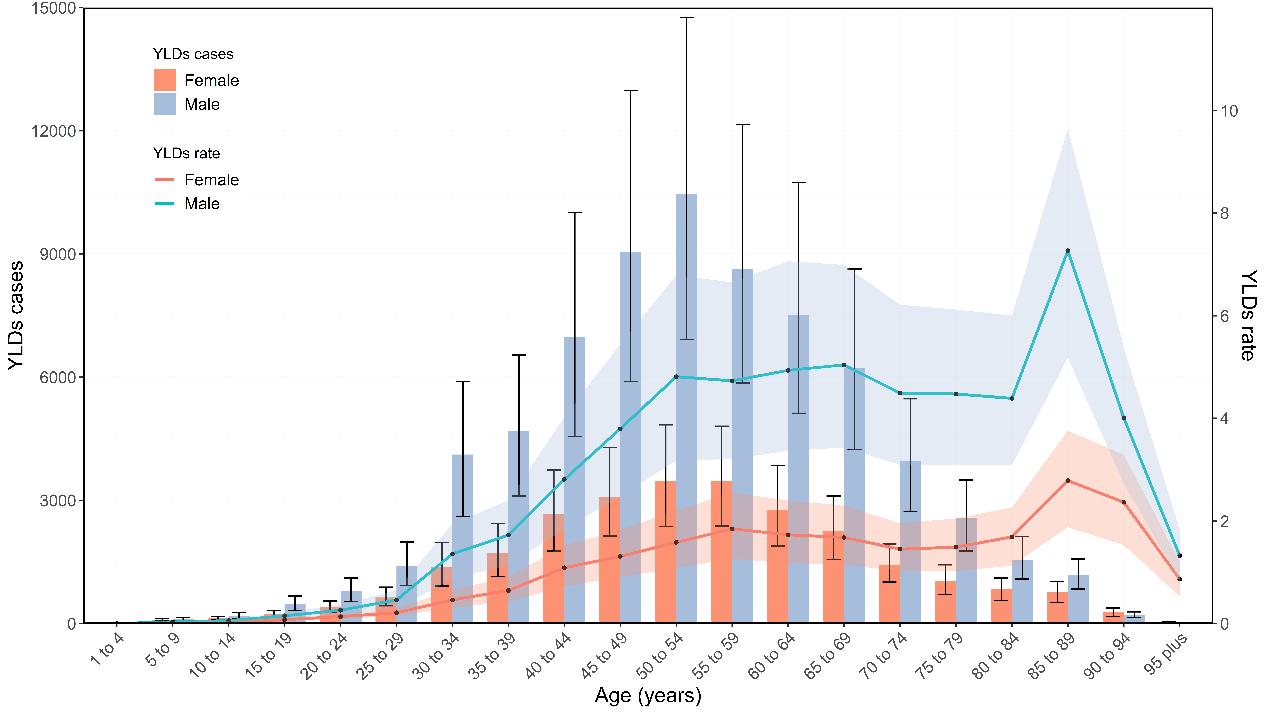


Error bars indicate the 95% uncertainty intervals (95% UI) for YLDs. Shading indicates the upper and lower limits of the 95% UI.

YLDs, years lived with disability.

**FIGURE S13** Global counts and age-standardized YLL rates of NPC per 100000 population by age and sex, 2019


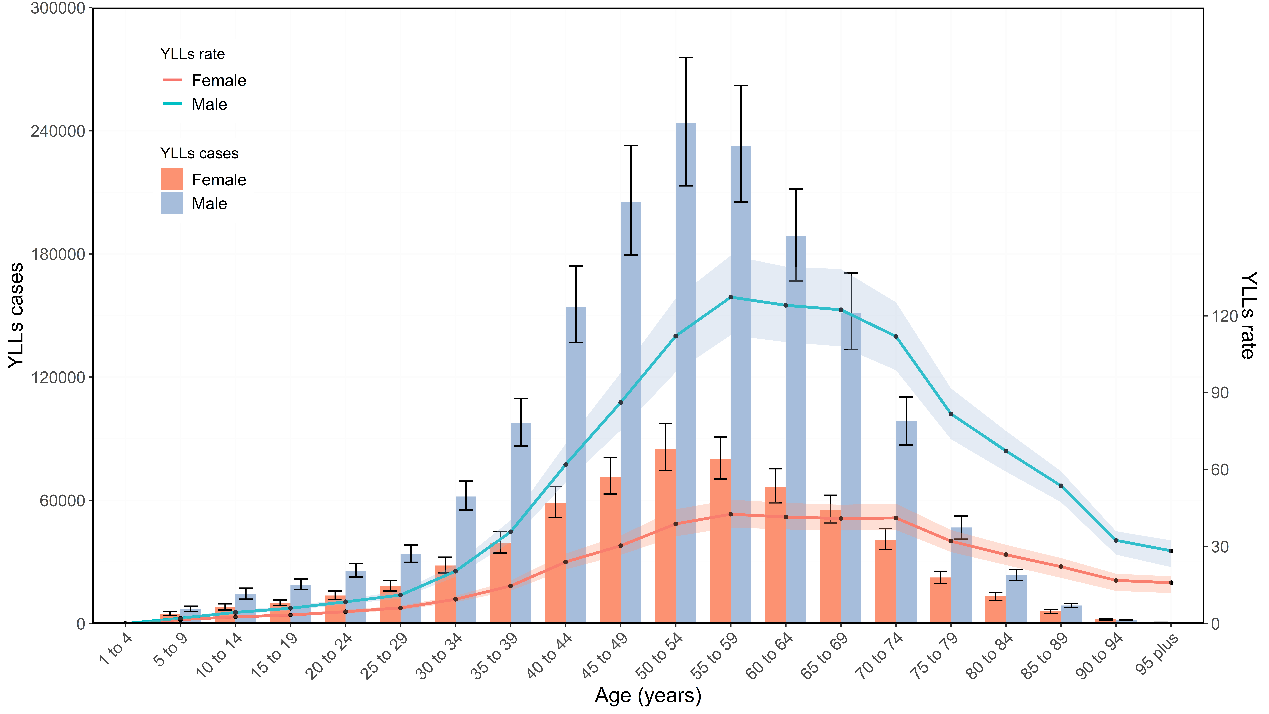


Error bars indicate the 95% uncertainty intervals (95% UI) for YLLs. Shading indicates the upper and lower limits of the 95% UI.

YLLs, years of life lost.

**FIGURE S14** The percentage change in age-standardized incidence (A), death (B) and DALY (C) rates of NPC per 1000000 by sex for 21 Global Burden of Disease regions, 1990-2019.


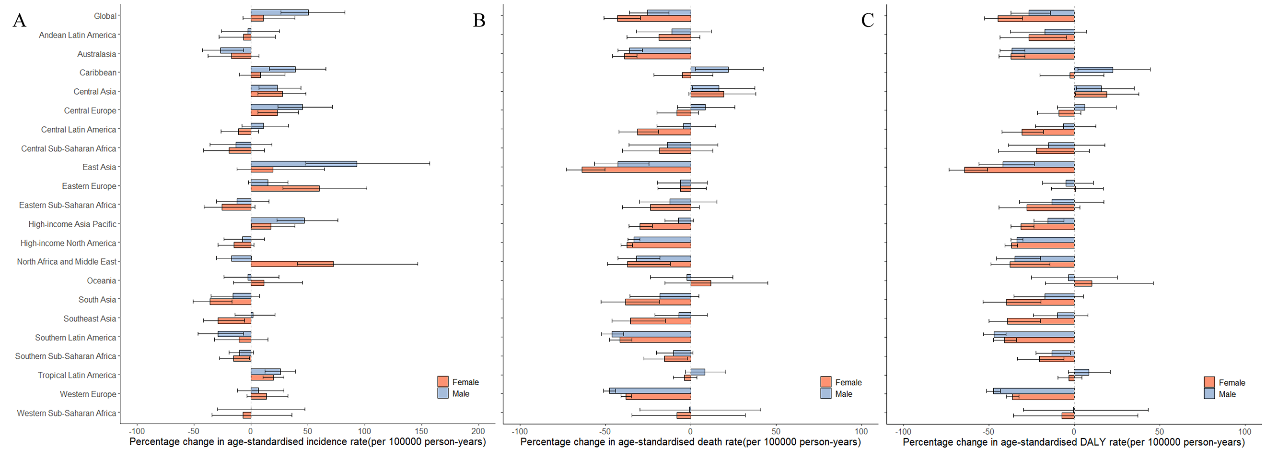


Error bars indicate the 95% uncertainty intervals (95% UI) for Age-standardized incidence (A), death (B) and DALYs (C).

DALYs, disability adjusted life years.

**FIGURE S15** The percentage change in age-standardized prevalence rate of NPC per 1000000 by sex for 21 Global Burden of Disease regions, 1990-2019.


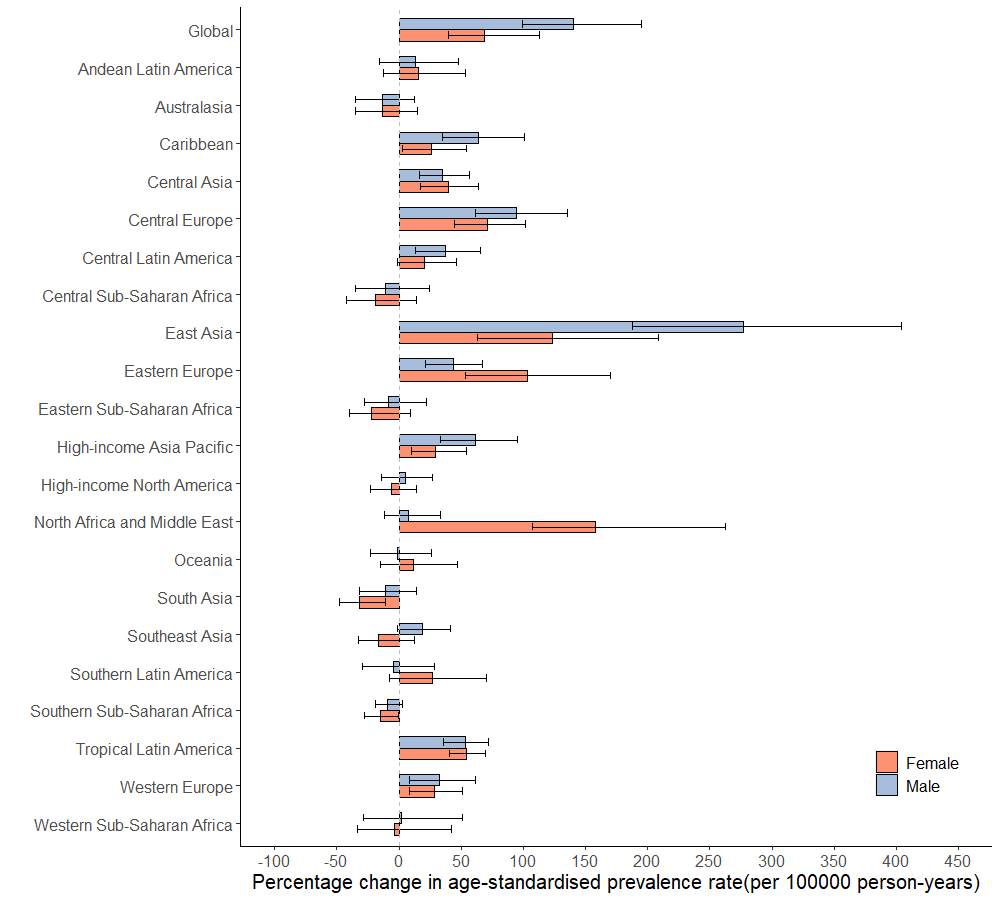


Error bars indicate the 95% uncertainty intervals (95% UI) for prevalence.

**FIGURE S16** The percentage change in age-standardized YLD rates of NPC per 1000000 by sex for 21 Global Burden of Disease regions, 1990-2019.


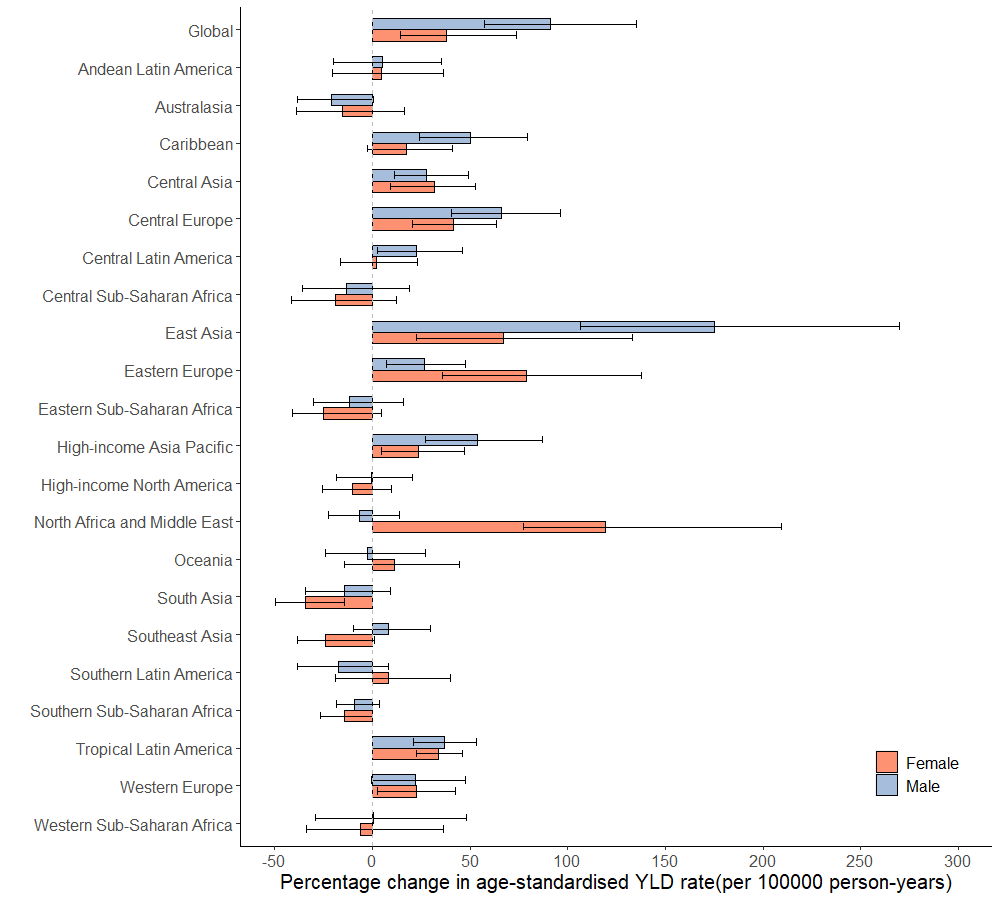


Error bars indicate the 95% uncertainty intervals (95% UI) for YLDs.

YLDs, years lived with disability.

**FIGURE S17** The percentage change in age-standardized YLL rates of NPC per 1000000 by sex for 21 Global Burden of Disease regions,1990-2019.


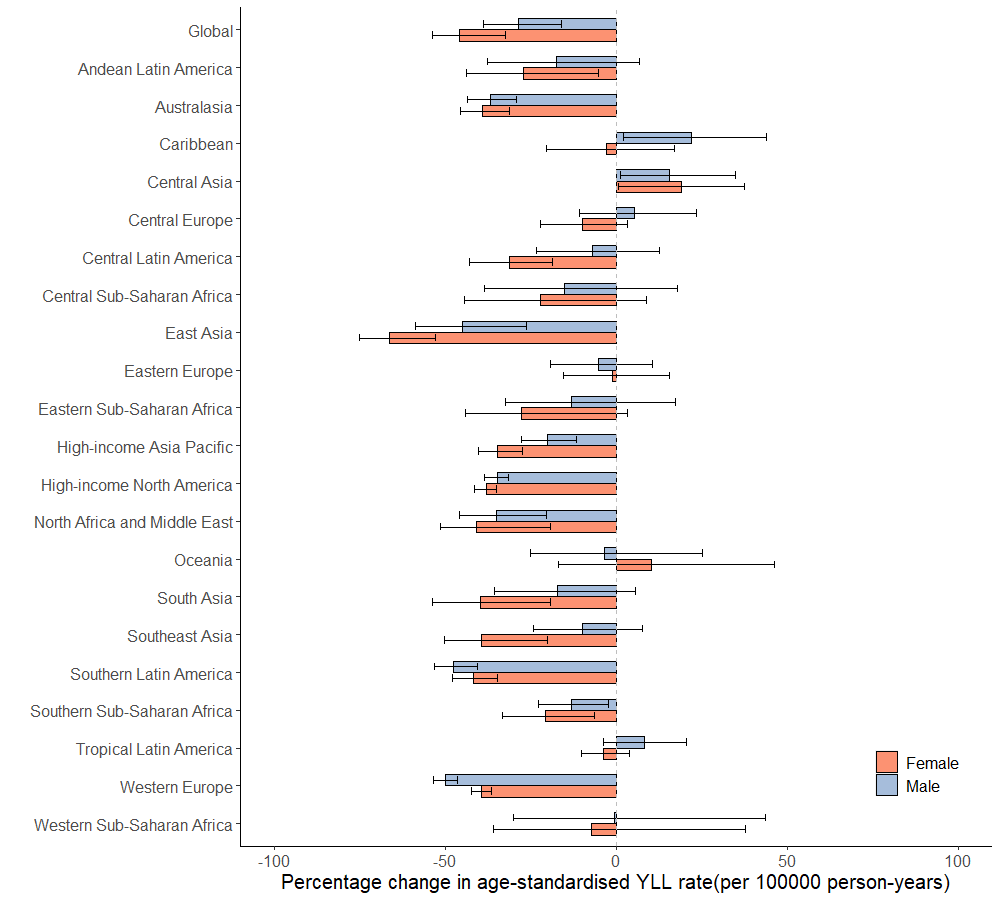


Error bars indicate the 95% uncertainty intervals (95% UI) for YLLs.

YLLs, years of life lost.

**FIGURE S18** Age-standardized YLD rates of NPC for 204 countries and territories by SDI, 2019


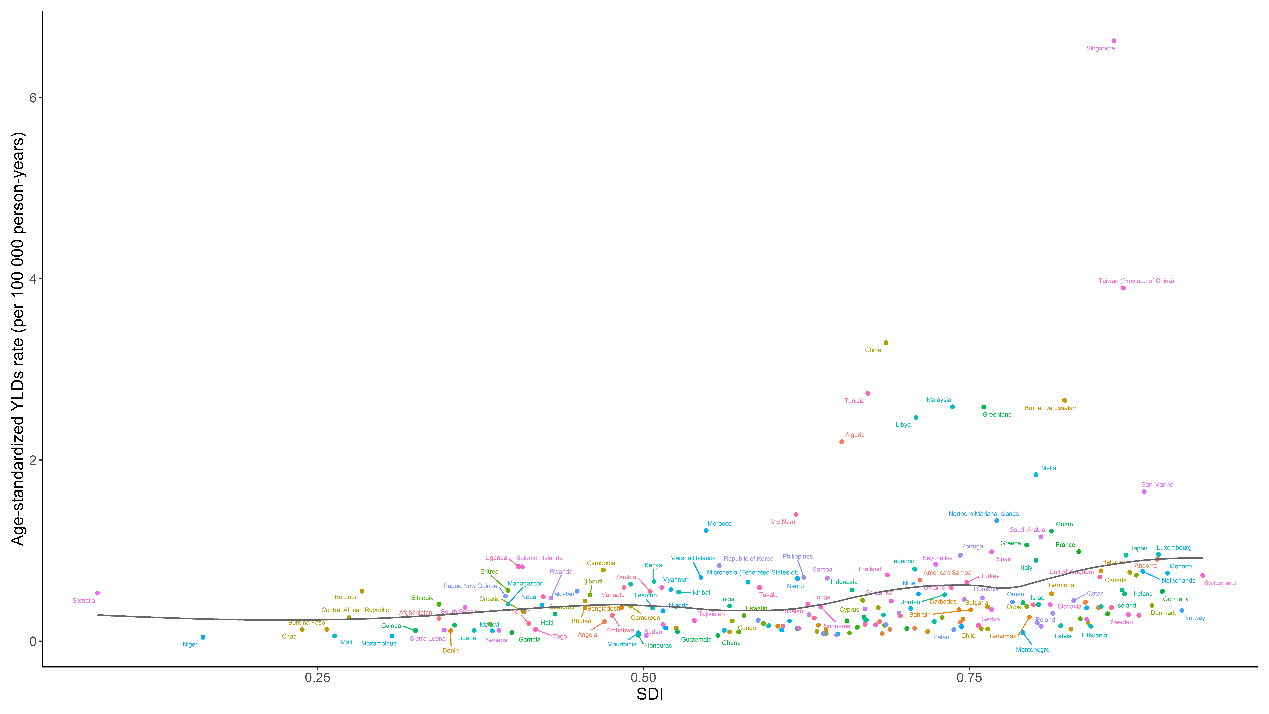
Black line represents the expected age-standardized YLD rates of nasopharyngeal cancer based on SDI.

SDI=Socio-demographic Index.

YLDs, years lived with disability.

**FIGURE S19** Age-standardized YLL rates of NPC for 204 countries and territories by SDI, 2019.


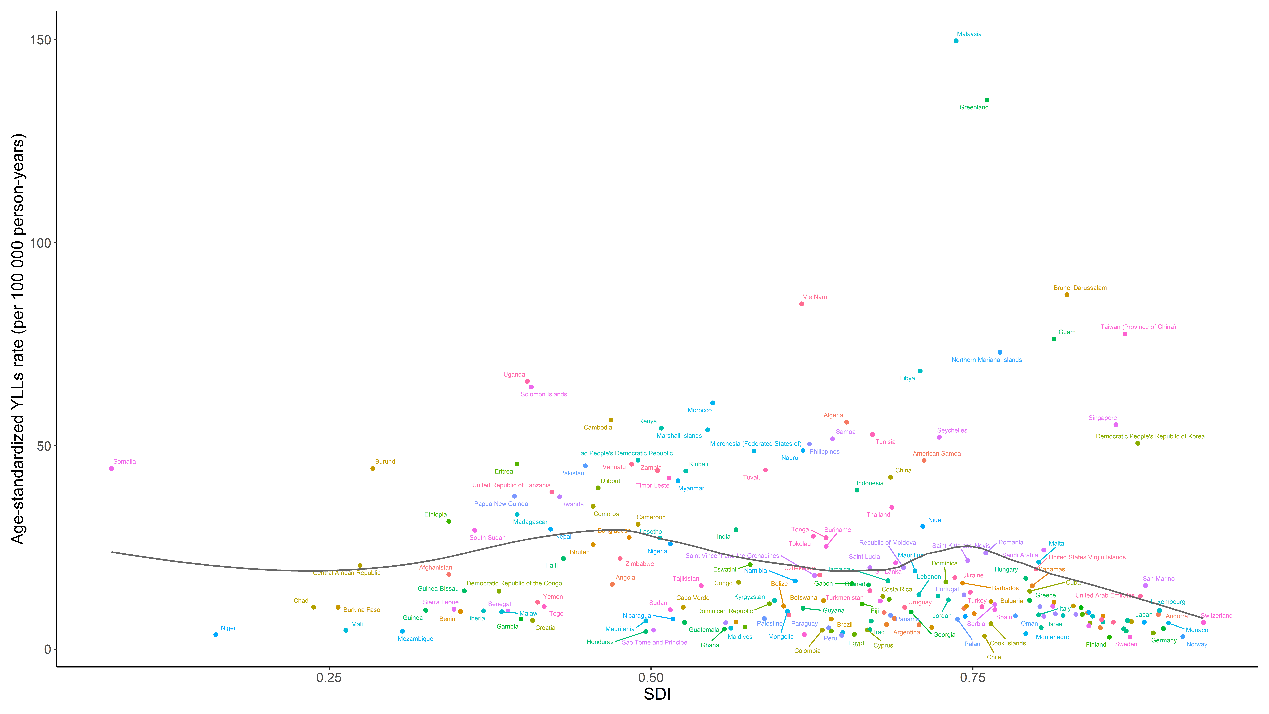


Black line represents the expected age-standardized YLL rates of nasopharyngeal cancer based on SDI. SDI=Socio-demographic Index.

YLLs, years of life lost.

**FIGURE S20** Age-standardized YLD rates of NPC for 21Global Burden of Disease region by SDI, 1990-2019.


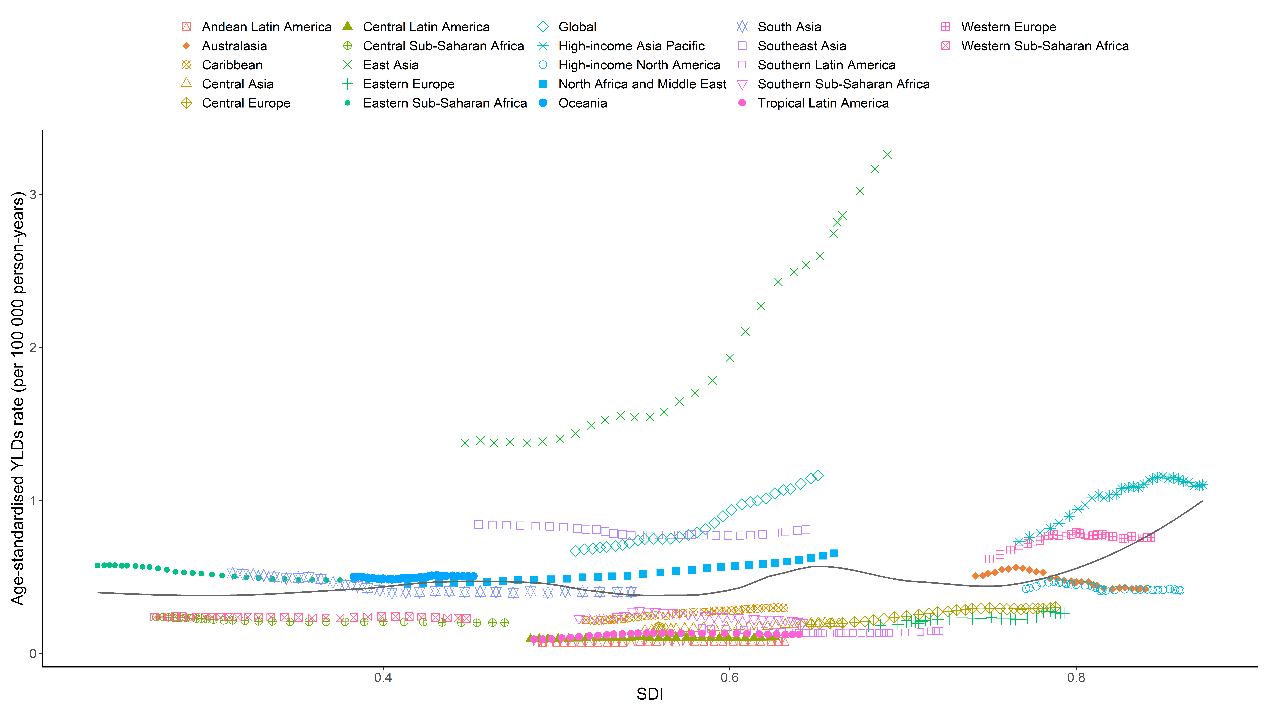


Black line represents the expected age-standardized YLD rates of nasopharyngeal cancer based on SDI. For each region, points from the left to right depict estimates from each year from 1990 to 2019.

SDI, Socio-demographic Index; YLDs, years lived with disability.

**FIGURE S21** Age-standardized YLL rates of NPC for 21Global Burden of Disease regions by SDI, 1990-2019.


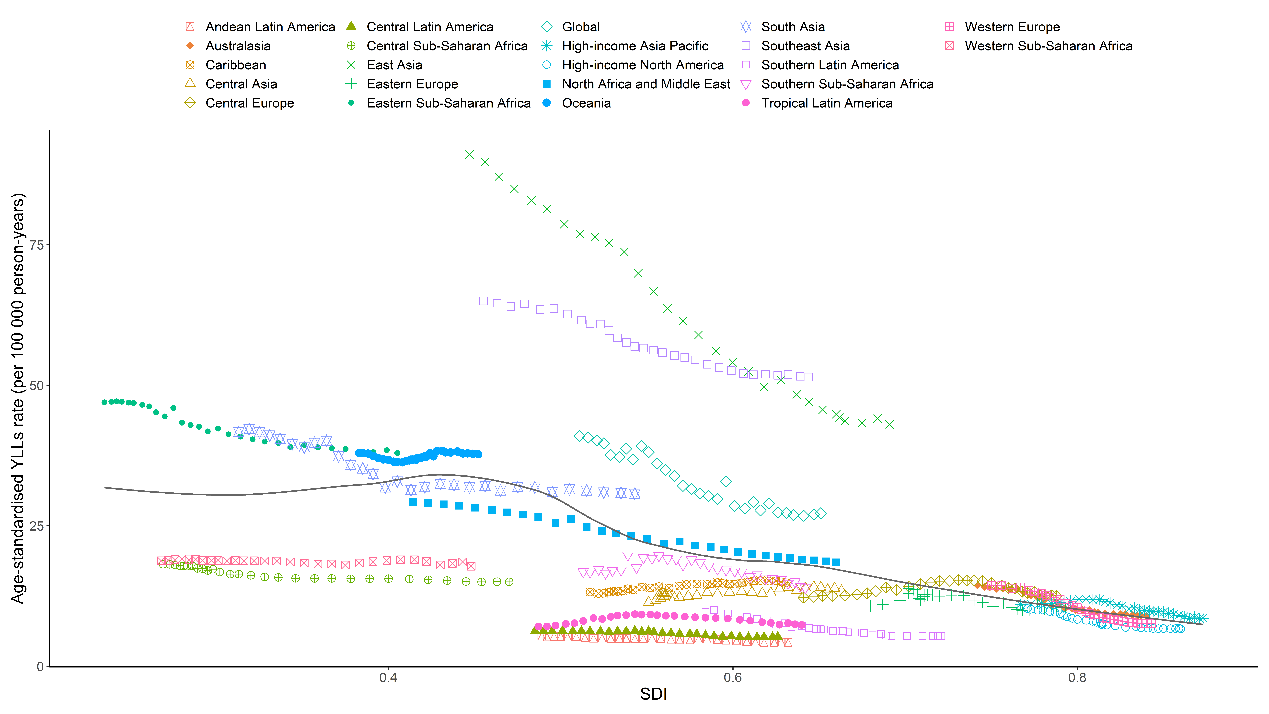


Black line represents the expected age-standardized YLL rates of nasopharyngeal cancer based on SDI. For each region, points from the left to right depict estimates from each year from 1990 to 2019.

SDI, Socio-demographic Index; YLLs, years of life lost.

**FIGURE S22** Percentage of NPC DALYs attributable to alcohol use, smoking, occupational exposure to formaldehyde by age groups, 2019.


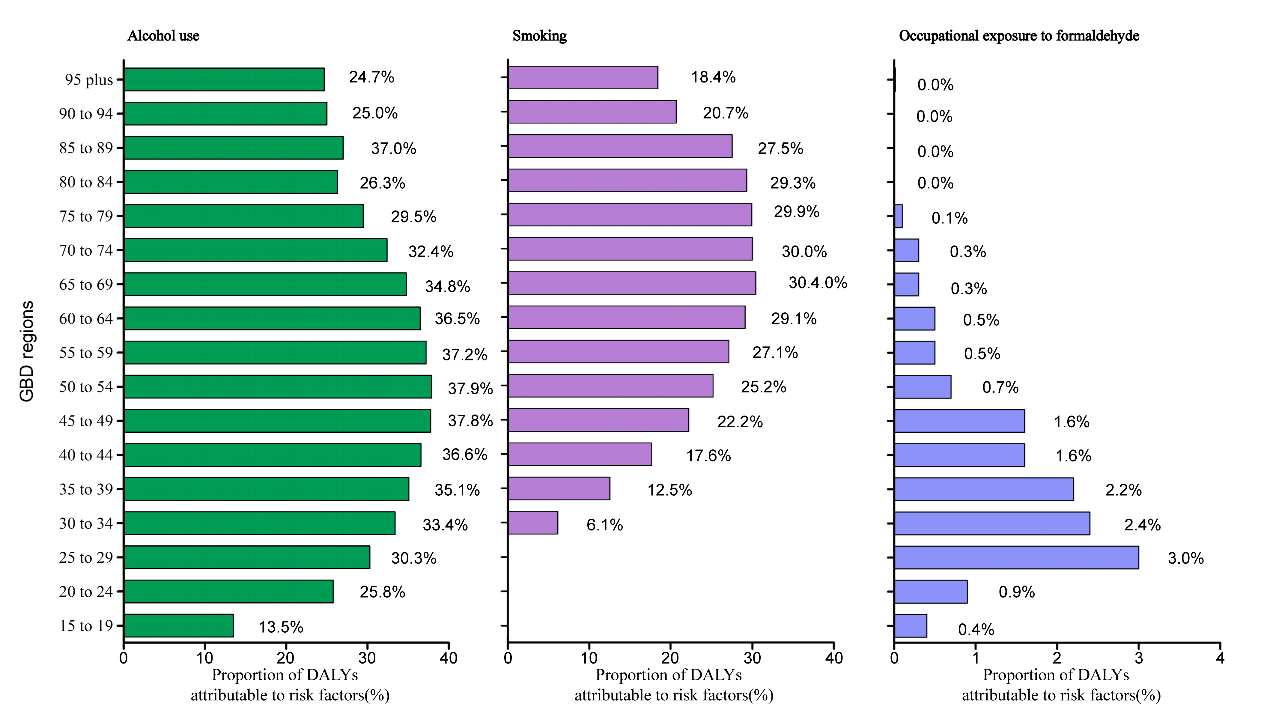


DALYs, disability adjusted life years.
