# Supplementary material for: The global, regional, national burden of nasopharyngeal cancer and its attributable risk factors (1990–2019) and predictions to 2035
Source: Cancer Med. 2022 Apr 27;11(22):4310–20. doi: 10.1002/cam4.4783 (PMC9678109; doi:10.1002/cam4.4783)
Supplement: Supplementary file 2 — Data S1 [file CAM4-11-4310-s002.docx]

**Supplementary methods**

**GBD overview**

GBD 2019 produced estimates for 204 countries and territories that were grouped into 21 regions and seven super-regions. For GBD 2019, nine countries and territories (Cook Islands, Monaco, San Marino, Nauru, Niue, Palau, Saint Kitts and Nevis, Tokelau, and Tuvalu) were added, such that the GBD location hierarchy now includes all WHO member states.

For GBD 2019, we have also defined locations as standard locations and non-standard locations. Standard GBD locations are defined as the set of all sub-nationals belonging to countries where data quality is high and with populations over 200 million, in addition to all other countries. Standard locations include the sub-nationals for China, India, the United States, and Brazil, but not Indonesia; China, India, the United States, and Brazil are also included at the country level. All other countries with subnational estimates are defined as non-standard locations.

A complete set of risk-specific exposures, relative risks (RRs), theoretical minimum-risk exposure levels (TMRELs), and population attributable fractions (PAFs) were computed for the years 1990-2019.

**Data sources**

In the current study, the data extracted from the GBD 2019 were estimated from vital registration (VR) systems, sample vital registration, verbal autopsy[1]. Information about the data sources used for each location in this study can be found on the GBD 2019 Data Input Sources Tool website.

**Age groups**

Following GBD 2019 studies, mortality and population are estimated for 23 age groups: early neonatal (0-6 days), late neonatal (7-27 days), post-neonatal (28-365 days), 1-4 years, 5-9 years, every 5-year age group up to 95 years, and 95 years and older. Age-specific fertility is estimated for 5-year age groups between ages 10 years and 54 years.

**Mortality Estimates**

We derived mortality estimates from the data source described above and, when necessary, registry incidence data were multiplied by the corresponding, independently modelled, mortality-to-incidence ratios (MIRs) to produce mortality estimates. MIRs were modelled using locations where same-year cancer mortality and incidence data were available. These MIRs model started with a linear-step mixed-effects model with logit link functions, with Healthcare Access and Quality Index, age, and sex as covariates. The resulting estimates were them smoothed over space and time, and adjusted with spatiotemporal Gaussian process regression[2]. The nasopharyngeal cancer estimated mortality was combined with observed mortality (from vital registration and verbal autopsy) and entered into the Cause of Death Ensemble Model (CODEm)[3]. The CoDCorrect algorithm was used to adjust estimated single causes of mortality to ensure that all single causes sum to the all-cause mortality estimation[1, 4, 5].

**Nonfatal Estimates**

The nasopharyngeal cancer survival data were collected and correlated with MIRs to generate prevalence. To estimate the YLDs, 10-year cancer prevalence was classified into four sequelae and multiplied the prevalence by corresponding disability weights (DWs): diagnosis and treatment, remission, disseminated and metastatic, and terminal phase. The durations of the four prevalence phases for Nasopharyngeal cancer were 5.3 months of diagnosis and treatment[6], 13.19 months of disseminated and metastatic disease^13^, and 1 month of terminal phase. Remission durations were calculated on the basis of the remainder of time after attributing other sequelae. Disability Weights are measured on a scale from 0 to 1, where 0 is a state of full health and 1 is death. Diagnosis and primary therapy phase of nasopharyngeal cancer has a disability weight of 0.288 (95% UI 0.193-0.399); Metastatic phase of nasopharyngeal cancer has a disability weight of 0.451 (95% UI 0.307-0.6); Terminal phase of nasopharyngeal cancer has a disability weight of 0.54 (95% UI 0.377-0.687); Controlled phase of nasopharyngeal cancer has a disability weight of 0.049 (95% UI 0.031-0.072)[7]. YLLs were computed by multiplying the age-specific estimates of number of deaths with a standard life expectancy at corresponding age. DALYs were the sum of YLDs and YLLs. The contributions of YLDs and YLLs to nasopharyngeal cancer DALYs were 3% and 97%, respectively. One DALY can be interpreted as 1 year of “healthy life” lost[8].

The age-standardized rates and estimated annual percentage change were used to quantify the nasopharyngeal cancer burden trends. It is necessary to standardize the data when comparing several population groups with different age classes or for the same population over time in which the age profiles change.

**Case definition**

The nasopharyngeal cancers are diagnosed by endoscopy, imaging studies, and biopsy in a patient with relevant clinical signs and symptoms.

The nasopharyngeal cancer coded as 147-147.9 in the 9th revision of the *International Classification of Disease and Injuries* (ICD-9) or C11-C11.9 in the ICD-10.

**Social demographic index**

Socio-demographic index (SDI) is a composite indicator that includes income per capita, average educational years and total fertility rate among individuals aged over 15 years. The calculation of SDI score in the GBD study was elaborated in the previous study[9].

According to Socio-demographic index (SDI) quintiles, 204 countries and territories were categorized into five groups: low-SDI, low-middle-SDI, middle-SDI, high-middle-SDI, and high-SDI quintiles[7].

We used the SDI to determine the relationship between the development level of a region or country and nasopharyngeal cancer YLD, YLL, DALY. It is the geometric mean of 0 to 1 indices of total fertility rate under the age of 25 (TFU25), mean education for those ages 15 and older (EDU15+), and lag distributed income (LDI) per capita. As a composite, a location with an SDI of 0 would have a theoretical minimum level of development relevant to health, while a location with an SDI of 1 would have a theoretical maximum level.

**Risk Factors**

The GBD 2019 used the comparative risk-assessment framework, used in GBD since 2002, to quantify associations between disease and risk factors[10]. Risk factors were divided into 3 categories: behavioral, environmental/occupational, and metabolic[11]. Among the 87 risk factor assess by GBD 2019, 3 major risk factors for nasopharyngeal cancer were confirmed: alcohol use, occupational exposure to formaldehyde, smoking[12].

**Uncertainty**

We captured and propagated uncertainty through all calculations by sampling 1000 values (called draws) for each prevalence, death, YLL, YLD, or DALY estimate and summing draws across age, cause, and location for all intermediate calculations. 95% uncertainty intervals (UIs) were defined by the ordinal 25th and 975th draw values.

**ARIMA model analysis**

The Autoregressive Integrated Moving Average (ARIMA) model is an investigative, data-oriented method, allowing the user to adjust the robustly oriented procedures that change over time by forecasting the scenarios future condition based on current situations[13, 14].We choose to use modest time-series methods such as the ARIMA model to forecast the ASR of the upcoming sixteen year until the end of 2035 in this research. To fit the model, we consider the model as ARIMA (p, d, q), where p represents the autoregressive term, d denotes the differencing order, and q indicates the moving averages term. Since the ARIMA model is the amalgamation of Autoregressive (AR) and Moving Average (MA) terms, we believe that it fits sound to the nature of the data and delivers good short-term forecasting. Parameters (p, d, q) are recognized by Autocorrelation function (ACF) and Partial Autocorrelation Function (PACF) for assessing the model fit. In addition, ARIMA (p,d,q) is chosen based on the Akaike information criterion (AIC), a goodness of fit test where the model with minimum AIC is considered here. We use R package tseries, and forecast to fit the ARIMA model. We run the ARIMA model through auto.arima function under the package forecast.

1. Collaborators, G.B.D.D., *Global age-sex-specific fertility, mortality, healthy life expectancy (HALE), and population estimates in 204 countries and territories, 1950-2019: a comprehensive demographic analysis for the Global Burden of Disease Study 2019.* Lancet, 2020. **396**(10258): p. 1160-1203.

2. Engholm, G., et al., *NORDCAN--a Nordic tool for cancer information, planning, quality control and research.* Acta Oncol, 2010. **49**(5): p. 725-36.

3. Foreman, K.J., et al., *Modeling causes of death: an integrated approach using CODEm.* Popul Health Metr, 2012. **10**: p. 1.

4. Disease, G.B.D., I. Injury, and C. Prevalence, *Global, regional, and national incidence, prevalence, and years lived with disability for 354 diseases and injuries for 195 countries and territories, 1990-2017: a systematic analysis for the Global Burden of Disease Study 2017.* Lancet, 2018. **392**(10159): p. 1789-1858.

5. Global Burden of Disease Cancer, C., et al., *Global, Regional, and National Cancer Incidence, Mortality, Years of Life Lost, Years Lived With Disability, and Disability-Adjusted Life-years for 32 Cancer Groups, 1990 to 2015: A Systematic Analysis for the Global Burden of Disease Study.* JAMA Oncol, 2017. **3**(4): p. 524-548.

6. Neal, R.D., et al., *Comparison of cancer diagnostic intervals before and after implementation of NICE guidelines: analysis of data from the UK General Practice Research Database.* Br J Cancer, 2014. **110**(3): p. 584-92.

7. Diseases, G.B.D. and C. Injuries, *Global burden of 369 diseases and injuries in 204 countries and territories, 1990-2019: a systematic analysis for the Global Burden of Disease Study 2019.* Lancet, 2020. **396**(10258): p. 1204-1222.

8. DALYs, G.B.D. and H. Collaborators, *Global, regional, and national disability-adjusted life-years (DALYs) for 359 diseases and injuries and healthy life expectancy (HALE) for 195 countries and territories, 1990-2017: a systematic analysis for the Global Burden of Disease Study 2017.* Lancet, 2018. **392**(10159): p. 1859-1922.

9. Alcohol, G.B.D. and C. Drug Use, *The global burden of disease attributable to alcohol and drug use in 195 countries and territories, 1990-2016: a systematic analysis for the Global Burden of Disease Study 2016.* Lancet Psychiatry, 2018. **5**(12): p. 987-1012.

10. Murray, C.J. and A.D. Lopez, *Global mortality, disability, and the contribution of risk factors: Global Burden of Disease Study.* Lancet, 1997. **349**(9063): p. 1436-42.

11. Murray, C.J., et al., *Comparative quantification of health risks conceptual framework and methodological issues.* Popul Health Metr, 2003. **1**(1): p. 1.

12. Collaborators, G.B.D.R.F., *Global burden of 87 risk factors in 204 countries and territories, 1990-2019: a systematic analysis for the Global Burden of Disease Study 2019.* Lancet, 2020. **396**(10258): p. 1223-1249.

13. Dyer, O., *Covid-19: Remdesivir has little or no impact on survival, WHO trial shows.* BMJ, 2020. **371**: p. m4057.

14. Papastefanopoulos, V., P. Linardatos, and S. Kotsiantis, *COVID-19: A Comparison of Time Series Methods to Forecast Percentage of Active Cases per Population.* Applied Sciences, 2020. **10**(11): p. 3880.
