# Supplementary material for: The global, regional, national burden of nasopharyngeal cancer and its attributable risk factors (1990–2019) and predictions to 2035
Source: Cancer Med. 2022 Apr 27;11(22):4310–20. doi: 10.1002/cam4.4783 (PMC9678109; doi:10.1002/cam4.4783)
Supplement: Supplementary file 3 — Table S1‐S12 [file CAM4-11-4310-s001.docx]

Supplementary Table 1 The incidence, death and DALYs for nasopharyngeal carcinoma in 2019 for both sexes, and percentage change of age-standardized rates (ASR) by GBD region, 1990-2019.

|  | Incidence (95%Uncertainty Interval) | | | Death (95%Uncertainty Interval) | | | DALYs (95%Uncertainty Interval) | | |
| --- | --- | --- | --- | --- | --- | --- | --- | --- | --- |
|  | Counts | ASR per 100,000 population (95%UI) | Percentage change in ASRs per 100,000 population (95%UI) (%) | Counts | ASR per 100,000 population (95%UI) | Percentage change in ASRs per 100,000 population(95%UI) (%) | Numbers | ASR per 100,000 population (95%UI) | Percentage change in ASRs per 100,000 population (95%UI) (%) |
| Global | 176502 (156046 to 199917) | 2.1 (1.9 to 2.4) | 37.1 (18.2 to 59.4) | 71610 (65442 to 77625) | 0.9 (0.8 to 0.9) | -31.3 (-38.9 to -22.5) | 2335096 (2139753 to 2536657) | 28 (25.7 to 30.4) | -32.9 (-40.6 to -23.8) |
| Andean Latin America | 102 (81 to 125) | 0.2 (0.1 to 0.2) | -4.3 (-24.8 to 20.2) | 89 (72 to 109) | 0.2 (0.1 to 0.2) | -14.5 (-32.3 to 6.6) | 2547 (2027 to 3164) | 4.3 (3.4 to 5.3) | -21.2 (-38.4 to 0.1) |
| Australasia | 305 (244 to 380) | 0.7 (0.6 to 0.9) | -21 (-38.6 to 0) | 133 (119 to 148) | 0.3 (0.3 to 0.3) | -36.3 (-42.7 to -28.9) | 3733 (3358 to 4127) | 9.4 (8.4 to 10.3) | -37 (-43.3 to -29.9) |
| Caribbean | 333 (282 to 386) | 0.6 (0.5 to 0.8) | 27.3 (8 to 48.5) | 269 (230 to 311) | 0.5 (0.4 to 0.6) | 11.7 (-4.3 to 28.6) | 7759 (6533 to 9118) | 15.2 (12.7 to 17.9) | 12.9 (-3.4 to 30.6) |
| Central Asia | 395 (350 to 447) | 0.5 (0.4 to 0.5) | 26.8 (11.2 to 45.1) | 336 (296 to 378) | 0.4 (0.4 to 0.5) | 19.3 (4.6 to 37.4) | 12211 (10777 to 13872) | 13.6 (12 to 15.4) | 18.2 (3.3 to 35.1) |
| Central Europe | 1034 (898 to 1189) | 0.6 (0.5 to 0.7) | 42 (22.8 to 63.6) | 738 (642 to 842) | 0.4 (0.3 to 0.5) | 4.9 (-9.8 to 19.6) | 21525 (18616 to 24716) | 12.8 (11.1 to 14.8) | 3.3 (-11.9 to 18.6) |
| Central Latin America | 563 (475 to 666) | 0.2 (0.2 to 0.3) | 2 (-14.4 to 20.3) | 448 (379 to 528) | 0.2 (0.2 to 0.2) | -15.1 (-28.1 to 0.5) | 13191 (11100 to 15759) | 5.3 (4.5 to 6.4) | -16 (-29.7 to 0.3) |
| Central Sub-Saharan Africa | 331 (253 to 421) | 0.5 (0.4 to 0.6) | -15.9 (-37.6 to 11.9) | 316 (242 to 403) | 0.5 (0.4 to 0.7) | -16.4 (-37.3 to 8.1) | 11213 (8559 to 14140) | 15.2 (11.7 to 19.4) | -17.3 (-38.7 to 10.1) |
| East Asia | 113395 (93188 to 135377) | 5.6 (4.7 to 6.7) | 67.4 (33.7 to 110.5) | 30103 (25229 to 35361) | 1.4 (1.2 to 1.7) | -50.3 (-59.8 to -38.4) | 958331 (810443 to 1124172) | 46.3 (39.3 to 54) | -49.9 (-59.3 to -38.1) |
| Eastern Europe | 1514 (1331 to 1697) | 0.5 (0.5 to 0.6) | 31.5 (15.6 to 49) | 963 (845 to 1091) | 0.3 (0.3 to 0.3) | -3.2 (-14.2 to 9.5) | 30888 (27091 to 35027) | 10.7 (9.4 to 12.1) | -1.9 (-12.8 to 11.6) |
| Eastern Sub-Saharan Africa | 2537 (1907 to 3110) | 1.2 (0.9 to 1.5) | -17.9 (-32.1 to 3.1) | 2409 (1813 to 2944) | 1.2 (0.9 to 1.5) | -17.3 (-31.3 to 4.2) | 90077 (67599 to 110875) | 38.4 (28.9 to 47) | -19.2 (-33.9 to 4.8) |
| High-income Asia Pacific | 5978 (5140 to 6935) | 1.7 (1.5 to 2) | 43.4 (22.7 to 68.5) | 1299 (1167 to 1410) | 0.3 (0.3 to 0.3) | -10.8 (-18 to -2.8) | 31876 (29131 to 34731) | 9.6 (8.8 to 10.5) | -17.5 (-24.6 to -9.3) |
| High-income North America | 3528 (3034 to 4101) | 0.7 (0.6 to 0.8) | -8.9 (-21.7 to 5.1) | 1266 (1208 to 1322) | 0.2 (0.2 to 0.2) | -33.7 (-36.5 to -30.6) | 36558 (34983 to 38168) | 7.1 (6.8 to 7.4) | -34 (-37 to -30.8) |
| North Africa and Middle East | 6642 (5776 to 7691) | 1.2 (1.1 to 1.4) | 16.4 (0.3 to 44.7) | 2950 (2572 to 3373) | 0.6 (0.5 to 0.7) | -33.2 (-42.3 to -21.1) | 102674 (89157 to 118580) | 19.1 (16.7 to 22) | -35.5 (-44.8 to -23.5) |
| Oceania | 109 (79 to 143) | 1.3 (0.9 to 1.6) | 0.5 (-20.7 to 28) | 103 (75 to 134) | 1.3 (0.9 to 1.6) | 0.8 (-20.3 to 27.7) | 3566 (2561 to 4741) | 38.2 (27.9 to 50) | -0.5 (-22.3 to 27.8) |
| South Asia | 15778 (13772 to 18199) | 1 (0.9 to 1.2) | -24.8 (-37.7 to -9.1) | 14665 (12957 to 16861) | 1 (0.8 to 1.1) | -27.1 (-39.4 to -10.2) | 508458 (449582 to 583996) | 31.1 (27.5 to 35.7) | -27.2 (-40 to -10.3) |
| Southeast Asia | 13120 (11396 to 15082) | 2 (1.7 to 2.2) | -9.6 (-22.7 to 6.9) | 11259 (9866 to 12847) | 1.7 (1.5 to 2) | -17.9 (-28.6 to -4.3) | 364166 (316978 to 418042) | 52.3 (45.7 to 60) | -20.6 (-31.7 to -7.2) |
| Southern Latin America | 228 (177 to 291) | 0.3 (0.2 to 0.4) | -24.1 (-42 to -1.9) | 150 (137 to 164) | 0.2 (0.2 to 0.2) | -0.5 (-0.4 to -0.5) | 4267 (3910 to 4661) | 5.5 (5.1 to 6) | -45.6 (-51 to -39.5) |
| Southern Sub-Saharan Africa | 306 (274 to 339) | 0.5 (0.4 to 0.6) | -13 (-21.9 to -3) | 291 (261 to 321) | 0.5 (0.4 to 0.5) | -0.1 (0.0 to -0.2) | 9266 (8256 to 10361) | 14.2 (12.7 to 15.8) | -16.7 (-25.4 to -7.5) |
| Tropical Latin America | 687 (644 to 731) | 0.3 (0.3 to 0.3) | 23.1 (12.8 to 33.1) | 537 (503 to 570) | 0.2 (0.2 to 0.2) | 0.0 (0.1 to 0.0) | 18585 (17416 to 19698) | 7.5 (7 to 7.9) | 4.3 (-4.5 to 13.4) |
| Western Europe | 8236 (7005 to 9635) | 1.2 (1 to 1.4) | 9.8 (-7.3 to 30.3) | 1990 (1866 to 2106) | 0.3 (0.2 to 0.3) | -0.4 (-0.4 to -0.5) | 56028 (52215 to 59854) | 8.4 (7.8 to 8.9) | -44.3 (-48 to -40.5) |
| Western Sub-Saharan Africa | 1382 (1092 to 1709) | 0.6 (0.5 to 0.7) | -4.7 (-27.8 to 25.8) | 1299 (1008 to 1590) | 0.6 (0.4 to 0.7) | -0.1 (0.2 to -0.3) | 48177 (36972 to 59608) | 18 (13.9 to 22.1) | -5.2 (-28 to 24.8) |

ASR, age-standardized rates; DALYs, disability adjusted life years.

Supplementary Table 2 The prevalence, YLLs and YLDs for NPC in 2019 for both sexes, and percentage change of age-standardized rates (ASRs) by Global Disease Burden region, 1990-2019.

|  | Prevalence (95%Uncertainty Interval) | | | YLLs (95%Uncertainty Interval) | | | YLDs (95%Uncertainty Interval) | | |
| --- | --- | --- | --- | --- | --- | --- | --- | --- | --- |
|  | Counts | ASR per 100,000 population (95%UI) | Percentage change in ASRs per 100,000 population (95%UI)(%) | Counts | ASR per 100,000 population (95%UI) | Percentage change in ASRs per 100,000 population(95%UI) (%) | Numbers | ASR per 100,000 population (95%UI) | Percentage change in ASRs per 100,000 population (95%UI) (%) |
| Global | 971935 (1119384 to 845408) | 11.7 (13.4 to 10.2) | 1.2 (1.5 to 0.8) | 4151 (4526 to 3812) | 26.8 (29.1 to 24.5) | -0.3 (-0.3 to -0.4) | 96734 (130872 to 68515) | 1.2 (1.6 to 0.8) | 0.7 (1.1 to 0.5) |
| Andean Latin America | 335 (419 to 264) | 0.6 (5.2 to 3.3) | 0.1 (0.5 to -0.1) | 2238361 (2428099 to 2045463) | 4.2 (5.2 to 3.4) | -0.2 (0.0 to -0.4) | 44 (61 to 30) | 0.1 (0.1 to 0.1) | 0.0 (0.3 to -0.2) |
| Australasia | 1625 (2028 to 1298) | 4.1 (2.7 to 1.9) | -0.1 (0.1 to -0.3) | 3561 (3929 to 3201) | 9.0 (9.9 to 8.1) | -0.4 (-0.3 to -0.4) | 172 (245 to 112) | 0.4 (0.6 to 0.3) | -0.2 (0.1 to -0.4) |
| Caribbean | 1175 (1399 to 987) | 2.3 (1.7 to 1.3) | 0.5 (0.8 to 0.2) | 2503 (3116 to 1992) | 14.9 (17.5 to 12.5) | 0.1 (0.3 to 0.0) | 152 (210 to 104) | 0.3 (0.4 to 0.2) | 0.4 (0.6 to 0.2) |
| Central Asia | 1318 (1502 to 1157) | 1.5 (3.2 to 2.3) | 0.4 (0.6 to 0.2) | 12935 (15445 to 10866) | 13.4 (15.2 to 11.9) | 0.2 (0.3 to 0.0) | 165 (226 to 116) | 0.2 (0.3 to 0.1) | 0.3 (0.5 to 0.1) |
| Central Europe | 4142 (4838 to 3563) | 2.7 (1.0 to 0.7) | 0.9 (1.3 to 0.6) | 892386 (1058872 to 744055) | 12.5 (14.4 to 10.8) | 0.0 (0.2 to -0.1) | 489 (667 to 344) | 0.3 (0.4 to 0.2) | 0.6 (0.9 to 0.4) |
| Central Latin America | 2058 (2449 to 1726) | 0.8 (1.7 to 1.0) | 0.3 (0.6 to 0.1) | 27986 (30231 to 25779) | 5.2 (6.2 to 4.4) | -0.2 (0.0 to -0.3) | 256 (351 to 175) | 0.1 (0.1 to 0.1) | 0.1 (0.3 to 0.0) |
| Central Sub-Saharan Africa | 932 (1177 to 712) | 1.3 (41.8 to 28.9) | -0.1 (0.2 to -0.4) | 9143 (10232 to 8160) | 15.0 (19.2 to 11.5) | -0.2 (0.1 to -0.4) | 131 (193 to 85) | 0.2 (0.3 to 0.1) | -0.2 (0.1 to -0.4) |
| East Asia | 703523 (845831 to 579101) | 35.0 (2.7 to 2.1) | 2.2 (3.1 to 1.5) | 99192 (114562 to 86104) | 43.0 (50.9 to 36.1) | -0.5 (-0.4 to -0.6) | 65945 (91101 to 44958) | 3.3 (4.5 to 2.2) | 1.4 (2.0 to 0.9) |
| Eastern Europe | 6508 (7359 to 5654) | 2.4 (4.0 to 2.5) | 0.7 (0.9 to 0.4) | 358672 (411763 to 312271) | 10.4 (11.8 to 9.1) | 0.0 (0.1 to -0.1) | 737 (998 to 512) | 0.3 (0.4 to 0.2) | 0.5 (0.7 to 0.3) |
| Eastern Sub-Saharan Africa | 7431 (9140 to 5580) | 3.3 (13.0 to 9.8) | -0.1 (0.1 to -0.3) | 12045 (13643 to 10635) | 38.0 (46.4 to 28.6) | -0.2 (0.0 to -0.3) | 999 (1430 to 643) | 0.5 (0.7 to 0.3) | -0.2 (0.0 to -0.3) |
| High-income Asia Pacific | 37319 (43216 to 32140) | 11.3 (4.7 to 3.5) | 0.6 (0.9 to 0.3) | 89078 (109736 to 66987) | 8.5 (9.2 to 7.9) | -0.2 (-0.1 to -0.3) | 3890 (5320 to 2745) | 1.1 (1.5 to 0.8) | 0.5 (0.8 to 0.3) |
| High-income North America | 20049 (23291 to 17251) | 4.1 (7.4 to 5.5) | 0.0 (0.2 to -0.1) | 7606 (8955 to 6397) | 6.7 (6.9 to 6.4) | -0.4 (-0.3 to -0.4) | 2098 (2821 to 1448) | 0.4 (0.6 to 0.3) | 0.0 (0.1 to -0.2) |
| North Africa and Middle East | 35392 (41859 to 30438) | 6.3 (4.3 to 2.5) | 0.8 (1.3 to 0.5) | 21035 (24100 to 18176) | 18.5 (21.2 to 16.1) | -0.4 (-0.2 to -0.5) | 3481 (4683 to 2428) | 0.7 (0.9 to 0.5) | 0.4 (0.8 to 0.2) |
| Oceania | 304 (400 to 222) | 3.3 (3.3 to 2.5) | 0.0 (0.3 to -0.2) | 524032 (599822 to 452991) | 37.7 (49.3 to 27.6) | 0.0 (0.3 to -0.2) | 43 (63 to 27) | 0.5 (0.7 to 0.3) | 0.0 (0.3 to -0.2) |
| South Asia | 46405 (53325 to 40491) | 2.9 (7.2 to 5.4) | -0.2 (0.0 to -0.3) | 11082 (13971 to 8481) | 30.7 (35.3 to 27.1) | -0.3 (-0.1 to -0.4) | 6290 (8419 to 4536) | 0.4 (0.5 to 0.3) | -0.2 (-0.1 to -0.4) |
| Southeast Asia | 43659 (50487 to 37311) | 6.2 (1.6 to 1.0) | 0.1 (0.3 to -0.1) | 50774 (53710 to 47925) | 51.5 (58.9 to 44.9) | -0.2 (-0.1 to -0.3) | 5494 (7419 to 3876) | 0.8 (1.1 to 0.6) | 0.0 (0.1 to -0.2) |
| Southern Latin America | 958 (1243 to 734) | 1.3 (1.5 to 1.2) | 0.0 (0.4 to -0.2) | 47631 (59012 to 36619) | 5.4 (5.9 to 5.0) | -0.5 (-0.4 to -0.5) | 115 (169 to 74) | 0.1 (0.2 to 0.1) | -0.1 (0.2 to -0.3) |
| Southern Sub-Saharan Africa | 868 (966 to 775) | 1.4 (1.1 to 1.0) | -0.1 (0.0 to -0.2) | 199420 (224866 to 175945) | 14.0 (15.5 to 12.5) | -0.2 (-0.1 to -0.3) | 123 (167 to 86) | 0.2 (0.3 to 0.1) | -0.1 (0.0 to -0.2) |
| Tropical Latin America | 2535 (2724 to 2363) | 1.0 (9.2 to 6.6) | 0.5 (0.7 to 0.4) | 147596 (159957 to 136621) | 7.3 (7.8 to 6.9) | 0.0 (0.1 to 0.0) | 304 (402 to 220) | 0.1 (0.2 to 0.1) | 0.4 (0.5 to 0.2) |
| Western Europe | 51363 (60459 to 43569) | 7.8 (1.9 to 1.2) | 0.3 (0.6 to 0.1) | 487557 (543797 to 440204) | 7.6 (8.0 to 7.2) | -0.5 (-0.4 to -0.5) | 5253 (7164 to 3616) | 0.8 (1.0 to 0.5) | 0.2 (0.5 to 0.0) |
| Western Sub-Saharan Africa | 4026 (5010 to 3155) | 1.6 (13.4 to 10.2) | 0.0 (0.3 to -0.3) | 878644 (980665 to 784149) | 17.8 (21.8 to 13.8) | -0.1 (0.2 to -0.3) | 545 (784 to 357) | 0.2 (0.3 to 0.1) | 0.0 (0.3 to -0.3) |

SDI, Socio-demographic Index; YLDs, years lived with disability; YLLs, years of life lost.

Supplementary Table 3 The incidence, deaths and DALYs for NPC in 2019 for both sexes, and percentage change of age-standardized rates (ASRs) by SDI quintile, 1990-2019.

|  | Incidence (95%Uncertainty Interval) | | | Death (95%Uncertainty Interval) | | | DALYs (95%Uncertainty Interval) | | |
| --- | --- | --- | --- | --- | --- | --- | --- | --- | --- |
|  | Counts | ASR per 100,000 population (95%UI) | Percentage change in ASRs per 100,000 population (95%UI) | Counts | ASR per 100,000 population (95%UI) | Percentage change in ASRs per 100,000 population(95%UI) | Numbers | ASR per 100,000 population (95%UI) | Percentage change in ASRs per 100,000 population (95%UI) |
| High SDI | 19265 (17281 to 21480) | 1.3 (1.2 to 1.4) | 12.9 (0.8 to 25.8) | 5552 (5139 to 5977) | 0.3 (0.3 to 0.4) | -34.7 (-39.1 to -29.7) | 159393 (146905 to 173317) | 10.8 (10 to 11.8) | -36.5 (-41.3 to -31.1) |
| High-middle SDI | 68017 (56467 to 81567) | 3.6 (3 to 4.3) | 111.3 (70.6 to 168.6) | 17221 (14917 to 19678) | 0.9 (0.8 to 1) | -34.2 (-44.6 to -21.6) | 563898 (492008 to 643059) | 29.5 (25.8 to 33.6) | -32.8 (-44.3 to -19.9) |
| Middle SDI | 57527 (50144 to 65912) | 2.2 (1.9 to 2.5) | 6.9 (-9.9 to 27.3) | 28750 (25630 to 32051) | 1.1 (1 to 1.2) | -40.3 (-48.5 to -30) | 914423 (819013 to 1020649) | 34 (30.5 to 37.9) | -42.6 (-50.7 to -32.8) |
| Low-middle SDI | 16590 (15068 to 18482) | 1.1 (1 to 1.2) | -19 (-30 to -6.6) | 14509 (13154 to 16111) | 1 (0.9 to 1.1) | -25.3 (-34.9 to -13.6) | 494502 (446245 to 550735) | 31.4 (28.4 to 35) | -26.7 (-36.6 to -14.9) |
| Low SDI | 5383 (4773 to 5974) | 0.8 (0.8 to 0.9) | -26.5 (-38.2 to -12.7) | 5544 (4904 to 6231) | 0.9 (0.8 to 1.0) | -20.1 (-33 to -4.3) | 201746 (177985 to 227158) | 29 (25.5 to 32.6) | -22.2 (-35.6 to -4.8) |

Abbreviations: DALYs, disability adjusted life years; SDI, Socio-demographic Index.

Supplementary Table 4 Prevalence, YLLs and YLDs for NPC in 2019 for both sexes, and percentage change of age-standardized rates (ASRs) by SDI quintile, 1990-2019.

|  | Prevalence (95%Uncertainty Interval) | | | YLLs (95%Uncertainty Interval) | | | YLDs (95%Uncertainty Interval) | | |
| --- | --- | --- | --- | --- | --- | --- | --- | --- | --- |
|  | Counts | ASR per 100,000 population (95%UI) | Percentage change in ASRs per 100,000 population (95%UI) | Numbers | ASR per 100,000 population (95%UI) | Percentage change in ASRs per 100,000 population(95%UI) | Numbers | ASR per 100,000 population (95%UI) | Percentage change in ASRs per 100,000 population (95%UI) |
| High SDI | 159393 (146905 to 173317) | 8 (7.2 to 9) | 33.3 (18.4 to 49.3) | 147596 (136621 to 159957) | 10 (9.3 to 10.9) | -38.9 (-43.4 to -33.7) | 11797 (8361 to 15650) | 0.8 (0.6 to 1) | 33.3 (18.4 to 49.3) |
| High-middle SDI | 563898 (492008 to 643059) | 22.7 (18.7 to 27.3) | 265.1 (191.4 to 367.1) | 524032 (452991 to 599822) | 27.4 (23.8 to 31.3) | -36.5 (-47.3 to -23.8) | 39866 (27297 to 55805) | 2.1 (1.4 to 2.9) | 265.1 (191.4 to 367.1) |
| Middle SDI | 914423 (819013 to 1020649) | 13.2 (11.3 to 15.4) | 118.9 (79.5 to 166.8) | 878644 (784149 to 980665) | 32.7 (29.2 to 36.4) | -44.1 (-52 to -34.3) | 35778 (25003 to 48688) | 1.3 (0.9 to 1.8) | 118.9 (79.5, to 166.8) |
| Low-middle SDI | 494502 (446245 to 550735) | 3.4 (3.1 to 3.8) | -6.1 (-19.3 to 9.9) | 487557 (440204 to 543797) | 31 (28 to 34.5) | -26.8 (-36.8 to -15) | 6945 (4947 to 9222) | 0.5 (0.3 to 0.6) | -6.1 (-19.3 to 9.9) |
| Low SDI | 201746 (177985 to 227158) | 2.5 (2.2 to 2.8) | -16.7 (-30.9 to 0.2) | 199420 (175945 to 224866) | 28.6 (25.2 to 32.2) | -22.2 (-35.7 to -4.8) | 2326 (1629 to 3169) | 0.4 (0.3 to 0.5) | -16.7 (-30.9 to 0.2) |

SDI, Socio-demographic Index; YLDs, years lived with disability; YLLs, years of life lost.

Supplementary Table 5 The incidence, death and DALYS for NPC for both sexes by Global Disease Burden region in 1990.

|  | Incidence (95%Uncertainty Interval) | | Death (95%Uncertainty Interval) | | DALYs (95%Uncertainty Interval) | |
| --- | --- | --- | --- | --- | --- | --- |
|  | Counts | ASR per 100,000 population (95%UI) | Counts | ASR per 100,000 population (95%UI) | Numbers | ASR per 100,000 population (95%UI) |
| Global | 67518 (61729 to 72995) | 1.5 (1.4 to 1.7） | 53459 (48875 to 57906) | 1.3 (1.2 to 1.4) | 1880702 (1715976 to 2050611) | 41.7 (38.1 to 45.4) |
| High SDI | 10903 (10624 to 11237) | 1.1 (1.1 to 1.2) | 5021 (4908 to 5122) | 0.5 (0.5 to 0.5) | 161255 (157625 to 164721) | 17.0 (16.7 to 17.4) |
| High-middle SDI | 18963 (16645 to 21359) | 1.7 (1.5 to 1.9) | 14536 (12863 to 16381) | 1.3 (1.2 to 1.5) | 497596 (437255 to 564801) | 43.9 (38.6 to 49.8) |
| Middle SDI | 24544 (21723 to 27165) | 2.0 (1.8 to 2.2) | 21554 (19334 to 23733) | 1.9 (1.7 to 2.1) | 769908 (692954 to 849323) | 59.3 (53.3 to 65.4) |
| Low-middle SDI | 9719 (8476 to 11224) | 1.3 (1.2 to 1.6) | 9128 (8021 to 10478) | 1.3 (1.2 to 1.5) | 333544 (291403 to 386816) | 42.8 (37.6 to 49.4) |
| Low SDI | 3366 (2815 to 4072) | 1.2 (1.0 to 1.4) | 3201 (2685 to 3851) | 1.1 (1.0 to 1.4) | 117760 (97503 to 143749) | 37.2 (31.2 to 45.0) |
| Andean Latin America | 43 (39 to 48) | 0.2 (0.2 to 0.2) | 41 (37 to 46) | 0.2 (0.2 to 0.2) | 1420 (1272 to 1594) | 5.5 (4.9 to 6.1) |
| Australasia | 212 (193 to 232) | 0.9 (0.9 to 1.0) | 107 (102 to 113) | 0.5 (0.4 to 0.5) | 3309 (3127 to 3492) | 14.9 (14.1 to 15.7) |
| Caribbean | 139 (127 to 150) | 0.5 (0.5 to 0.5) | 123 (113 to 133) | 0.5 (0.4 to 0.5) | 3840 (3403 to 4219) | 13.4 (12.0 to 14.7) |
| Central Asia | 194 (178 to 218) | 0.4 (0.3 to 0.4) | 176 (161 to 200) | 0.3 (0.3 to 0.4) | 6478 (5983 to 7190) | 11.5 (10.6 to 12.9) |
| Central Europe | 622 (602 to 642) | 0.4 (0.4 to 0.5) | 543 (528 to 560) | 0.4 (0.4 to 0.4) | 17575 (17019 to 18107) | 12.4 (12.0 to 12.8) |
| Central Latin America | 220 (213 to 226) | 0.2 (0.2 to 0.2) | 200 (194 to 206) | 0.2 (0.2 to 0.2) | 6830 (6641 to 7019) | 6.3 (6.2 to 6.5) |
| Central Sub-Saharan Africa | 165 (132 to 205) | 0.6 (0.5 to 0.7) | 159 (130 to 192) | 0.6 (0.5 to 0.7) | 5613 (4504 to 6945) | 18.4 (15.0 to 22.4) |
| East Asia | 33792 (29132 to 38286) | 3.4 (2.9 to 3.8) | 27761 (23907 to 31520) | 2.9 (2.5 to 3.3) | 978436 (840861 to 1115057) | 92.4 (79.5 to 104.9) |
| Eastern Europe | 1076 (998 to 1180) | 0.4 (0.4 to 0.4) | 868 (810 to 944) | 0.3 (0.3 to 0.3) | 28845 (26851 to 31426) | 10.9 (10.2 to 11.9) |
| Eastern Sub-Saharan Africa | 1388 (1125 to 1681) | 1.5 (1.2 to 1.8) | 1315 (1059 to 1597) | 1.5 (1.2 to 1.7) | 49134 (39213 to 60688) | 47.5 (38.3 to 58.1) |
| High-income Asia Pacific | 2462 (2286 to 2626) | 1.2 (1.1 to 1.3) | 726 (697 to 751) | 0.4 (0.3 to 0.4) | 23898 (22859 to 24950) | 11.7 (11.2 to 12.2) |
| High-income North America | 2394 (2319 to 2475) | 0.8 (0.7 to 0.8) | 1090 (1060 to 1119) | 0.3 (0.3 to 0.3) | 33228 (32397 to 34081) | 10.7 (10.4 to 11.0) |
| North Africa and Middle East | 2298 (1979 to 2601) | 1.1 (0.9 to 1.2) | 1823 (1561 to 2075) | 0.9 (0.8 to 1.0) | 67441 (57954 to 77327) | 29.7 (25.4 to 33.9) |
| Oceania | 46 (35 to 57) | 1.3 (1.0 to 1.6) | 43 (33 to 54) | 1.2 (1.0 to 1.5) | 1502 (1145 to 1880) | 38.4 (29.6 to 47.9) |
| South Asia | 9347 (8093 to 10931) | 1.3 (1.2 to 1.6) | 8841 (7644 to 10310) | 1.3 (1.1 to 1.5) | 325298 (279995 to 381204) | 42.7 (36.9 to 49.8) |
| Southeast Asia | 6494 (5685 to 7152) | 2.2 (1.9 to 2.4) | 6084 (5354 to 6667) | 2.1 (1.9 to 2.3) | 214767 (187732 to 236156) | 65.8 (58.0 to 72.3) |
| Southern Latin America | 178 (167 to 189) | 0.4 (0.4 to 0.4) | 155 (146 to 165) | 0.3 (0.3 to 0.4) | 4790 (4514 to 5071) | 10.2 (9.6 to 10.8) |
| Southern Sub-Saharan Africa | 180 (164 to 198) | 0.6 (0.5 to 0.6) | 170 (154 to 188) | 0.6 (0.5 to 0.6) | 5801 (5306 to 6344) | 17.0 (15.5 to 18.7) |
| Tropical Latin America | 251 (241 to 263) | 0.2 (0.2 to 0.2) | 222 (212 to 232) | 0.2 (0.2 to 0.2) | 8518 (8136 to 8902) | 7.2 (6.8 to 7.5) |
| Western Europe | 5398 (5176 to 5630) | 1.1 (1.1 to 1.2) | 2412 (2340 to 2481) | 0.5 (0.4 to 0.5) | 72967 (70780 to 75188) | 15.0 (14.6 to 15.4) |
| Western Sub-Saharan Africa | 620 (494 to 774) | 0.6 (0.5 to 0.7) | 598 (476 to 748) | 0.6 (0.5 to 0.7) | 21012 (16704 to 26583) | 19.0 (15.1 to 24.0 |

Supplementary Table 6 The prevalence, YLLs and YLDs for NPC for both sexes by Global Disease Burden region in 1990.

|  | Prevalence (95%Uncertainty Interval) | | YLLs (95%Uncertainty Interval) | | YLDs (95%Uncertainty Interval) | |
| --- | --- | --- | --- | --- | --- | --- |
|  | Counts | ASR per 100,000 population (95%UI) | Counts | ASR per 100,000 population (95%UI) | Numbers | ASR per 100,000 population (95%UI) |
| Global | 243481 (223234 to 262233) | 5.4 (5.0 to 5.8) | 1851376 (1688182 to 2021511) | 41.0 (37.4 (44.7 | 29326 (21065 to 38799) | 0.7 (0.5 to 0.9) |
| High SDI | 56632 (54713 to 58876) | 6.0 (5.8 to 6.3) | 155272 (152106 to 158298) | 16.4 (16.1 to 16.7) | 5983 (4310 to 7890) | 0.6 (0.5 to 0.8) |
| High-middle SDI | 70886 (61922 to 80799) | 6.2 (5.4 to 7.1) | 489332 (430004 to 556691) | 43.1 (37.9 to 49.1) | 8264 (5883 to 11157) | 0.7 (0.5 to 1.0) |
| Middle SDI | 78584 (69354 to 86998) | 6.0 (5.3 to 6.7) | 759966 (683209 to 840312) | 58.5 (52.6 to 64.6) | 9941 (7099 to 13192) | 0.8 (0.6 to 1.1) |
| Low-middle SDI | 27914 (24231 to 32424) | 3.7 (3.2 to 4.2) | 329731 (288046 to 382389) | 42.3 (37.0 to 48.7) | 3813 (2650 to 5190) | 0.5 (0.4 to 0.7) |
| Low SDI | 9386 (7841 to 11407) | 3.0 (2.6 to 3.7) | 116445 (96394 to 142133) | 36.8 (30.8 to 44.4) | 1314(902 to 1830) | 0.5 (0.3 to 0.6) |
| Andean Latin America | 125 (111 to 140) | 0.5 (0.4 to 0.6) | 1403 (1257 to 1573) | 5.4 (4.8 to 6.0) | 17 (12 to 23) | 0.1 (0.1 to 0.1) |
| Australasia | 1059 (930 to 1195) | 4.8 (4.2 to 5.4) | 3195 (3022 to 3371) | 14.4 (13.6 to 15.2) | 113 (79 to 150) | 0.5 (0.4 to 0.7) |
| Caribbean | 441 (402 to 482) | 1.5 (1.4 to 1.7) | 3781 (3345 to 4147) | 13.2 (11.8 to 14.4) | 59 (42 to 77) | 0.2 (0.2 to 0.3) |
| Central Asia | 597 (549 to 666) | 1.1 (1.0 to 1.2) | 6399 (5908 to 7102) | 11.4 (10.5 to 12.7) | 79 (55 to 107) | 0.1 (0.1 to 0.2) |
| Central Europe | 1987 (1907 to 2069) | 1.4 (1.4 to 1.5 | 17313 (16784 to 17847) | 12.2 (11.9 to 12.6) | 262 (188 to 343) | 0.2 (0.1 to 0.2) |
| Central Latin America | 679 (658 to 703) | 0.6 (0.6 to 0.7) | 6739 (6555 to 6927) | 6.3 (6.1 to 6.4) | 90 (64 to 117) | 0.1 (0.1 to 0.1) |
| Central Sub-Saharan Africa | 453 (360 to 568) | 1.5 (1.2 to 1.9) | 5547 (4448 to 6858) | 18.2 (14.8 to 22.0) | 65 (44 to 91) | 0.2 (0.2 to 0.3) |
| East Asia | 117458 (101033 to 133789) | 10.9 (9.4 to 12.4) | 964353 (827536 to 1100281) | 91.0 (78.2 to 103.4) | 14084 (10055 to 18933) | 1.4 (1.0 to 1.9) |
| Eastern Europe | 3755 (3433 to 4193) | 1.4 (1.3 to 1.6) | 28372 (26433 to 31007) | 10.7 (10.0 to 11.7) | 473 (335 to 634) | 0.2 (0.1 to 0.2) |
| Eastern Sub-Saharan Africa | 3863 (3131 to 4708) | 3.8 (3.1 to 4.6) | 48591 (38753 to 59973) | 47.0 (37.8 to 57.3) | 543 (370 to 762) | 0.6 (0.4 to 0.8) |
| High-income Asia Pacific | 14763 (13500 to 15942) | 7.2 (6.6 to 7.7) | 22404 (21447 to 23259) | 11.0 (10.5 to 11.4) | 1494 (1068 to 1998) | 0.7 (0.5 to 1.0) |
| High-income North America | 12449 (11979 to 12964) | 4.0 (3.9 to 4.2) | 31901 (31186 to 32643) | 10.3 (10.1 to 10.5) | 1327 (945 to 1746) | 0.4 (0.3 to 0.6) |
| North Africa and Middle East | 8360 (7193 to 9468) | 3.6 (3.1 to 4.0) | 66459 (56963 to 76213) | 29.2 (25.0 to 33.3) | 982 (686 to 1303) | 0.5 (0.3 to 0.6) |
| Oceania | 126 (97 to 156) | 3.3 (2.6 to 4.1) | 1484 (1132 to 1859) | 37.9 (29.3 to 47.3) | 18 (12 to 26) | 0.5 (0.3 to 0.7) |
| South Asia | 26555 (22863 to 31032) | 3.6 (3.1 to 4.2) | 321652 (277233 to 377554) | 42.2 (36.5 to 49.3) | 3646 (2564 to 4922) | 0.5 (0.4 to 0.7) |
| Southeast Asia | 18984 (16559 to 20958) | 5.9 (5.2 to 6.5) | 212214 (185606 to 233406) | 65.0 (57.2 to 71.3) | 2553 (1809 to 3345) | 0.8 (0.6 to 1.1) |
| Southern Latin America | 566 (529 to 606) | 1.2 (1.1 to 1.3) | 4714 (4448 to 4992) | 10.0 (9.4 to 10.6) | 76 (55 to 100) | 0.2 (0.1 to 0.2) |
| Southern Sub-Saharan Africa | 513 (470 to 565) | 1.5 (1.4 to 1.7) | 5729 (5236 to 6268) | 16.8 (15.3 to 18.5) | 72 (51 to 97) | 0.2 (0.2 to 0.3) |
| Tropical Latin America | 795 (760 to 834) | 0.7 (0.6 to 0.7) | 8416 (8028 to 8800) | 7.1 (6.7 to 7.4) | 102 (73 to 135) | 0.1 (0.1 to 0.1) |
| Western Europe | 28228 (26757 to 29901) | 6.0 (5.6 to 6.3) | 69940 (68067 to 71890) | 14.4 (14.0 to 14.8) | 3027 (2169 to 3980) | 0.6 (0.4 to 0.8) |
| Western Sub-Saharan Africa | 1724 (1367 to 2161) | 1.6 (1.3 to 2.0) | 20769 (16488 to 26259) | 18.8 (14.9 to 23.7) | 243 (159 to 338) | 0.2 (0.2 to 0.3) |

DALYs, disability adjusted life years.

Supplementary Table 7 The incidence, death and DALYs for NPC for both sex in 2019, and percentage change of age-standardized rates (ASRs) by 204 countries, 1990-2019.

|  | Incidence (95% Uncertainty Interval) | | | Death (95% Uncertainty Interval) | | | DALYs (95% Uncertainty Interval) | | |
| --- | --- | --- | --- | --- | --- | --- | --- | --- | --- |
|  | Counts | ASR per 100,000 population (95%UI) | Percentage change in ASRs per 100,000 population (95%UI) | Counts | ASR per 100,000 population (95%UI) | Percentage change in ASRs per 100,000 population(95%UI) | Numbers | ASR per 100,000 population (95%UI) | Percentage change in ASRs per 100,000 population (95%UI) |
| Afghanistan | 112 (67, 164) | 0.6 (0.4, 0.9) | -0.3 (-0.5, 0) | 97 (59, 143) | 0.6 (0.4, 0.8) | -0.3 (-0.5, -0.1) | 3870 (2319, 5830) | 18.6 (11.7, 27.1) | -0.3 (-0.5, -0.1) |
| Albania | 17 (13, 22) | 0.5 (0.3, 0.6) | -0.2 (-0.4, 0) | 14 (11, 18) | 0.4 (0.3, 0.5) | -0.4 (-0.6, -0.2) | 341 (254, 449) | 9.2 (6.9, 12.2) | -0.4 (-0.6, -0.2) |
| Algeria | 1687 (1272, 2210) | 4.2 (3.2, 5.4) | 0.1 (-0.2, 0.5) | 659 (514, 830) | 1.8 (1.4, 2.3) | -0.4 (-0.6, -0.2) | 23276 (17981, 29412) | 58 (45, 73.2) | -0.5 (-0.6, -0.2) |
| American Samoa | 1 (1, 1) | 1.7 (1.4, 2) | 0.1 (-0.1, 0.5) | 1 (1, 1) | 1.6 (1.3, 1.9) | 0.1 (-0.1, 0.4) | 25 (20, 31) | 47.1 (38.1, 58.1) | 0.1 (-0.1, 0.5) |
| Andorra | 2 (1, 3) | 1.4 (1, 2) | 0.2 (-0.2, 0.8) | 0 (0, 1) | 0.3 (0.2, 0.4) | -0.3 (-0.6, 0) | 12 (9, 17) | 9.4 (7, 12.6) | -0.3 (-0.5, 0) |
| Angola | 76 (55, 98) | 0.5 (0.4, 0.7) | -0.1 (-0.4, 0.3) | 72 (52, 93) | 0.5 (0.4, 0.7) | -0.1 (-0.4, 0.2) | 2592 (1838, 3345) | 16.2 (11.7, 20.9) | -0.2 (-0.4, 0.2) |
| Antigua and Barbuda | 0 (0, 1) | 0.5 (0.4, 0.5) | 0.3 (0.1, 0.6) | 0 (0, 0) | 0.4 (0.3, 0.5) | 0.2 (0, 0.5) | 11 (9, 13) | 10.2 (8.4, 12.3) | 0.1 (-0.1, 0.4) |
| Argentina | 149 (113, 189) | 0.3 (0.2, 0.4) | -0.3 (-0.5, -0.1) | 107 (96, 119) | 0.2 (0.2, 0.2) | -0.5 (-0.5, -0.4) | 3063 (2765, 3407) | 6.1 (5.5, 6.8) | -0.5 (-0.5, -0.4) |
| Armenia | 10 (9, 13) | 0.3 (0.2, 0.3) | 0 (-0.2, 0.3) | 8 (7, 10) | 0.2 (0.2, 0.3) | -0.1 (-0.3, 0.1) | 277 (231, 331) | 7.7 (6.4, 9.1) | -0.2 (-0.3, 0) |
| Australia | 262 (202, 337) | 0.8 (0.6, 1) | -0.2 (-0.4, 0) | 113 (100, 127) | 0.3 (0.3, 0.3) | -0.4 (-0.4, -0.3) | 3156 (2801, 3528) | 9.4 (8.3, 10.5) | -0.4 (-0.5, -0.3) |
| Austria | 86 (65, 112) | 0.6 (0.5, 0.8) | -0.1 (-0.3, 0.2) | 29 (25, 32) | 0.2 (0.2, 0.2) | -0.5 (-0.6, -0.4) | 786 (690, 893) | 5.6 (5, 6.4) | -0.5 (-0.6, -0.4) |
| Azerbaijan | 21 (17, 26) | 0.2 (0.2, 0.2) | 0.4 (0.1, 0.7) | 18 (15, 22) | 0.2 (0.1, 0.2) | 0.3 (0, 0.6) | 677 (544, 837) | 6.2 (5, 7.5) | 0.2 (-0.1, 0.6) |
| Bahamas | 3 (2, 3) | 0.6 (0.5, 0.8) | 0.1 (-0.1, 0.4) | 2 (2, 3) | 0.5 (0.4, 0.7) | 0.1 (-0.2, 0.3) | 68 (54, 85) | 15.8 (12.6, 19.9) | 0.1 (-0.2, 0.3) |
| Bahrain | 9 (7, 12) | 0.6 (0.5, 0.8) | -0.2 (-0.4, 0.2) | 4 (3, 5) | 0.3 (0.2, 0.4) | -0.5 (-0.7, -0.4) | 137 (102, 181) | 9.1 (6.9, 11.8) | -0.5 (-0.7, -0.3) |
| Bangladesh | 1304 (922, 1828) | 0.9 (0.7, 1.3) | -0.4 (-0.6, -0.1) | 1210 (862, 1703) | 0.9 (0.6, 1.2) | -0.4 (-0.6, -0.1) | 40920 (29017, 58151) | 27.9 (19.7, 39.5) | -0.4 (-0.6, -0.1) |
| Barbados | 3 (3, 4) | 0.8 (0.6, 0.9) | 0.5 (0.2, 0.8) | 3 (2, 4) | 0.6 (0.5, 0.7) | 0.3 (0.1, 0.6) | 73 (59, 91) | 16.6 (13.4, 20.6) | 0.3 (0, 0.6) |
| Belarus | 69 (50, 93) | 0.5 (0.4, 0.7) | 0.3 (-0.1, 0.8) | 48 (35, 65) | 0.3 (0.2, 0.4) | 0 (-0.3, 0.3) | 1508 (1084, 2026) | 10.8 (7.8, 14.5) | 0 (-0.3, 0.3) |
| Belgium | 217 (163, 287) | 1.2 (0.9, 1.7) | 0.3 (0, 0.9) | 54 (48, 61) | 0.3 (0.2, 0.3) | -0.3 (-0.4, -0.2) | 1535 (1358, 1752) | 8.9 (7.8, 10.1) | -0.3 (-0.4, -0.2) |
| Belize | 1 (1, 1) | 0.4 (0.3, 0.5) | 0.7 (0.4, 1.1) | 1 (1, 1) | 0.4 (0.3, 0.4) | 0.6 (0.3, 0.9) | 36 (31, 43) | 10.7 (9.1, 12.6) | 0.7 (0.4, 1) |
| Benin | 19 (13, 26) | 0.3 (0.2, 0.4) | 0.2 (-0.1, 0.7) | 17 (12, 24) | 0.3 (0.2, 0.4) | 0.2 (-0.1, 0.6) | 666 (456, 939) | 9.4 (6.5, 13.2) | 0.2 (-0.2, 0.7) |
| Bermuda | 1 (1, 1) | 0.9 (0.8, 1.2) | 0.4 (0.1, 0.8) | 1 (0, 1) | 0.5 (0.4, 0.6) | -0.2 (-0.4, 0) | 13 (11, 17) | 12 (9.6, 14.8) | -0.2 (-0.4, 0) |
| Bhutan | 6 (4, 8) | 0.9 (0.6, 1.2) | -0.3 (-0.5, 0.1) | 5 (4, 7) | 0.9 (0.6, 1.2) | -0.3 (-0.5, 0) | 170 (112, 240) | 26.1 (17.4, 36.4) | -0.4 (-0.6, 0) |
| Bolivia (Plurinational State of) | 23 (17, 30) | 0.3 (0.2, 0.3) | -0.1 (-0.3, 0.3) | 22 (17, 29) | 0.3 (0.2, 0.3) | -0.1 (-0.3, 0.3) | 658 (490, 861) | 6.7 (5, 8.8) | -0.2 (-0.4, 0.1) |
| Bosnia and Herzegovina | 13 (10, 16) | 0.2 (0.2, 0.3) | 1 (0.5, 1.6) | 11 (9, 14) | 0.2 (0.2, 0.3) | 0.8 (0.4, 1.2) | 287 (222, 365) | 5.4 (4.2, 6.9) | 0.6 (0.2, 1) |
| Botswana | 7 (5, 10) | 0.4 (0.3, 0.6) | 0 (-0.4, 0.5) | 6 (4, 9) | 0.4 (0.3, 0.6) | -0.1 (-0.4, 0.4) | 218 (145, 306) | 12.1 (8.2, 16.7) | -0.1 (-0.4, 0.4) |
| Brazil | 675 (633, 721) | 0.3 (0.3, 0.3) | 0.2 (0.1, 0.3) | 527 (494, 560) | 0.2 (0.2, 0.2) | 0 (-0.1, 0.1) | 18249 (17075, 19340) | 7.5 (7.1, 8) | 0 (0, 0.1) |
| Brunei Darussalam | 22 (18, 27) | 5.3 (4.3, 6.4) | 0.1 (-0.1, 0.5) | 11 (9, 13) | 2.9 (2.5, 3.4) | -0.2 (-0.4, 0) | 391 (328, 462) | 89.8 (76.4, 105.6) | -0.2 (-0.4, 0) |
| Bulgaria | 72 (53, 97) | 0.7 (0.5, 1) | 1.3 (0.6, 2.2) | 44 (33, 56) | 0.4 (0.3, 0.5) | 0.7 (0.3, 1.3) | 1280 (961, 1671) | 12.1 (9.1, 15.7) | 0.7 (0.3, 1.3) |
| Burkina Faso | 38 (28, 50) | 0.3 (0.2, 0.4) | 0.3 (0, 0.8) | 36 (26, 47) | 0.3 (0.2, 0.4) | 0.3 (0, 0.8) | 1356 (971, 1842) | 10.4 (7.6, 13.7) | 0.3 (0, 0.9) |
| Burundi | 85 (58, 117) | 1.4 (1, 1.9) | -0.3 (-0.5, 0.1) | 80 (55, 111) | 1.4 (1, 1.9) | -0.3 (-0.5, 0.1) | 3019 (2048, 4157) | 45 (30.7, 62.7) | -0.3 (-0.5, 0.1) |
| Cabo Verde | 2 (1, 2) | 0.3 (0.3, 0.4) | 2.2 (1.4, 3.1) | 1 (1, 2) | 0.3 (0.2, 0.4) | 1.9 (1.2, 2.6) | 53 (42, 69) | 10.4 (8.2, 13.4) | 2 (1.2, 2.8) |
| Cambodia | 260 (193, 334) | 2 (1.5, 2.5) | -0.1 (-0.4, 0.2) | 249 (185, 316) | 2 (1.5, 2.5) | -0.1 (-0.4, 0.2) | 7940 (5899, 10319) | 57.2 (42.4, 73.8) | -0.2 (-0.4, 0.1) |
| Cameroon | 156 (103, 223) | 1 (0.7, 1.4) | 0 (-0.3, 0.4) | 143 (97, 204) | 1 (0.7, 1.3) | 0 (-0.3, 0.3) | 5599 (3638, 8074) | 31.1 (21, 44.7) | 0 (-0.3, 0.4) |
| Canada | 654 (480, 857) | 1.2 (0.9, 1.6) | 0.1 (-0.2, 0.4) | 136 (121, 153) | 0.2 (0.2, 0.3) | -0.3 (-0.4, -0.2) | 4088 (3576, 4671) | 7.5 (6.7, 8.6) | -0.3 (-0.4, -0.2) |
| Central African Republic | 18 (13, 26) | 0.7 (0.5, 0.9) | -0.2 (-0.4, 0.1) | 18 (12, 25) | 0.7 (0.5, 0.9) | -0.2 (-0.4, 0.1) | 649 (440, 934) | 20.9 (14.6, 28.8) | -0.2 (-0.4, 0.1) |
| Chad | 24 (17, 31) | 0.3 (0.2, 0.4) | 0.5 (0.1, 1) | 22 (17, 29) | 0.3 (0.2, 0.4) | 0.5 (0.1, 1) | 851 (617, 1119) | 10.4 (7.7, 13.7) | 0.5 (0.1, 1.1) |
| Chile | 54 (41, 73) | 0.2 (0.2, 0.3) | 0.2 (-0.1, 0.7) | 27 (23, 30) | 0.1 (0.1, 0.1) | -0.3 (-0.4, -0.2) | 746 (659, 848) | 3.3 (3, 3.8) | -0.3 (-0.4, -0.2) |
| China | 110433 (90342, 132397) | 5.7 (4.7, 6.8) | 0.7 (0.4, 1.2) | 28659 (23780, 34066) | 1.4 (1.2, 1.7) | -0.5 (-0.6, -0.4) | 912107 (760829, 1081932) | 45.6 (38.3, 53.8) | -0.5 (-0.6, -0.4) |
| Colombia | 119 (89, 155) | 0.2 (0.2, 0.3) | -0.3 (-0.5, -0.1) | 90 (68, 120) | 0.2 (0.1, 0.2) | -0.4 (-0.6, -0.3) | 2481 (1851, 3257) | 4.8 (3.6, 6.2) | -0.5 (-0.6, -0.3) |
| Comoros | 6 (4, 9) | 1.1 (0.8, 1.6) | -0.1 (-0.4, 0.9) | 6 (4, 9) | 1.1 (0.8, 1.6) | -0.1 (-0.4, 0.8) | 207 (138, 304) | 35.6 (24.5, 51.9) | -0.1 (-0.4, 1) |
| Congo | 18 (13, 24) | 0.6 (0.4, 0.8) | -0.3 (-0.5, 0.1) | 17 (12, 23) | 0.6 (0.4, 0.8) | -0.3 (-0.5, 0) | 588 (423, 814) | 16.7 (12.1, 22.9) | -0.3 (-0.6, 0) |
| Cook Islands | 0 (0, 0) | 0.3 (0.2, 0.4) | -0.2 (-0.4, 0.1) | 0 (0, 0) | 0.2 (0.2, 0.3) | -0.4 (-0.5, -0.1) | 1 (1, 2) | 6.4 (4.9, 8) | -0.4 (-0.5, -0.1) |
| Costa Rica | 36 (27, 49) | 0.7 (0.5, 1) | 0.4 (0, 1) | 20 (15, 26) | 0.4 (0.3, 0.5) | -0.1 (-0.3, 0.2) | 677 (507, 895) | 13.3 (10, 17.5) | 0 (-0.3, 0.4) |
| Croatia | 37 (27, 49) | 0.6 (0.4, 0.8) | -0.2 (-0.4, 0.2) | 18 (13, 23) | 0.2 (0.2, 0.3) | -0.5 (-0.6, -0.3) | 492 (369, 653) | 7.4 (5.5, 9.8) | -0.5 (-0.7, -0.3) |
| Cuba | 134 (105, 170) | 0.8 (0.6, 1) | 0.6 (0.2, 1) | 97 (76, 122) | 0.5 (0.4, 0.7) | 0.2 (0, 0.6) | 2551 (2003, 3263) | 14.6 (11.5, 18.4) | 0.2 (0, 0.6) |
| Cyprus | 12 (10, 15) | 0.7 (0.6, 0.9) | 1.6 (1, 2.3) | 3 (2, 3) | 0.2 (0.1, 0.2) | -0.2 (-0.3, 0) | 90 (76, 106) | 5.2 (4.4, 6.1) | -0.1 (-0.3, 0.1) |
| Czechia | 63 (49, 80) | 0.4 (0.3, 0.5) | -0.2 (-0.4, 0) | 39 (30, 49) | 0.2 (0.2, 0.3) | -0.5 (-0.6, -0.4) | 1078 (841, 1365) | 6.6 (5.2, 8.4) | -0.5 (-0.6, -0.4) |
| Democratic People's Republic of Korea | 583 (426, 772) | 1.8 (1.3, 2.3) | -0.2 (-0.4, 0.1) | 525 (394, 676) | 1.6 (1.2, 2.1) | -0.2 (-0.4, 0.1) | 17041 (12097, 22822) | 51.4 (36.7, 68.7) | -0.2 (-0.5, 0.1) |
| Democratic Republic of the Congo | 210 (154, 275) | 0.5 (0.4, 0.6) | -0.1 (-0.4, 0.2) | 200 (147, 263) | 0.5 (0.4, 0.6) | -0.2 (-0.4, 0.2) | 7085 (5263, 9256) | 14.5 (10.6, 19.1) | -0.2 (-0.4, 0.2) |
| Denmark | 54 (40, 72) | 0.6 (0.5, 0.8) | 0.6 (0.2, 1.3) | 14 (12, 16) | 0.1 (0.1, 0.2) | -0.2 (-0.3, 0) | 384 (330, 445) | 4.4 (3.8, 5.1) | -0.2 (-0.3, 0) |
| Djibouti | 10 (6, 15) | 1.3 (0.9, 1.9) | -0.1 (-0.4, 0.3) | 10 (6, 14) | 1.3 (0.9, 1.8) | -0.1 (-0.4, 0.3) | 355 (220, 540) | 40.2 (26, 59.2) | -0.1 (-0.4, 0.3) |
| Dominica | 1 (0, 1) | 0.6 (0.5, 0.8) | 0.4 (0, 0.8) | 1 (0, 1) | 0.6 (0.5, 0.8) | 0.4 (0, 0.8) | 14 (11, 18) | 16.8 (13.3, 21.5) | 0.4 (0, 0.8) |
| Dominican Republic | 43 (32, 56) | 0.5 (0.3, 0.6) | 0.4 (0, 0.8) | 42 (32, 54) | 0.5 (0.3, 0.6) | 0.3 (-0.1, 0.7) | 1114 (812, 1475) | 11.3 (8.3, 14.9) | 0.3 (-0.1, 0.7) |
| Ecuador | 29 (23, 38) | 0.2 (0.1, 0.2) | 0.2 (-0.1, 0.5) | 26 (20, 34) | 0.2 (0.1, 0.2) | 0.1 (-0.2, 0.4) | 733 (564, 956) | 4.5 (3.5, 5.9) | -0.1 (-0.3, 0.2) |
| Egypt | 151 (107, 206) | 0.2 (0.1, 0.3) | 0.3 (-0.1, 0.9) | 84 (60, 113) | 0.1 (0.1, 0.2) | -0.1 (-0.3, 0.3) | 3110 (2251, 4172) | 3.7 (2.7, 5) | -0.1 (-0.4, 0.2) |
| El Salvador | 14 (10, 18) | 0.2 (0.2, 0.3) | 0.1 (-0.2, 0.5) | 12 (9, 16) | 0.2 (0.2, 0.3) | 0 (-0.3, 0.3) | 330 (247, 431) | 5.6 (4.1, 7.3) | -0.1 (-0.3, 0.2) |
| Equatorial Guinea | 3 (2, 4) | 0.4 (0.3, 0.7) | -0.3 (-0.6, 0.2) | 2 (1, 4) | 0.4 (0.3, 0.7) | -0.3 (-0.6, 0.2) | 87 (49, 145) | 12.4 (7.2, 20.5) | -0.4 (-0.7, 0.1) |
| Eritrea | 53 (34, 75) | 1.4 (1, 2) | -0.1 (-0.4, 0.3) | 49 (32, 69) | 1.4 (0.9, 1.9) | -0.1 (-0.4, 0.3) | 1903 (1225, 2702) | 46.1 (30.2, 64.4) | -0.1 (-0.4, 0.3) |
| Estonia | 9 (6, 12) | 0.5 (0.3, 0.7) | -0.3 (-0.5, -0.1) | 6 (4, 7) | 0.3 (0.2, 0.4) | -0.6 (-0.7, -0.4) | 166 (121, 221) | 8.8 (6.4, 11.7) | -0.6 (-0.7, -0.4) |
| Eswatini | 5 (3, 7) | 0.7 (0.5, 1) | 0 (-0.3, 0.5) | 5 (3, 6) | 0.7 (0.5, 1) | 0 (-0.3, 0.5) | 155 (106, 221) | 21.1 (14.6, 29.8) | 0 (-0.3, 0.5) |
| Ethiopia | 534 (340, 700) | 1 (0.6, 1.4) | -0.4 (-0.6, 0.1) | 507 (318, 670) | 1 (0.6, 1.4) | -0.4 (-0.6, 0.1) | 18447 (11652, 24529) | 31.8 (19.9, 42.1) | -0.4 (-0.6, 0.1) |
| Fiji | 3 (2, 4) | 0.4 (0.3, 0.5) | -0.2 (-0.4, 0.2) | 3 (2, 4) | 0.4 (0.3, 0.5) | -0.2 (-0.4, 0.2) | 99 (75, 130) | 11.3 (8.6, 14.6) | -0.2 (-0.4, 0.2) |
| Finland | 41 (31, 54) | 0.5 (0.4, 0.6) | 0.4 (0, 0.9) | 10 (9, 12) | 0.1 (0.1, 0.1) | -0.3 (-0.4, -0.2) | 277 (239, 323) | 3.2 (2.8, 3.8) | -0.3 (-0.4, -0.2) |
| France | 1578 (1127, 2159) | 1.6 (1.1, 2.1) | -0.2 (-0.4, 0.2) | 388 (341, 442) | 0.3 (0.3, 0.4) | -0.6 (-0.7, -0.6) | 11083 (9634, 12754) | 11.2 (9.7, 12.9) | -0.6 (-0.7, -0.6) |
| Gabon | 7 (5, 9) | 0.6 (0.4, 0.8) | -0.2 (-0.5, 0.2) | 6 (4, 9) | 0.6 (0.4, 0.7) | -0.2 (-0.5, 0.1) | 212 (148, 291) | 16.3 (11.3, 22.4) | -0.3 (-0.5, 0.1) |
| Gambia | 3 (2, 4) | 0.2 (0.2, 0.3) | 0.2 (-0.2, 0.8) | 3 (2, 3) | 0.2 (0.2, 0.3) | 0.2 (-0.2, 0.7) | 98 (70, 133) | 7.5 (5.4, 10.2) | 0.2 (-0.2, 0.8) |
| Georgia | 17 (14, 20) | 0.3 (0.3, 0.4) | -0.1 (-0.3, 0.1) | 16 (13, 19) | 0.3 (0.2, 0.3) | -0.2 (-0.3, 0.1) | 457 (375, 552) | 9.3 (7.6, 11.2) | -0.2 (-0.4, 0.1) |
| Germany | 1211 (896, 1629) | 0.9 (0.6, 1.2) | 0.1 (-0.2, 0.5) | 281 (247, 314) | 0.2 (0.2, 0.2) | -0.4 (-0.5, -0.4) | 7756 (6747, 8811) | 5.6 (4.9, 6.3) | -0.5 (-0.5, -0.4) |
| Ghana | 32 (24, 43) | 0.2 (0.1, 0.2) | -0.4 (-0.6, -0.1) | 30 (22, 39) | 0.2 (0.1, 0.2) | -0.4 (-0.6, -0.1) | 1134 (827, 1493) | 5 (3.7, 6.6) | -0.5 (-0.6, -0.2) |
| Greece | 290 (220, 382) | 1.7 (1.3, 2.3) | 0.7 (0.3, 1.4) | 80 (73, 88) | 0.4 (0.4, 0.4) | 0.1 (-0.1, 0.2) | 2148 (1944, 2386) | 13 (11.8, 14.4) | 0.1 (0, 0.2) |
| Greenland | 4 (4, 6) | 6 (4.8, 7.4) | -0.5 (-0.6, -0.3) | 3 (3, 4) | 4.7 (3.8, 5.7) | -0.5 (-0.6, -0.4) | 105 (82, 131) | 137.7 (109.3, 171.5) | -0.6 (-0.7, -0.4) |
| Grenada | 1 (1, 1) | 0.6 (0.5, 0.7) | 0 (-0.1, 0.2) | 1 (1, 1) | 0.6 (0.5, 0.6) | 0 (-0.2, 0.1) | 19 (16, 22) | 16.1 (13.9, 18.3) | -0.1 (-0.2, 0.1) |
| Guam | 6 (4, 7) | 2.9 (2.3, 3.6) | 0 (-0.2, 0.4) | 5 (4, 6) | 2.5 (2, 3.1) | 0 (-0.3, 0.3) | 151 (120, 187) | 77.6 (62.2, 95.5) | 0 (-0.2, 0.4) |
| Guatemala | 31 (24, 38) | 0.3 (0.2, 0.3) | 0.5 (0.1, 0.9) | 29 (22, 36) | 0.3 (0.2, 0.3) | 0.4 (0.1, 0.8) | 885 (687, 1115) | 6.7 (5.2, 8.4) | 0.3 (0, 0.7) |
| Guinea | 20 (15, 26) | 0.3 (0.2, 0.4) | 0.1 (-0.2, 0.5) | 19 (15, 25) | 0.3 (0.2, 0.4) | 0.1 (-0.2, 0.5) | 717 (540, 946) | 9.7 (7.2, 12.6) | 0.1 (-0.2, 0.5) |
| Guinea-Bissau | 4 (3, 6) | 0.4 (0.3, 0.6) | 0.2 (-0.2, 0.7) | 4 (3, 6) | 0.4 (0.3, 0.6) | 0.1 (-0.2, 0.6) | 158 (109, 221) | 14.5 (9.9, 20.2) | 0.2 (-0.2, 0.7) |
| Guyana | 2 (2, 3) | 0.3 (0.3, 0.5) | 0.2 (-0.1, 0.6) | 2 (2, 3) | 0.3 (0.3, 0.4) | 0.2 (-0.1, 0.6) | 75 (55, 98) | 10.2 (7.6, 13.3) | 0.2 (-0.1, 0.6) |
| Haiti | 61 (38, 85) | 0.8 (0.5, 1) | 0 (-0.3, 0.3) | 58 (36, 81) | 0.8 (0.5, 1) | 0 (-0.3, 0.3) | 2039 (1231, 2843) | 22.6 (14, 31.4) | -0.1 (-0.3, 0.3) |
| Honduras | 11 (8, 14) | 0.2 (0.1, 0.2) | 0.1 (-0.2, 0.4) | 10 (7, 13) | 0.2 (0.1, 0.2) | 0 (-0.2, 0.4) | 308 (221, 408) | 4.4 (3.2, 5.8) | -0.1 (-0.3, 0.2) |
| Hungary | 127 (98, 161) | 0.9 (0.7, 1.1) | 0.5 (0.1, 0.9) | 87 (69, 109) | 0.5 (0.4, 0.7) | 0.1 (-0.2, 0.4) | 2619 (2044, 3320) | 17.8 (13.9, 22.6) | 0 (-0.2, 0.3) |
| Iceland | 4 (3, 5) | 0.8 (0.7, 1) | 0.1 (-0.2, 0.4) | 1 (1, 1) | 0.2 (0.1, 0.2) | -0.4 (-0.5, -0.2) | 23 (20, 27) | 5 (4.3, 5.8) | -0.3 (-0.5, -0.2) |
| India | 12212 (10384, 14415) | 1 (0.8, 1.1) | -0.3 (-0.4, -0.1) | 11358 (9717, 13434) | 0.9 (0.8, 1.1) | -0.3 (-0.4, -0.1) | 388094 (331090, 459149) | 29.7 (25.5, 35.2) | -0.3 (-0.4, -0.1) |
| Indonesia | 3356 (2639, 4364) | 1.4 (1.1, 1.8) | -0.1 (-0.2, 0.2) | 3217 (2507, 4134) | 1.4 (1.1, 1.8) | 0 (-0.2, 0.2) | 100797 (78200, 131184) | 39.7 (31, 51) | -0.1 (-0.3, 0.1) |
| Iran (Islamic Republic of) | 339 (307, 377) | 0.4 (0.4, 0.5) | 0.2 (0, 0.5) | 129 (120, 139) | 0.2 (0.2, 0.2) | -0.4 (-0.4, -0.2) | 4110 (3839, 4438) | 5 (4.7, 5.4) | -0.4 (-0.4, -0.2) |
| Iraq | 139 (98, 186) | 0.4 (0.3, 0.6) | -0.2 (-0.5, 0.1) | 60 (44, 78) | 0.2 (0.2, 0.3) | -0.5 (-0.7, -0.4) | 2220 (1612, 2924) | 7.1 (5.2, 9.2) | -0.6 (-0.7, -0.4) |
| Ireland | 58 (43, 78) | 0.9 (0.7, 1.2) | 0.4 (0.1, 1) | 13 (11, 15) | 0.2 (0.2, 0.2) | -0.4 (-0.5, -0.3) | 366 (311, 428) | 5.6 (4.8, 6.5) | -0.4 (-0.5, -0.3) |
| Israel | 67 (50, 88) | 0.7 (0.5, 0.9) | 0.1 (-0.2, 0.5) | 19 (17, 22) | 0.2 (0.2, 0.2) | -0.5 (-0.6, -0.5) | 576 (509, 655) | 5.7 (5.1, 6.5) | -0.5 (-0.6, -0.4) |
| Italy | 1424 (1125, 1779) | 1.4 (1.1, 1.8) | 0.2 (0, 0.6) | 320 (298, 341) | 0.3 (0.3, 0.3) | -0.4 (-0.4, -0.4) | 9119 (8456, 9828) | 9.3 (8.7, 10) | -0.4 (-0.4, -0.3) |
| Jamaica | 20 (15, 25) | 0.6 (0.5, 0.8) | 1 (0.5, 1.7) | 15 (12, 19) | 0.5 (0.4, 0.6) | 0.8 (0.3, 1.3) | 511 (390, 654) | 17.1 (13.1, 21.9) | 0.9 (0.4, 1.4) |
| Japan | 3985 (3238, 4847) | 1.5 (1.2, 1.8) | 0.3 (0.1, 0.6) | 936 (829, 1018) | 0.3 (0.3, 0.3) | 0.1 (0, 0.2) | 20308 (18438, 22292) | 8 (7.4, 8.7) | 0 (-0.1, 0.1) |
| Jordan | 80 (64, 98) | 0.9 (0.8, 1.1) | -0.1 (-0.3, 0.2) | 32 (25, 39) | 0.4 (0.4, 0.5) | -0.5 (-0.6, -0.3) | 1095 (877, 1351) | 12.6 (10.1, 15.6) | -0.5 (-0.6, -0.4) |
| Kazakhstan | 91 (76, 110) | 0.5 (0.4, 0.6) | 0.1 (0, 0.4) | 77 (64, 92) | 0.4 (0.4, 0.5) | 0 (-0.1, 0.2) | 2492 (2053, 2990) | 13.3 (11, 15.9) | 0 (-0.2, 0.2) |
| Kenya | 478 (312, 629) | 1.7 (1.1, 2.2) | 0.1 (-0.1, 0.3) | 469 (312, 620) | 1.7 (1.1, 2.2) | 0.2 (-0.1, 0.4) | 17394 (11621, 23030) | 55 (36.5, 72.7) | 0.2 (-0.1, 0.4) |
| Kiribati | 1 (1, 2) | 1.4 (1, 1.9) | -0.1 (-0.3, 0.4) | 1 (1, 2) | 1.4 (1, 1.8) | -0.1 (-0.4, 0.3) | 41 (29, 55) | 44.4 (32.1, 59.7) | -0.1 (-0.4, 0.4) |
| Kuwait | 25 (20, 31) | 0.7 (0.5, 0.8) | -0.5 (-0.6, -0.3) | 7 (6, 9) | 0.2 (0.2, 0.3) | -0.6 (-0.7, -0.4) | 265 (212, 332) | 7 (5.6, 8.8) | -0.6 (-0.7, -0.5) |
| Kyrgyzstan | 22 (19, 26) | 0.4 (0.3, 0.5) | 0.2 (-0.1, 0.4) | 19 (16, 23) | 0.4 (0.3, 0.4) | 0.1 (-0.1, 0.4) | 694 (577, 824) | 12.1 (10.1, 14.4) | 0.1 (-0.2, 0.3) |
| Lao People's Democratic Republic | 80 (57, 106) | 1.6 (1.1, 2.1) | -0.4 (-0.6, -0.1) | 77 (55, 102) | 1.6 (1.1, 2.1) | -0.4 (-0.5, -0.1) | 2587 (1797, 3480) | 47.1 (33.4, 62.5) | -0.4 (-0.6, -0.1) |
| Latvia | 11 (8, 14) | 0.4 (0.3, 0.5) | -0.4 (-0.6, -0.2) | 9 (7, 11) | 0.3 (0.2, 0.3) | -0.5 (-0.6, -0.4) | 246 (186, 320) | 8.4 (6.4, 11) | -0.5 (-0.7, -0.4) |
| Lebanon | 72 (54, 97) | 1.4 (1, 1.8) | 0.5 (0.1, 1.3) | 23 (18, 30) | 0.4 (0.3, 0.6) | -0.4 (-0.5, -0.1) | 742 (568, 983) | 14.2 (10.9, 18.8) | -0.3 (-0.5, -0.1) |
| Lesotho | 13 (9, 17) | 0.9 (0.7, 1.2) | 0.4 (0, 0.9) | 13 (9, 17) | 0.9 (0.7, 1.3) | 0.4 (0, 0.9) | 420 (295, 567) | 27.7 (19.4, 37.1) | 0.4 (0, 1) |
| Liberia | 8 (6, 11) | 0.3 (0.2, 0.4) | 0.1 (-0.2, 0.6) | 8 (5, 11) | 0.3 (0.2, 0.4) | 0.1 (-0.3, 0.6) | 300 (203, 414) | 9.5 (6.6, 13.3) | 0.1 (-0.3, 0.6) |
| Libya | 326 (233, 445) | 4.8 (3.5, 6.3) | 0.4 (0, 1.1) | 133 (102, 171) | 2.2 (1.7, 2.9) | -0.1 (-0.4, 0.2) | 4772 (3600, 6218) | 70.9 (54.6, 91.3) | -0.1 (-0.4, 0.3) |
| Lithuania | 15 (11, 20) | 0.4 (0.3, 0.5) | -0.4 (-0.6, -0.2) | 12 (9, 16) | 0.3 (0.2, 0.3) | -0.5 (-0.6, -0.3) | 351 (262, 459) | 8.3 (6.2, 10.8) | -0.5 (-0.6, -0.4) |
| Luxembourg | 13 (10, 17) | 1.5 (1.2, 2) | 0.1 (-0.2, 0.5) | 3 (3, 4) | 0.3 (0.3, 0.4) | -0.5 (-0.6, -0.4) | 92 (77, 111) | 10.5 (8.7, 12.6) | -0.5 (-0.6, -0.4) |
| Madagascar | 155 (106, 216) | 1 (0.7, 1.5) | -0.2 (-0.4, 0.1) | 146 (100, 203) | 1 (0.7, 1.4) | -0.2 (-0.4, 0.1) | 5587 (3875, 7768) | 33.6 (23, 46.9) | -0.2 (-0.4, 0.1) |
| Malawi | 27 (20, 38) | 0.3 (0.2, 0.4) | -0.2 (-0.5, 0.1) | 26 (18, 35) | 0.3 (0.2, 0.4) | -0.2 (-0.4, 0.1) | 988 (691, 1392) | 9.3 (6.7, 12.8) | -0.2 (-0.5, 0.1) |
| Malaysia | 1846 (1382, 2378) | 6.1 (4.6, 7.8) | -0.2 (-0.4, 0.1) | 1380 (1053, 1767) | 4.8 (3.6, 6.1) | -0.3 (-0.5, -0.1) | 46906 (35572, 60317) | 152.3 (115.7, 195.1) | -0.3 (-0.5, -0.1) |
| Maldives | 1 (1, 1) | 0.3 (0.2, 0.3) | -0.4 (-0.6, -0.1) | 1 (1, 1) | 0.2 (0.2, 0.2) | -0.5 (-0.7, -0.3) | 21 (17, 26) | 5.3 (4.3, 6.4) | -0.6 (-0.7, -0.3) |
| Mali | 16 (12, 22) | 0.1 (0.1, 0.2) | -0.1 (-0.3, 0.3) | 15 (11, 20) | 0.1 (0.1, 0.2) | -0.1 (-0.3, 0.3) | 591 (427, 806) | 4.7 (3.5, 6.4) | -0.1 (-0.3, 0.3) |
| Malta | 20 (16, 25) | 3 (2.4, 3.7) | 0.4 (0.1, 0.8) | 6 (5, 7) | 0.7 (0.6, 0.8) | -0.4 (-0.5, -0.3) | 158 (134, 183) | 23.2 (19.7, 26.9) | -0.3 (-0.4, -0.2) |
| Marshall Islands | 1 (1, 1) | 1.8 (1.2, 2.6) | 0 (-0.3, 0.5) | 1 (0, 1) | 1.8 (1.2, 2.5) | 0 (-0.3, 0.4) | 25 (17, 37) | 54.7 (37.4, 80.2) | 0 (-0.3, 0.5) |
| Mauritania | 6 (4, 9) | 0.2 (0.1, 0.3) | -0.1 (-0.4, 0.3) | 5 (3, 8) | 0.2 (0.1, 0.3) | -0.1 (-0.4, 0.3) | 188 (113, 292) | 7.1 (4.4, 10.9) | -0.1 (-0.5, 0.3) |
| Mauritius | 14 (11, 17) | 0.8 (0.6, 1) | 0.1 (-0.2, 0.4) | 12 (9, 15) | 0.7 (0.5, 0.8) | -0.1 (-0.3, 0.2) | 338 (266, 432) | 19.6 (15.5, 25) | 0 (-0.2, 0.2) |
| Mexico | 213 (181, 251) | 0.2 (0.1, 0.2) | 0 (-0.2, 0.1) | 174 (146, 204) | 0.1 (0.1, 0.2) | -0.2 (-0.3, 0) | 5187 (4345, 6106) | 4.2 (3.5, 4.9) | -0.2 (-0.3, 0) |
| Micronesia (Federated States of) | 1 (1, 2) | 1.6 (1.1, 2.3) | -0.1 (-0.4, 0.3) | 1 (1, 2) | 1.6 (1.1, 2.3) | -0.1 (-0.4, 0.2) | 44 (27, 66) | 49.4 (31.5, 72.6) | -0.2 (-0.5, 0.2) |
| Monaco | 1 (1, 1) | 1.2 (0.9, 1.5) | 0.3 (-0.1, 0.8) | 0 (0, 0) | 0.2 (0.2, 0.3) | -0.1 (-0.4, 0.2) | 5 (3, 6) | 7.2 (5.5, 9.2) | -0.1 (-0.4, 0.2) |
| Mongolia | 9 (7, 12) | 0.3 (0.2, 0.4) | 0.1 (-0.2, 0.6) | 8 (6, 11) | 0.3 (0.2, 0.4) | 0.1 (-0.2, 0.5) | 305 (224, 406) | 9.4 (7, 12.3) | 0.1 (-0.3, 0.5) |
| Montenegro | 2 (1, 2) | 0.2 (0.2, 0.2) | 0.2 (-0.1, 0.5) | 1 (1, 1) | 0.1 (0.1, 0.1) | 0 (-0.2, 0.2) | 33 (27, 41) | 3.9 (3.1, 4.9) | -0.1 (-0.3, 0.2) |
| Morocco | 957 (701, 1279) | 2.7 (2, 3.5) | 0.1 (-0.2, 0.5) | 675 (493, 878) | 2 (1.5, 2.5) | -0.1 (-0.4, 0.2) | 22370 (16226, 30657) | 61.8 (45.4, 83.2) | -0.2 (-0.4, 0.2) |
| Mozambique | 20 (15, 26) | 0.1 (0.1, 0.2) | 0.2 (-0.1, 0.6) | 19 (14, 25) | 0.1 (0.1, 0.2) | 0.2 (-0.1, 0.6) | 742 (550, 960) | 4.5 (3.3, 5.8) | 0.2 (-0.1, 0.7) |
| Myanmar | 720 (570, 911) | 1.4 (1.2, 1.8) | -0.4 (-0.5, -0.1) | 693 (554, 869) | 1.4 (1.2, 1.8) | -0.4 (-0.5, -0.1) | 22101 (17233, 28381) | 42 (33.1, 53.3) | -0.4 (-0.6, -0.2) |
| Namibia | 9 (7, 12) | 0.6 (0.4, 0.7) | 0 (-0.3, 0.5) | 8 (6, 11) | 0.5 (0.4, 0.7) | 0 (-0.3, 0.4) | 299 (212, 416) | 17 (12.3, 23.3) | 0 (-0.3, 0.5) |
| Nauru | 0 (0, 0) | 1.7 (1.1, 2.4) | -0.1 (-0.3, 0.2) | 0 (0, 0) | 1.6 (1.1, 2.2) | -0.1 (-0.3, 0.1) | 3 (2, 5) | 49.6 (32.6, 70.8) | -0.1 (-0.4, 0.1) |
| Nepal | 244 (180, 308) | 1 (0.7, 1.3) | -0.2 (-0.5, 0.1) | 232 (172, 293) | 1 (0.7, 1.2) | -0.2 (-0.5, 0.1) | 7627 (5542, 9745) | 29.9 (21.8, 38.1) | -0.3 (-0.5, 0.1) |
| Netherlands | 332 (246, 438) | 1.2 (0.9, 1.6) | 0.4 (0, 0.8) | 74 (65, 84) | 0.2 (0.2, 0.3) | -0.2 (-0.3, -0.1) | 2013 (1756, 2312) | 7.4 (6.5, 8.5) | -0.2 (-0.3, -0.1) |
| New Zealand | 43 (35, 51) | 0.7 (0.5, 0.8) | 0 (-0.2, 0.2) | 20 (18, 22) | 0.3 (0.3, 0.3) | -0.3 (-0.3, -0.2) | 577 (528, 633) | 9.4 (8.6, 10.3) | -0.3 (-0.3, -0.2) |
| Nicaragua | 16 (13, 20) | 0.3 (0.3, 0.4) | 0.4 (0.1, 0.7) | 13 (10, 15) | 0.3 (0.2, 0.3) | 0.2 (-0.1, 0.5) | 395 (311, 494) | 7.5 (6, 9.3) | 0.1 (-0.2, 0.3) |
| Niger | 12 (8, 16) | 0.1 (0.1, 0.2) | -0.1 (-0.4, 0.2) | 11 (8, 15) | 0.1 (0.1, 0.1) | -0.1 (-0.4, 0.2) | 443 (308, 614) | 3.6 (2.5, 4.9) | -0.1 (-0.4, 0.2) |
| Nigeria | 935 (687, 1228) | 0.8 (0.6, 1.1) | 0 (-0.3, 0.4) | 885 (650, 1151) | 0.8 (0.6, 1.1) | -0.1 (-0.3, 0.3) | 32221 (23406, 42554) | 26.2 (19.3, 34) | -0.1 (-0.3, 0.4) |
| Niue | 0 (0, 0) | 1.2 (0.9, 1.6) | -0.1 (-0.4, 0.2) | 0 (0, 0) | 1 (0.8, 1.3) | -0.2 (-0.4, 0.1) | 1 (0, 1) | 30.7 (22.3, 40.6) | -0.2 (-0.4, 0.1) |
| North Macedonia | 10 (8, 13) | 0.4 (0.3, 0.5) | 0 (-0.2, 0.3) | 8 (6, 10) | 0.3 (0.2, 0.3) | -0.2 (-0.4, 0.1) | 245 (189, 317) | 8.2 (6.4, 10.6) | -0.2 (-0.4, 0) |
| Northern Mariana Islands | 2 (2, 2) | 3.1 (2.5, 3.8) | 0.2 (-0.1, 0.5) | 2 (1, 2) | 2.6 (2.1, 3) | 0.1 (-0.1, 0.4) | 49 (39, 60) | 74.4 (60.3, 89.9) | 0.1 (-0.2, 0.4) |
| Norway | 40 (33, 49) | 0.5 (0.4, 0.6) | 0.1 (-0.1, 0.4) | 9 (8, 10) | 0.1 (0.1, 0.1) | -0.4 (-0.4, -0.3) | 257 (236, 283) | 3.4 (3.1, 3.7) | -0.4 (-0.4, -0.3) |
| Oman | 22 (17, 28) | 0.7 (0.6, 0.9) | 0.1 (-0.2, 0.6) | 7 (5, 9) | 0.3 (0.2, 0.4) | -0.5 (-0.6, -0.2) | 256 (190, 346) | 8.6 (6.7, 11.1) | -0.5 (-0.6, -0.2) |
| Pakistan | 2012 (1601, 2497) | 1.4 (1.1, 1.7) | 0 (-0.2, 0.3) | 1859 (1481, 2325) | 1.4 (1.1, 1.7) | 0 (-0.3, 0.2) | 71647 (57194, 88599) | 45.7 (36.3, 57) | 0 (-0.2, 0.3) |
| Palau | 0 (0, 0) | 0.3 (0.2, 0.4) | 0 (-0.3, 0.3) | 0 (0, 0) | 0.2 (0.2, 0.3) | -0.1 (-0.4, 0.2) | 2 (1, 3) | 7.5 (5.6, 9.8) | -0.1 (-0.4, 0.2) |
| Palestine | 15 (12, 18) | 0.4 (0.4, 0.5) | 0.1 (-0.2, 0.6) | 7 (6, 8) | 0.3 (0.2, 0.3) | -0.2 (-0.5, 0.1) | 257 (212, 309) | 7.7 (6.4, 9.3) | -0.2 (-0.5, 0.1) |
| Panama | 16 (12, 21) | 0.4 (0.3, 0.5) | 0.2 (-0.1, 0.7) | 12 (9, 16) | 0.3 (0.2, 0.4) | 0 (-0.3, 0.3) | 358 (267, 473) | 8.5 (6.4, 11.3) | 0 (-0.3, 0.4) |
| Papua New Guinea | 74 (50, 102) | 1.3 (0.9, 1.7) | 0 (-0.2, 0.4) | 70 (48, 97) | 1.3 (0.9, 1.7) | 0 (-0.2, 0.4) | 2455 (1662, 3432) | 38.1 (26.4, 52.3) | 0 (-0.3, 0.4) |
| Paraguay | 12 (9, 16) | 0.2 (0.1, 0.3) | 0.3 (-0.1, 0.8) | 10 (7, 13) | 0.2 (0.1, 0.2) | 0.2 (-0.2, 0.6) | 335 (246, 444) | 5.3 (3.9, 7) | 0.1 (-0.2, 0.6) |
| Peru | 49 (36, 65) | 0.1 (0.1, 0.2) | -0.1 (-0.4, 0.3) | 41 (30, 54) | 0.1 (0.1, 0.2) | -0.3 (-0.5, 0) | 1157 (836, 1536) | 3.5 (2.5, 4.6) | -0.3 (-0.5, 0) |
| Philippines | 1559 (1260, 1924) | 1.8 (1.4, 2.2) | -0.2 (-0.4, 0.1) | 1432 (1158, 1747) | 1.7 (1.4, 2) | -0.2 (-0.4, 0) | 48258 (38808, 59051) | 51.2 (41.4, 62.5) | -0.2 (-0.4, 0) |
| Poland | 260 (213, 314) | 0.4 (0.4, 0.5) | 0.2 (-0.1, 0.4) | 219 (181, 265) | 0.3 (0.3, 0.4) | -0.1 (-0.2, 0.2) | 6081 (4965, 7451) | 10.7 (8.7, 13.1) | -0.1 (-0.3, 0.1) |
| Portugal | 254 (187, 336) | 1.6 (1.2, 2.1) | 1 (0.4, 1.7) | 77 (67, 87) | 0.4 (0.4, 0.5) | -0.2 (-0.3, -0.1) | 2313 (2021, 2621) | 14.3 (12.4, 16.3) | -0.2 (-0.3, -0.1) |
| Puerto Rico | 34 (25, 44) | 0.6 (0.4, 0.8) | 0 (-0.3, 0.3) | 22 (16, 28) | 0.3 (0.2, 0.4) | -0.4 (-0.5, -0.2) | 513 (385, 679) | 9 (6.7, 12) | -0.3 (-0.5, -0.1) |
| Qatar | 14 (10, 20) | 0.8 (0.6, 1.1) | 0.4 (-0.1, 1.2) | 4 (3, 6) | 0.3 (0.2, 0.5) | -0.3 (-0.6, 0.1) | 168 (115, 243) | 9 (6.4, 12.7) | -0.4 (-0.6, 0.1) |
| Republic of Korea | 1087 (869, 1346) | 1.3 (1, 1.6) | 1.3 (0.7, 2.1) | 200 (174, 227) | 0.2 (0.2, 0.3) | -0.4 (-0.5, -0.3) | 6115 (5289, 7017) | 7.3 (6.4, 8.4) | -0.4 (-0.5, -0.2) |
| Republic of Moldova | 36 (30, 44) | 0.7 (0.6, 0.9) | 0.3 (0.1, 0.5) | 31 (25, 37) | 0.6 (0.5, 0.7) | 0.1 (-0.1, 0.4) | 1013 (842, 1231) | 20.4 (17.1, 24.6) | 0.1 (-0.1, 0.3) |
| Romania | 280 (224, 345) | 1 (0.8, 1.3) | 2 (1.3, 2.7) | 211 (168, 257) | 0.7 (0.6, 0.8) | 1.2 (0.8, 1.7) | 6582 (5236, 8105) | 24.1 (19.2, 29.5) | 1.2 (0.7, 1.7) |
| Russian Federation | 710 (592, 850) | 0.4 (0.3, 0.4) | 0 (-0.1, 0.2) | 535 (448, 638) | 0.2 (0.2, 0.3) | -0.2 (-0.3, -0.1) | 16687 (13900, 19953) | 8.2 (6.9, 9.8) | -0.2 (-0.3, -0.1) |
| Rwanda | 92 (62, 128) | 1.2 (0.8, 1.7) | -0.4 (-0.6, -0.1) | 86 (59, 120) | 1.2 (0.8, 1.6) | -0.4 (-0.6, -0.1) | 3166 (2122, 4502) | 38 (25.8, 52.9) | -0.4 (-0.6, -0.1) |
| Saint Kitts and Nevis | 1 (1, 1) | 1 (0.8, 1.2) | 0.1 (-0.2, 0.4) | 1 (0, 1) | 0.8 (0.7, 1) | 0 (-0.2, 0.2) | 16 (12, 20) | 22.3 (17, 27.7) | -0.1 (-0.3, 0.2) |
| Saint Lucia | 2 (1, 2) | 0.8 (0.7, 1) | 0.2 (-0.1, 0.4) | 2 (1, 2) | 0.7 (0.6, 0.9) | 0 (-0.1, 0.3) | 44 (36, 53) | 20.4 (16.9, 24.5) | 0.1 (-0.1, 0.3) |
| Saint Vincent and the Grenadines | 1 (1, 1) | 0.7 (0.6, 0.8) | 0.2 (0, 0.5) | 1 (1, 1) | 0.7 (0.6, 0.8) | 0.2 (0, 0.4) | 25 (21, 29) | 18.4 (15.9, 21.5) | 0.2 (0, 0.5) |
| Samoa | 3 (2, 4) | 1.7 (1.3, 2.3) | -0.2 (-0.4, 0.2) | 3 (2, 3) | 1.6 (1.2, 2.1) | -0.2 (-0.4, 0.1) | 91 (66, 127) | 52.4 (38.3, 72.8) | -0.2 (-0.4, 0.2) |
| San Marino | 1 (1, 2) | 2.7 (1.9, 3.6) | 0.2 (-0.2, 0.8) | 0 (0, 0) | 0.6 (0.4, 0.8) | -0.2 (-0.5, 0.2) | 9 (6, 13) | 17.3 (10.9, 26.2) | -0.2 (-0.5, 0.2) |
| Sao Tome and Principe | 0 (0, 0) | 0.2 (0.1, 0.2) | 0.2 (-0.1, 0.7) | 0 (0, 0) | 0.1 (0.1, 0.2) | 0.2 (-0.1, 0.6) | 7 (5, 9) | 4.8 (3.7, 6.2) | 0.1 (-0.2, 0.7) |
| Saudi Arabia | 694 (511, 935) | 2.1 (1.6, 2.7) | 0.5 (0.1, 1.3) | 207 (157, 269) | 0.8 (0.6, 1) | -0.4 (-0.6, -0.1) | 8307 (6203, 11023) | 25.5 (19.7, 32.5) | -0.4 (-0.6, -0.1) |
| Senegal | 27 (19, 38) | 0.3 (0.2, 0.4) | 0.2 (-0.1, 0.7) | 26 (18, 36) | 0.3 (0.2, 0.4) | 0.2 (-0.1, 0.7) | 945 (649, 1335) | 9.5 (6.5, 13.4) | 0.2 (-0.2, 0.8) |
| Serbia | 82 (62, 109) | 0.7 (0.5, 0.9) | 0.5 (0.1, 1) | 48 (37, 62) | 0.4 (0.3, 0.5) | -0.1 (-0.3, 0.2) | 1416 (1080, 1830) | 11.3 (8.6, 14.8) | -0.1 (-0.3, 0.2) |
| Seychelles | 2 (2, 3) | 2 (1.7, 2.4) | 0.2 (0, 0.5) | 2 (2, 2) | 1.7 (1.5, 2.1) | 0.1 (-0.1, 0.4) | 65 (54, 79) | 53 (44.4, 63.7) | 0.1 (-0.2, 0.3) |
| Sierra Leone | 14 (10, 19) | 0.3 (0.2, 0.4) | 0.3 (-0.1, 0.8) | 13 (10, 18) | 0.3 (0.2, 0.4) | 0.2 (-0.1, 0.8) | 502 (358, 682) | 9.9 (7.1, 13.4) | 0.3 (-0.1, 0.9) |
| Singapore | 884 (671, 1167) | 10.8 (8.3, 14.2) | -0.2 (-0.4, 0) | 152 (133, 175) | 1.9 (1.6, 2.2) | -0.7 (-0.7, -0.6) | 5063 (4383, 5899) | 61.8 (53.7, 71.8) | -0.7 (-0.7, -0.6) |
| Slovakia | 57 (42, 78) | 0.8 (0.6, 1) | -0.2 (-0.4, 0.1) | 29 (22, 38) | 0.3 (0.3, 0.5) | -0.5 (-0.7, -0.4) | 873 (654, 1164) | 10.9 (8.1, 14.5) | -0.6 (-0.7, -0.4) |
| Slovenia | 14 (10, 18) | 0.4 (0.3, 0.6) | -0.2 (-0.5, 0.2) | 7 (5, 9) | 0.2 (0.1, 0.3) | -0.5 (-0.7, -0.3) | 197 (147, 261) | 6 (4.4, 8) | -0.5 (-0.7, -0.3) |
| Solomon Islands | 8 (4, 13) | 2.1 (1.2, 3.2) | 0 (-0.3, 0.4) | 8 (4, 12) | 2 (1.2, 3) | 0 (-0.3, 0.3) | 287 (147, 453) | 65.3 (35.8, 100.7) | 0 (-0.3, 0.4) |
| Somalia | 123 (83, 175) | 1.4 (0.9, 1.9) | -0.2 (-0.4, 0.2) | 119 (82, 170) | 1.4 (1, 1.9) | -0.1 (-0.4, 0.2) | 4651 (3143, 6628) | 44.9 (30.7, 64.4) | -0.1 (-0.4, 0.2) |
| South Africa | 209 (188, 233) | 0.4 (0.4, 0.5) | -0.2 (-0.3, -0.1) | 200 (180, 224) | 0.4 (0.4, 0.5) | -0.2 (-0.3, -0.1) | 5984 (5357, 6712) | 12 (10.7, 13.4) | -0.3 (-0.3, -0.2) |
| South Sudan | 46 (28, 72) | 0.9 (0.6, 1.5) | -0.2 (-0.5, 0.1) | 44 (26, 70) | 1 (0.6, 1.5) | -0.2 (-0.5, 0.1) | 1611 (950, 2591) | 29.6 (17.6, 47.2) | -0.2 (-0.5, 0.2) |
| Spain | 1170 (867, 1561) | 1.6 (1.2, 2.1) | 0.1 (-0.2, 0.5) | 275 (243, 306) | 0.3 (0.3, 0.4) | -0.5 (-0.5, -0.4) | 7806 (6810, 8795) | 10.7 (9.3, 12) | -0.5 (-0.5, -0.4) |
| Sri Lanka | 252 (185, 338) | 1 (0.7, 1.3) | 0 (-0.3, 0.3) | 207 (152, 277) | 0.8 (0.6, 1.1) | -0.2 (-0.5, 0.1) | 5633 (4080, 7633) | 21.7 (15.8, 29.2) | -0.2 (-0.4, 0.1) |
| Sudan | 102 (68, 144) | 0.4 (0.3, 0.6) | -0.2 (-0.5, 0.2) | 71 (50, 97) | 0.3 (0.2, 0.4) | -0.3 (-0.5, 0) | 2569 (1754, 3608) | 9.9 (6.9, 13.7) | -0.4 (-0.6, 0) |
| Suriname | 6 (5, 7) | 0.9 (0.7, 1.1) | 0.3 (0.1, 0.7) | 5 (4, 7) | 0.9 (0.7, 1.1) | 0.3 (0, 0.6) | 161 (129, 199) | 25.6 (20.6, 31.4) | 0.3 (0, 0.6) |
| Sweden | 69 (57, 83) | 0.5 (0.4, 0.6) | 0 (-0.2, 0.2) | 18 (16, 20) | 0.1 (0.1, 0.1) | -0.4 (-0.4, -0.3) | 490 (444, 539) | 3.3 (3, 3.6) | -0.3 (-0.4, -0.3) |
| Switzerland | 162 (119, 215) | 1.1 (0.8, 1.5) | 0.5 (0.1, 1) | 37 (32, 42) | 0.2 (0.2, 0.3) | 0 (-0.2, 0.1) | 1015 (870, 1177) | 7.3 (6.3, 8.4) | -0.1 (-0.2, 0.1) |
| Syrian Arab Republic | 37 (28, 51) | 0.3 (0.2, 0.4) | 0.6 (0.1, 1.4) | 15 (11, 20) | 0.1 (0.1, 0.2) | -0.2 (-0.4, 0.2) | 543 (405, 729) | 3.7 (2.8, 5) | -0.2 (-0.5, 0.2) |
| Taiwan (Province of China) | 2380 (1769, 3212) | 7.1 (5.3, 9.7) | 0 (-0.2, 0.4) | 919 (691, 1211) | 2.4 (1.9, 3.2) | -0.5 (-0.6, -0.3) | 29183 (21981, 38765) | 81.5 (61.6, 108.9) | -0.5 (-0.6, -0.3) |
| Tajikistan | 32 (25, 40) | 0.6 (0.4, 0.7) | 0 (-0.3, 0.3) | 31 (24, 39) | 0.6 (0.5, 0.7) | 0 (-0.3, 0.4) | 1022 (792, 1283) | 15.8 (12.4, 19.6) | -0.1 (-0.3, 0.2) |
| Thailand | 1539 (1094, 2040) | 1.6 (1.1, 2.1) | 0 (-0.3, 0.4) | 1176 (848, 1568) | 1.2 (0.8, 1.5) | -0.2 (-0.5, 0) | 35203 (25311, 47160) | 35.6 (25.7, 47.3) | -0.2 (-0.5, 0) |
| The Republic of Côte d'Ivoire | 49 (34, 67) | 0.3 (0.2, 0.4) | 0.2 (-0.2, 0.7) | 46 (32, 62) | 0.3 (0.2, 0.4) | 0.2 (-0.2, 0.6) | 1778 (1231, 2444) | 10.8 (7.5, 14.5) | 0.2 (-0.2, 0.7) |
| Timor-Leste | 13 (9, 18) | 1.5 (1, 2) | -0.1 (-0.4, 0.3) | 13 (9, 17) | 1.5 (1.1, 2.1) | -0.1 (-0.3, 0.3) | 392 (244, 535) | 42.7 (27.8, 58.5) | -0.1 (-0.4, 0.3) |
| Togo | 16 (11, 22) | 0.3 (0.2, 0.4) | 0.3 (-0.1, 0.8) | 15 (11, 20) | 0.3 (0.2, 0.4) | 0.3 (-0.1, 0.8) | 571 (398, 772) | 10.6 (7.6, 14.3) | 0.3 (-0.1, 0.9) |
| Tokelau | 0 (0, 0) | 1 (0.7, 1.4) | -0.1 (-0.4, 0.2) | 0 (0, 0) | 0.9 (0.7, 1.2) | -0.2 (-0.4, 0.1) | 0 (0, 1) | 28.2 (20.4, 38.6) | -0.2 (-0.4, 0.1) |
| Tonga | 1 (1, 1) | 1 (0.7, 1.3) | 0 (-0.3, 0.4) | 1 (1, 1) | 0.9 (0.7, 1.3) | 0 (-0.3, 0.4) | 23 (17, 32) | 27.8 (20.3, 38.4) | 0 (-0.3, 0.4) |
| Trinidad and Tobago | 7 (5, 9) | 0.4 (0.3, 0.5) | 0.2 (-0.1, 0.6) | 6 (4, 8) | 0.3 (0.2, 0.4) | 0.1 (-0.2, 0.4) | 180 (132, 240) | 10.6 (7.7, 14.2) | 0.1 (-0.2, 0.5) |
| Tunisia | 652 (454, 900) | 4.9 (3.5, 6.8) | 0.6 (0.1, 1.4) | 226 (162, 312) | 1.7 (1.3, 2.4) | -0.3 (-0.5, 0.1) | 7393 (5290, 10222) | 55.6 (40, 76.7) | -0.2 (-0.5, 0.1) |
| Turkey | 1069 (831, 1354) | 1.2 (0.9, 1.5) | 0.2 (-0.2, 0.6) | 414 (327, 515) | 0.5 (0.4, 0.6) | -0.5 (-0.6, -0.3) | 13500 (10611, 16834) | 14.6 (11.5, 18.2) | -0.5 (-0.6, -0.3) |
| Turkmenistan | 21 (17, 26) | 0.4 (0.4, 0.6) | 0.5 (0.2, 0.9) | 17 (13, 21) | 0.4 (0.3, 0.5) | 0.4 (0.1, 0.7) | 705 (571, 872) | 14.6 (11.8, 17.9) | 0.4 (0.1, 0.7) |
| Tuvalu | 0 (0, 0) | 1.5 (1, 2.3) | -0.2 (-0.4, 0.2) | 0 (0, 0) | 1.4 (1, 2.2) | -0.2 (-0.4, 0.1) | 5 (3, 8) | 44.7 (30.2, 70.1) | -0.2 (-0.4, 0.2) |
| Uganda | 397 (299, 518) | 2.1 (1.6, 2.7) | -0.1 (-0.4, 0.2) | 368 (277, 476) | 2.1 (1.6, 2.6) | -0.2 (-0.4, 0.1) | 14225 (10566, 18969) | 66.7 (49.9, 86.8) | -0.1 (-0.4, 0.2) |
| Ukraine | 663 (543, 796) | 1.1 (0.9, 1.4) | 1.2 (0.7, 1.8) | 322 (261, 397) | 0.5 (0.4, 0.6) | 0.7 (0.2, 1.2) | 10918 (8817, 13457) | 18.2 (14.8, 22.3) | 0.7 (0.3, 1.3) |
| United Arab Emirates | 58 (37, 87) | 0.6 (0.4, 0.8) | -0.2 (-0.4, 0.2) | 33 (22, 53) | 0.4 (0.3, 0.6) | -0.4 (-0.6, 0) | 1435 (910, 2343) | 13.3 (9.1, 19.8) | -0.3 (-0.5, 0) |
| United Kingdom | 1121 (928, 1344) | 1.1 (0.9, 1.4) | 0 (-0.2, 0.2) | 277 (263, 291) | 0.3 (0.2, 0.3) | -0.3 (-0.3, -0.2) | 7667 (7276, 8061) | 8 (7.6, 8.4) | -0.2 (-0.3, -0.2) |
| United Republic of Tanzania | 381 (253, 548) | 1.3 (0.8, 1.8) | -0.2 (-0.4, 0.1) | 360 (239, 513) | 1.2 (0.8, 1.7) | -0.2 (-0.4, 0.1) | 13157 (8646, 19258) | 39.1 (26, 56.3) | -0.2 (-0.4, 0.2) |
| United States of America | 2870 (2431, 3378) | 0.6 (0.5, 0.7) | -0.1 (-0.3, 0) | 1127 (1073, 1180) | 0.2 (0.2, 0.2) | -0.3 (-0.4, -0.3) | 32364 (30958, 33905) | 7 (6.7, 7.3) | -0.3 (-0.4, -0.3) |
| United States Virgin Islands | 1 (1, 2) | 0.9 (0.7, 1.1) | 0.1 (-0.1, 0.5) | 1 (1, 2) | 0.8 (0.6, 0.9) | 0 (-0.2, 0.3) | 33 (26, 39) | 20.1 (16.1, 24.5) | 0 (-0.2, 0.3) |
| Uruguay | 25 (19, 32) | 0.5 (0.4, 0.7) | -0.1 (-0.4, 0.2) | 16 (14, 19) | 0.3 (0.3, 0.4) | -0.3 (-0.4, -0.2) | 457 (403, 516) | 10.5 (9.3, 11.9) | -0.3 (-0.4, -0.2) |
| Uzbekistan | 171 (138, 205) | 0.6 (0.5, 0.7) | 0.7 (0.2, 1.3) | 142 (115, 170) | 0.6 (0.5, 0.7) | 0.6 (0.2, 1.2) | 5582 (4570, 6699) | 18.5 (15.1, 22.1) | 0.5 (0.1, 1.1) |
| Vanuatu | 3 (2, 4) | 1.5 (1, 2.1) | 0 (-0.3, 0.5) | 3 (2, 4) | 1.5 (1, 2.1) | 0 (-0.3, 0.5) | 95 (64, 136) | 46.1 (31.6, 65.8) | 0.1 (-0.3, 0.6) |
| Venezuela (Bolivarian Republic of) | 108 (80, 142) | 0.4 (0.3, 0.5) | 0.6 (0.1, 1.1) | 88 (65, 115) | 0.3 (0.2, 0.4) | 0.3 (0, 0.7) | 2571 (1883, 3348) | 8.6 (6.3, 11.1) | 0.3 (0, 0.8) |
| Viet Nam | 3460 (2614, 4462) | 3.3 (2.5, 4.2) | 0 (-0.3, 0.3) | 2788 (2136, 3586) | 2.7 (2.1, 3.5) | -0.2 (-0.4, 0.1) | 93450 (70634, 121735) | 86.4 (65.7, 111.1) | -0.2 (-0.4, 0.1) |
| Yemen | 77 (54, 109) | 0.5 (0.3, 0.6) | -0.2 (-0.4, 0.2) | 60 (44, 83) | 0.4 (0.3, 0.5) | -0.2 (-0.5, 0.1) | 2177 (1509, 3063) | 11.7 (8.4, 16.5) | -0.3 (-0.5, 0.1) |
| Zambia | 127 (90, 172) | 1.4 (1, 1.9) | -0.2 (-0.4, 0.1) | 118 (84, 159) | 1.4 (1, 1.8) | -0.2 (-0.4, 0.1) | 4553 (3199, 6191) | 44.5 (31.8, 60.3) | -0.2 (-0.4, 0.1) |
| Zimbabwe | 63 (48, 83) | 0.7 (0.5, 0.9) | 0.2 (-0.1, 0.5) | 59 (44, 76) | 0.7 (0.5, 0.9) | 0.2 (-0.1, 0.5) | 2191 (1638, 2911) | 22.6 (16.9, 29.6) | 0.2 (-0.1, 0.6) |

DALYs, disability adjusted life years; UI, uncertainty interval.

Supplementary Table 8 The prevalence, YLLs and YLDs for NPC for both sex in 2019, and percentage change of age-standardized rates (ASRs) by 204 countries, 1990-2019.

|  | Prevalence (95% Uncertainty Interval) | | | YLLs (95% Uncertainty Interval) | | | YLDs (95% Uncertainty Interval) | | |
| --- | --- | --- | --- | --- | --- | --- | --- | --- | --- |
|  | Counts | ASR per 100,000 population (95%UI) | Percentage change in ASRs per 100,000 population (95%UI) | Counts | ASR per 100,000 population (95%UI) | Percentage change in ASRs per 100,000 population(95%UI) | Numbers | ASR per 100,000 population (95%UI) | Percentage change in ASRs per 100,000 population (95%UI) |
| Afghanistan | 358 (206, 536) | 1.7 (1.1, 2.5) | -0.2 (-0.5, 0.1) | 3825 (2295, 5761) | 18.4 (11.4, 26.8) | -0.3 (-0.5, -0.1) | 45 (24, 72) | 0.2 (0.1, 0.4) | -0.3 (-0.5, 0) |
| Albania | 58 (42, 79) | 1.7 (1.2, 2.4) | 0.1 (-0.2, 0.6) | 333 (248, 439) | 18.4 (11.4, 26.8) | -0.4 (-0.6, -0.2) | 8 (5, 12) | 0.2 (0.1, 0.3) | -0.1 (-0.3, 0.2) |
| Algeria | 9463 (6974, 12811) | 22.5 (16.8, 30.2) | 0.6 (0.1, 1.3) | 22383 (17331, 28343) | 55.8 (43.1, 70.8) | -0.5 (-0.6, -0.3) | 893 (569, 1347) | 2.2 (1.4, 3.3) | 0.3 (-0.1, 0.8) |
| American Samoa | 3 (2, 3) | 4.7 (3.8, 5.9) | 0.2 (-0.1, 0.6) | 25 (20, 31) | 46.4 (37.5, 57.4) | 0.1 (-0.1, 0.5) | 0 (0, 0) | 0.7 (0.5, 0.9) | 0.1 (-0.1, 0.5) |
| Andorra | 12 (9, 17) | 9.3 (6.7, 12.8) | 0.4 (-0.1, 1.3) | 11 (8, 15) | 8.5 (6.3, 11.3) | -0.3 (-0.6, 0) | 1 (1, 2) | 0.9 (0.6, 1.4) | 0.3 (-0.2, 1.1) |
| Angola | 217 (155, 279) | 1.4 (1, 1.8) | -0.1 (-0.4, 0.3) | 2562 (1814, 3307) | 15.9 (11.6, 20.7) | -0.2 (-0.4, 0.2) | 30 (19, 44) | 0.2 (0.1, 0.3) | -0.1 (-0.4, 0.3) |
| Antigua and Barbuda | 2 (1, 2) | 1.5 (1.2, 1.9) | 0.5 (0.2, 0.9) | 10 (9, 13) | 10 (8.2, 12) | 0.1 (-0.1, 0.4) | 0 (0, 0) | 0.2 (0.1, 0.3) | 0.4 (0.2, 0.8) |
| Argentina | 582 (431, 753) | 1.2 (0.9, 1.5) | -0.1 (-0.3, 0.2) | 2991 (2694, 3325) | 6 (5.4, 6.6) | -0.5 (-0.5, -0.4) | 72 (47, 105) | 0.1 (0.1, 0.2) | -0.2 (-0.4, 0) |
| Armenia | 38 (31, 47) | 1.1 (0.9, 1.4) | 0.3 (-0.1, 0.7) | 272 (227, 325) | 7.6 (6.3, 9) | -0.2 (-0.4, 0) | 5 (3, 6) | 0.1 (0.1, 0.2) | 0.1 (-0.1, 0.4) |
| Australia | 1405 (1070, 1805) | 4.2 (3.2, 5.5) | -0.2 (-0.4, 0.1) | 3007 (2680, 3344) | 8.9 (8, 10) | -0.4 (-0.5, -0.3) | 149 (96, 215) | 0.4 (0.3, 0.6) | -0.2 (-0.4, 0.1) |
| Austria | 496 (370, 656) | 3.7 (2.8, 4.8) | 0.2 (-0.2, 0.6) | 733 (649, 832) | 5.3 (4.7, 5.9) | -0.5 (-0.6, -0.4) | 53 (33, 78) | 0.4 (0.2, 0.5) | 0 (-0.2, 0.4) |
| Azerbaijan | 71 (57, 89) | 0.7 (0.5, 0.8) | 0.5 (0.1, 0.9) | 668 (537, 827) | 6.1 (4.9, 7.4) | 0.2 (-0.1, 0.6) | 9 (6, 12) | 0.1 (0.1, 0.1) | 0.4 (0.1, 0.8) |
| Bahamas | 8 (7, 11) | 2 (1.6, 2.5) | 0.3 (0, 0.6) | 67 (53, 84) | 15.6 (12.4, 19.5) | 0.1 (-0.2, 0.3) | 1 (1, 2) | 0.3 (0.2, 0.4) | 0.2 (-0.1, 0.5) |
| Bahrain | 49 (37, 65) | 3.1 (2.5, 4) | 0.5 (0.1, 1) | 132 (98, 174) | 8.7 (6.6, 11.5) | -0.5 (-0.7, -0.4) | 5 (3, 7) | 0.3 (0.2, 0.5) | 0.1 (-0.2, 0.5) |
| Bangladesh | 3905 (2752, 5489) | 2.7 (1.9, 3.8) | -0.3 (-0.6, 0) | 40394 (28634, 57411) | 27.5 (19.5, 39) | -0.4 (-0.6, -0.1) | 526 (321, 819) | 0.4 (0.2, 0.6) | -0.4 (-0.6, 0) |
| Barbados | 11 (9, 14) | 2.7 (2.1, 3.4) | 0.8 (0.3, 1.2) | 72 (58, 89) | 16.3 (13.2, 20.1) | 0.3 (0, 0.6) | 2 (1, 2) | 0.3 (0.2, 0.5) | 0.6 (0.3, 1) |
| Belarus | 275 (193, 381) | 2.2 (1.5, 3) | 0.8 (0.3, 1.5) | 1475 (1063, 1977) | 10.6 (7.7, 14.1) | -0.1 (-0.3, 0.3) | 33 (20, 50) | 0.2 (0.2, 0.4) | 0.5 (0.1, 1.1) |
| Belgium | 1346 (996, 1814) | 8 (5.9, 10.8) | 0.6 (0.2, 1.3) | 1397 (1242, 1576) | 8.1 (7.2, 9.1) | -0.4 (-0.4, -0.3) | 137 (87, 208) | 0.8 (0.5, 1.2) | 0.5 (0, 1.2) |
| Belize | 4 (3, 5) | 1.2 (1, 1.4) | 0.9 (0.5, 1.3) | 36 (30, 42) | 10.6 (8.9, 12.4) | 0.7 (0.4, 1) | 1 (0, 1) | 0.2 (0.1, 0.2) | 0.8 (0.5, 1.2) |
| Benin | 55 (38, 77) | 0.8 (0.6, 1.1) | 0.2 (-0.1, 0.7) | 658 (450, 930) | 9.3 (6.5, 13.1) | 0.2 (-0.2, 0.7) | 7 (4, 11) | 0.1 (0.1, 0.2) | 0.2 (-0.1, 0.7) |
| Bermuda | 5 (4, 6) | 4.8 (3.7, 6.1) | 1.2 (0.7, 1.9) | 13 (10, 16) | 11.5 (9.2, 14.2) | -0.2 (-0.4, 0) | 1 (0, 1) | 0.5 (0.3, 0.7) | 0.8 (0.4, 1.4) |
| Bhutan | 17 (11, 24) | 2.6 (1.8, 3.6) | -0.2 (-0.5, 0.2) | 168 (110, 237) | 25.7 (17.2, 35.9) | -0.4 (-0.6, 0) | 2 (1, 4) | 0.4 (0.2, 0.6) | -0.3 (-0.5, 0.1) |
| Bolivia (Plurinational State of) | 65 (48, 84) | 0.7 (0.5, 0.9) | 0 (-0.3, 0.3) | 648 (482, 851) | 6.6 (5, 8.7) | -0.2 (-0.4, 0.1) | 9 (6, 13) | 0.1 (0.1, 0.1) | -0.1 (-0.3, 0.3) |
| Bosnia and Herzegovina | 39 (31, 51) | 0.8 (0.6, 1) | 1.4 (0.8, 2.2) | 281 (217, 358) | 5.3 (4.1, 6.8) | 0.5 (0.2, 1) | 6 (4, 8) | 0.1 (0.1, 0.2) | 1.2 (0.7, 1.9) |
| Botswana | 22 (15, 31) | 1.2 (0.9, 1.7) | 0.1 (-0.3, 0.7) | 215 (143, 303) | 11.9 (8.1, 16.5) | -0.1 (-0.4, 0.4) | 3 (2, 4) | 0.2 (0.1, 0.3) | 0 (-0.3, 0.5) |
| Brazil | 2493 (2322, 2681) | 1 (1, 1.1) | 0.5 (0.4, 0.7) | 17950 (16844, 19048) | 7.4 (7, 7.9) | 0 (-0.1, 0.1) | 299 (216, 396) | 0.1 (0.1, 0.2) | 0.4 (0.2, 0.5) |
| Brunei Darussalam | 113 (87, 145) | 25.2 (20, 31.6) | 0.5 (0.1, 1) | 379 (319, 450) | 87.2 (74.2, 102.7) | -0.2 (-0.4, 0) | 11 (8, 16) | 2.7 (1.8, 3.8) | 0.3 (-0.1, 0.8) |
| Bulgaria | 326 (230, 456) | 3.7 (2.6, 5.2) | 1.7 (0.7, 3) | 1244 (937, 1623) | 11.7 (8.8, 15.2) | 0.7 (0.3, 1.2) | 36 (22, 55) | 0.4 (0.2, 0.6) | 1.4 (0.6, 2.7) |
| Burkina Faso | 111 (80, 149) | 0.9 (0.6, 1.2) | 0.4 (0, 0.9) | 1341 (962, 1817) | 10.3 (7.5, 13.6) | 0.3 (0, 0.9) | 15 (9, 22) | 0.1 (0.1, 0.2) | 0.3 (0, 0.8) |
| Burundi | 243 (165, 336) | 3.8 (2.5, 5.2) | -0.2 (-0.5, 0.2) | 2986 (2025, 4111) | 44.4 (30.2, 61.8) | -0.3 (-0.5, 0.1) | 33 (20, 52) | 0.6 (0.3, 0.9) | -0.3 (-0.5, 0.1) |
| Cabo Verde | 6 (4, 8) | 1.1 (0.9, 1.4) | 2.6 (1.7, 3.7) | 53 (41, 68) | 10.3 (8.1, 13.2) | 2 (1.2, 2.8) | 1 (0, 1) | 0.1 (0.1, 0.2) | 2.4 (1.6, 3.3) |
| Cambodia | 738 (548, 955) | 5.3 (4, 6.9) | -0.1 (-0.3, 0.3) | 7836 (5821, 10157) | 56.4 (41.9, 72.8) | -0.2 (-0.4, 0.1) | 103 (67, 150) | 0.8 (0.5, 1.1) | -0.1 (-0.4, 0.2) |
| Cameroon | 465 (305, 661) | 2.7 (1.8, 3.8) | 0 (-0.3, 0.4) | 5537 (3599, 7989) | 30.7 (20.8, 44.1) | 0 (-0.3, 0.4) | 62 (36, 97) | 0.4 (0.2, 0.6) | 0 (-0.3, 0.4) |
| Canada | 4216 (3074, 5587) | 8 (5.9, 10.5) | 0.2 (-0.2, 0.6) | 3672 (3246, 4121) | 6.8 (6, 7.6) | -0.3 (-0.4, -0.2) | 416 (260, 620) | 0.8 (0.5, 1.1) | 0.1 (-0.2, 0.6) |
| Central African Republic | 50 (34, 72) | 1.7 (1.2, 2.3) | -0.2 (-0.4, 0.1) | 641 (435, 922) | 20.6 (14.5, 28.5) | -0.2 (-0.4, 0.1) | 7 (4, 11) | 0.3 (0.2, 0.4) | -0.2 (-0.4, 0.1) |
| Chad | 68 (50, 90) | 0.9 (0.7, 1.1) | 0.5 (0.1, 1.1) | 842 (609, 1107) | 10.3 (7.6, 13.5) | 0.5 (0.1, 1.1) | 9 (6, 13) | 0.1 (0.1, 0.2) | 0.5 (0.1, 1) |
| Chile | 274 (204, 374) | 1.3 (0.9, 1.7) | 0.9 (0.4, 1.7) | 716 (635, 813) | 3.2 (2.8, 3.6) | -0.3 (-0.4, -0.2) | 31 (20, 45) | 0.1 (0.1, 0.2) | 0.6 (0.2, 1.1) |
| China | 688195 (561636, 830005) | 35.4 (29.1, 42.4) | 2.3 (1.6, 3.3) | 847682 (699217, #####) | 42.3 (35.1, 50.8) | -0.5 (-0.6, -0.4) | 64425 (43940, 88926) | 3.3 (2.3, 4.6) | 1.4 (0.9, 2.1) |
| Colombia | 457 (338, 613) | 0.9 (0.7, 1.2) | 0 (-0.3, 0.3) | 2425 (1811, 3186) | 4.7 (3.5, 6.1) | -0.5 (-0.6, -0.3) | 56 (35, 82) | 0.1 (0.1, 0.2) | -0.2 (-0.4, 0.1) |
| Comoros | 17 (12, 26) | 3.1 (2.1, 4.4) | -0.1 (-0.4, 1.1) | 204 (137, 301) | 35.2 (24.2, 51.1) | -0.1 (-0.4, 1) | 2 (1, 4) | 0.4 (0.3, 0.7) | -0.1 (-0.4, 0.9) |
| Congo | 49 (36, 68) | 1.5 (1.1, 2) | -0.3 (-0.5, 0.1) | 581 (418, 805) | 16.4 (11.9, 22.6) | -0.3 (-0.6, 0) | 7 (4, 11) | 0.2 (0.1, 0.3) | -0.3 (-0.5, 0.1) |
| Cook Islands | 0 (0, 0) | 1.1 (0.8, 1.6) | 0 (-0.4, 0.5) | 1 (1, 2) | 6.2 (4.8, 7.9) | -0.4 (-0.5, -0.1) | 0 (0, 0) | 0.1 (0.1, 0.2) | -0.1 (-0.4, 0.2) |
| Costa Rica | 178 (124, 257) | 3.6 (2.5, 5.2) | 0.9 (0.3, 1.8) | 658 (494, 864) | 13 (9.7, 17) | 0 (-0.3, 0.3) | 19 (12, 28) | 0.4 (0.2, 0.6) | 0.6 (0.1, 1.3) |
| Croatia | 190 (135, 258) | 3.2 (2.3, 4.4) | 0.1 (-0.2, 0.6) | 472 (354, 625) | 7.1 (5.3, 9.4) | -0.5 (-0.7, -0.4) | 21 (13, 31) | 0.3 (0.2, 0.5) | 0 (-0.3, 0.4) |
| Cuba | 529 (404, 682) | 3.2 (2.5, 4.1) | 0.9 (0.4, 1.6) | 2485 (1949, 3174) | 14.2 (11.2, 17.9) | 0.2 (-0.1, 0.6) | 66 (44, 95) | 0.4 (0.3, 0.6) | 0.7 (0.3, 1.3) |
| Cyprus | 79 (63, 97) | 4.6 (3.7, 5.6) | 3 (2, 4.2) | 82 (69, 96) | 4.7 (4, 5.5) | -0.1 (-0.3, 0.1) | 8 (5, 11) | 0.5 (0.3, 0.6) | 2.3 (1.5, 3.3) |
| Czechia | 284 (214, 374) | 2 (1.5, 2.6) | 0.3 (-0.1, 0.7) | 1046 (818, 1323) | 6.4 (5, 8.2) | -0.5 (-0.6, -0.4) | 32 (21, 47) | 0.2 (0.1, 0.3) | 0 (-0.3, 0.3) |
| Democratic People's Republic of Korea | 1806 (1277, 2443) | 5.5 (3.9, 7.5) | -0.2 (-0.5, 0.1) | 16802 (11921, 22487) | 50.7 (36.2, 67.7) | -0.2 (-0.5, 0.1) | 239 (152, 354) | 0.7 (0.5, 1.1) | -0.2 (-0.4, 0.1) |
| Democratic Republic of the Congo | 589 (435, 771) | 1.2 (0.9, 1.6) | -0.1 (-0.4, 0.2) | 7002 (5200, 9140) | 14.3 (10.5, 18.8) | -0.2 (-0.4, 0.2) | 83 (53, 124) | 0.2 (0.1, 0.3) | -0.1 (-0.4, 0.2) |
| Denmark | 335 (245, 450) | 4 (3, 5.4) | 1 (0.4, 1.8) | 349 (302, 402) | 4 (3.5, 4.6) | -0.2 (-0.3, -0.1) | 35 (21, 51) | 0.4 (0.2, 0.6) | 0.9 (0.3, 1.6) |
| Djibouti | 30 (19, 45) | 3.5 (2.3, 5.1) | -0.1 (-0.4, 0.4) | 351 (217, 534) | 39.7 (25.6, 58.5) | -0.1 (-0.4, 0.3) | 4 (2, 6) | 0.5 (0.3, 0.8) | -0.1 (-0.4, 0.4) |
| Dominica | 2 (1, 2) | 1.8 (1.4, 2.3) | 0.4 (0, 0.8) | 14 (11, 18) | 16.5 (13.1, 21.2) | 0.4 (0, 0.8) | 0 (0, 0) | 0.3 (0.2, 0.4) | 0.4 (0.1, 0.8) |
| Dominican Republic | 126 (92, 167) | 1.3 (0.9, 1.7) | 0.5 (0, 1) | 1096 (797, 1453) | 11.2 (8.2, 14.7) | 0.2 (-0.1, 0.7) | 18 (11, 27) | 0.2 (0.1, 0.3) | 0.4 (0, 0.9) |
| Ecuador | 96 (74, 128) | 0.6 (0.5, 0.8) | 0.3 (0, 0.8) | 720 (555, 940) | 4.5 (3.5, 5.8) | -0.1 (-0.3, 0.2) | 13 (8, 18) | 0.1 (0.1, 0.1) | 0.3 (0, 0.7) |
| Egypt | 714 (481, 989) | 0.8 (0.6, 1.1) | 0.9 (0.3, 1.8) | 3037 (2186, 4065) | 3.6 (2.6, 4.9) | -0.1 (-0.4, 0.2) | 74 (45, 113) | 0.1 (0.1, 0.1) | 0.6 (0.1, 1.2) |
| El Salvador | 45 (34, 60) | 0.7 (0.6, 1) | 0.3 (0, 0.8) | 324 (243, 424) | 5.5 (4.1, 7.1) | -0.1 (-0.3, 0.2) | 6 (4, 9) | 0.1 (0.1, 0.1) | 0.2 (-0.1, 0.6) |
| Equatorial Guinea | 8 (4, 13) | 1.1 (0.7, 1.9) | -0.3 (-0.6, 0.3) | 86 (48, 143) | 12.3 (7.1, 20.3) | -0.4 (-0.7, 0.1) | 1 (1, 2) | 0.2 (0.1, 0.3) | -0.3 (-0.6, 0.2) |
| Eritrea | 151 (98, 213) | 3.8 (2.5, 5.3) | 0 (-0.3, 0.5) | 1883 (1214, 2669) | 45.6 (29.8, 63.6) | -0.1 (-0.4, 0.3) | 21 (12, 32) | 0.6 (0.3, 0.9) | -0.1 (-0.4, 0.4) |
| Estonia | 39 (27, 54) | 2.3 (1.6, 3.3) | 0 (-0.3, 0.5) | 161 (118, 216) | 8.6 (6.2, 11.3) | -0.6 (-0.7, -0.4) | 4 (3, 7) | 0.2 (0.1, 0.4) | -0.2 (-0.4, 0.2) |
| Eswatini | 13 (9, 19) | 1.8 (1.3, 2.6) | 0 (-0.3, 0.5) | 153 (104, 218) | 20.8 (14.4, 29.4) | 0 (-0.3, 0.5) | 2 (1, 3) | 0.3 (0.2, 0.4) | 0 (-0.3, 0.5) |
| Ethiopia | 1550 (991, 2035) | 2.8 (1.8, 3.7) | -0.3 (-0.5, 0.2) | 18237 (11506, 24266) | 31.4 (19.7, 41.6) | -0.4 (-0.6, 0.1) | 210 (118, 318) | 0.4 (0.2, 0.6) | -0.4 (-0.5, 0.1) |
| Fiji | 9 (7, 12) | 1 (0.8, 1.3) | -0.1 (-0.4, 0.2) | 98 (74, 128) | 11.1 (8.5, 14.4) | -0.2 (-0.4, 0.2) | 1 (1, 2) | 0.2 (0.1, 0.2) | -0.1 (-0.4, 0.2) |
| Finland | 253 (188, 340) | 3.1 (2.3, 4.1) | 0.7 (0.2, 1.4) | 251 (218, 289) | 2.9 (2.6, 3.4) | -0.4 (-0.5, -0.2) | 26 (17, 40) | 0.3 (0.2, 0.5) | 0.6 (0.2, 1.2) |
| France | 9869 (6953, 13564) | 10.2 (7.2, 14.1) | 0.1 (-0.3, 0.6) | 10081 (8809, 11534) | 10.2 (8.9, 11.7) | -0.7 (-0.7, -0.6) | 1002 (627, 1501) | 1 (0.6, 1.5) | 0 (-0.3, 0.4) |
| Gabon | 19 (13, 26) | 1.5 (1, 2) | -0.2 (-0.5, 0.2) | 209 (146, 288) | 16.1 (11.2, 22.2) | -0.3 (-0.5, 0.1) | 3 (2, 4) | 0.2 (0.1, 0.3) | -0.2 (-0.5, 0.2) |
| Gambia | 8 (6, 11) | 0.6 (0.5, 0.9) | 0.2 (-0.2, 0.8) | 97 (69, 132) | 7.4 (5.3, 10) | 0.2 (-0.2, 0.8) | 1 (1, 2) | 0.1 (0.1, 0.1) | 0.2 (-0.2, 0.8) |
| Georgia | 50 (40, 60) | 1 (0.8, 1.3) | -0.1 (-0.3, 0.2) | 450 (369, 544) | 9.1 (7.5, 11) | -0.2 (-0.4, 0.1) | 7 (5, 10) | 0.1 (0.1, 0.2) | -0.1 (-0.3, 0.1) |
| Germany | 7573 (5493, 10260) | 5.6 (4.1, 7.6) | 0.3 (-0.1, 0.8) | 6972 (6134, 7830) | 5 (4.5, 5.6) | -0.5 (-0.6, -0.4) | 785 (494, 1182) | 0.5 (0.3, 0.8) | 0.2 (-0.2, 0.7) |
| Ghana | 97 (71, 128) | 0.4 (0.3, 0.6) | -0.4 (-0.6, -0.1) | 1121 (819, 1474) | 4.9 (3.7, 6.5) | -0.5 (-0.6, -0.2) | 13 (8, 19) | 0.1 (0, 0.1) | -0.4 (-0.6, -0.1) |
| Greece | 1735 (1286, 2329) | 10.9 (8, 14.7) | 1 (0.4, 1.8) | 1966 (1791, 2145) | 12 (11, 13.1) | 0.1 (-0.1, 0.2) | 181 (115, 269) | 1.1 (0.7, 1.6) | 0.9 (0.3, 1.6) |
| Greenland | 16 (13, 20) | 21.6 (17.1, 26.9) | -0.4 (-0.5, -0.2) | 103 (81, 129) | 135.2 (107.3, 168.5) | -0.6 (-0.7, -0.4) | 2 (1, 3) | 2.6 (1.7, 3.6) | -0.4 (-0.6, -0.2) |
| Grenada | 2 (2, 3) | 1.9 (1.6, 2.2) | 0.1 (-0.1, 0.3) | 19 (16, 21) | 15.8 (13.7, 18) | -0.1 (-0.2, 0.1) | 0 (0, 0) | 0.3 (0.2, 0.4) | 0.1 (-0.1, 0.3) |
| Guam | 18 (14, 23) | 9.5 (7.5, 11.8) | 0.1 (-0.2, 0.5) | 149 (118, 184) | 76.3 (61.1, 94) | 0 (-0.2, 0.4) | 2 (2, 3) | 1.2 (0.8, 1.7) | 0.1 (-0.2, 0.5) |
| Guatemala | 91 (70, 116) | 0.7 (0.5, 0.9) | 0.6 (0.2, 1.1) | 872 (676, 1102) | 6.6 (5.1, 8.3) | 0.3 (0, 0.7) | 13 (8, 18) | 0.1 (0.1, 0.1) | 0.5 (0.1, 1) |
| Guinea | 58 (44, 76) | 0.8 (0.6, 1) | 0.1 (-0.2, 0.5) | 709 (533, 937) | 9.5 (7.2, 12.5) | 0.1 (-0.2, 0.5) | 8 (5, 11) | 0.1 (0.1, 0.2) | 0.1 (-0.2, 0.5) |
| Guinea-Bissau | 13 (9, 18) | 1.2 (0.8, 1.7) | 0.2 (-0.2, 0.7) | 156 (108, 219) | 14.3 (9.8, 20) | 0.2 (-0.2, 0.7) | 2 (1, 3) | 0.2 (0.1, 0.3) | 0.2 (-0.2, 0.7) |
| Guyana | 7 (5, 9) | 1 (0.7, 1.3) | 0.3 (-0.1, 0.7) | 74 (54, 97) | 10.1 (7.5, 13.1) | 0.2 (-0.1, 0.6) | 1 (1, 1) | 0.1 (0.1, 0.2) | 0.2 (-0.1, 0.7) |
| Haiti | 172 (105, 240) | 2 (1.2, 2.7) | 0 (-0.3, 0.4) | 2015 (1219, 2811) | 22.3 (13.8, 31) | -0.1 (-0.3, 0.3) | 24 (14, 36) | 0.3 (0.2, 0.5) | 0 (-0.3, 0.3) |
| Honduras | 31 (22, 41) | 0.4 (0.3, 0.6) | 0.1 (-0.2, 0.4) | 304 (218, 403) | 4.3 (3.1, 5.7) | -0.1 (-0.3, 0.2) | 4 (3, 6) | 0.1 (0, 0.1) | 0.1 (-0.2, 0.4) |
| Hungary | 520 (395, 687) | 3.9 (2.9, 5.1) | 1 (0.4, 1.7) | 2559 (1989, 3240) | 17.4 (13.6, 22) | 0 (-0.2, 0.3) | 60 (39, 89) | 0.4 (0.3, 0.6) | 0.7 (0.2, 1.2) |
| Iceland | 25 (20, 30) | 5.4 (4.4, 6.5) | 0.2 (-0.1, 0.5) | 21 (18, 24) | 4.5 (3.8, 5.2) | -0.4 (-0.5, -0.2) | 2 (2, 3) | 0.5 (0.3, 0.7) | 0.1 (-0.2, 0.5) |
| India | 35779 (30441, 42128) | 2.8 (2.4, 3.3) | -0.2 (-0.4, 0) | 383220 (327049, 454497) | 29.4 (25.1, 34.8) | -0.3 (-0.4, -0.1) | 4874 (3445, 6575) | 0.4 (0.3, 0.5) | -0.2 (-0.4, -0.1) |
| Indonesia | 9566 (7478, 12478) | 3.8 (3, 4.9) | 0 (-0.2, 0.2) | 99460 (77024, 130030) | 39.1 (30.4, 50.4) | -0.1 (-0.3, 0.1) | 1337 (889, 1888) | 0.6 (0.4, 0.8) | 0 (-0.2, 0.2) |
| Iran (Islamic Republic of) | 1896 (1687, 2155) | 2.3 (2, 2.6) | 0.8 (0.4, 1.3) | 3919 (3655, 4227) | 4.8 (4.5, 5.1) | -0.4 (-0.5, -0.3) | 191 (134, 258) | 0.2 (0.2, 0.3) | 0.5 (0.3, 0.9) |
| Iraq | 752 (510, 1046) | 2.2 (1.5, 3) | 0.2 (-0.2, 0.7) | 2147 (1559, 2828) | 6.9 (5, 9) | -0.6 (-0.7, -0.4) | 73 (44, 110) | 0.2 (0.1, 0.3) | -0.1 (-0.4, 0.3) |
| Ireland | 373 (272, 501) | 5.7 (4.2, 7.7) | 0.9 (0.4, 1.7) | 329 (283, 384) | 5 (4.3, 5.8) | -0.4 (-0.5, -0.3) | 38 (24, 56) | 0.6 (0.4, 0.8) | 0.7 (0.2, 1.4) |
| Israel | 407 (299, 545) | 4.1 (3, 5.5) | 0.5 (0.1, 1.1) | 535 (475, 603) | 5.3 (4.7, 6) | -0.5 (-0.6, -0.5) | 41 (26, 61) | 0.4 (0.3, 0.6) | 0.3 (0, 0.8) |
| Italy | 9026 (7051, 11339) | 9.3 (7.4, 11.7) | 0.5 (0.1, 1) | 8201 (7689, 8710) | 8.4 (7.9, 8.9) | -0.4 (-0.5, -0.4) | 918 (602, 1299) | 0.9 (0.6, 1.3) | 0.4 (0.1, 0.8) |
| Jamaica | 72 (53, 96) | 2.4 (1.8, 3.2) | 1.3 (0.7, 2.2) | 502 (383, 644) | 16.8 (12.9, 21.5) | 0.9 (0.4, 1.4) | 9 (6, 13) | 0.3 (0.2, 0.4) | 1.1 (0.6, 1.8) |
| Japan | 24147 (19558, 29471) | 9.5 (7.8, 11.6) | 0.4 (0.1, 0.7) | 17674 (16126, 19027) | 7 (6.5, 7.5) | 0 (-0.1, 0) | 2634 (1792, 3710) | 0.9 (0.6, 1.3) | 0.4 (0.1, 0.7) |
| Jordan | 444 (351, 555) | 4.8 (3.9, 6) | 0.3 (0, 0.9) | 1052 (836, 1295) | 12.1 (9.6, 15) | -0.5 (-0.7, -0.4) | 43 (28, 62) | 0.5 (0.3, 0.7) | 0.1 (-0.2, 0.5) |
| Kazakhstan | 306 (249, 379) | 1.6 (1.3, 2) | 0.3 (0.1, 0.6) | 2453 (2025, 2950) | 13.1 (10.9, 15.6) | 0 (-0.2, 0.2) | 39 (27, 55) | 0.2 (0.1, 0.3) | 0.2 (0, 0.5) |
| Kenya | 1427 (946, 1883) | 4.6 (3, 6.1) | 0.1 (-0.1, 0.4) | 17204 (11458, 22796) | 54.4 (36.1, 71.8) | 0.2 (-0.1, 0.4) | 190 (112, 280) | 0.7 (0.4, 1) | 0.1 (-0.1, 0.3) |
| Kiribati | 3 (2, 4) | 3.6 (2.6, 4.9) | -0.1 (-0.3, 0.4) | 40 (29, 54) | 43.8 (31.6, 59) | -0.1 (-0.4, 0.4) | 0 (0, 1) | 0.5 (0.3, 0.8) | -0.1 (-0.3, 0.4) |
| Kuwait | 154 (123, 192) | 3.7 (3.1, 4.6) | -0.4 (-0.5, -0.2) | 251 (199, 313) | 6.6 (5.3, 8.3) | -0.6 (-0.7, -0.5) | 15 (10, 21) | 0.4 (0.3, 0.5) | -0.4 (-0.5, -0.2) |
| Kyrgyzstan | 74 (60, 89) | 1.3 (1.1, 1.6) | 0.3 (0, 0.6) | 685 (569, 812) | 12 (10, 14.2) | 0 (-0.2, 0.3) | 9 (6, 13) | 0.2 (0.1, 0.2) | 0.2 (0, 0.5) |
| Lao People's Democratic Republic | 227 (159, 300) | 4.2 (3, 5.6) | -0.4 (-0.6, -0.1) | 2555 (1774, 3440) | 46.5 (32.9, 61.6) | -0.4 (-0.6, -0.1) | 32 (19, 47) | 0.6 (0.4, 0.9) | -0.4 (-0.6, -0.1) |
| Latvia | 38 (29, 51) | 1.4 (1.1, 1.9) | -0.3 (-0.5, 0) | 241 (183, 314) | 8.3 (6.3, 10.7) | -0.5 (-0.7, -0.4) | 5 (3, 7) | 0.2 (0.1, 0.3) | -0.3 (-0.5, -0.1) |
| Lebanon | 428 (315, 582) | 8.1 (6, 11) | 1.5 (0.7, 2.7) | 700 (536, 930) | 13.4 (10.3, 17.8) | -0.4 (-0.5, -0.1) | 42 (27, 62) | 0.8 (0.5, 1.2) | 1 (0.4, 1.9) |
| Lesotho | 34 (24, 46) | 2.3 (1.6, 3.1) | 0.4 (0, 0.9) | 415 (291, 559) | 27.3 (19.2, 36.5) | 0.4 (0, 1) | 5 (3, 8) | 0.4 (0.2, 0.5) | 0.4 (0, 0.9) |
| Liberia | 24 (17, 34) | 0.8 (0.6, 1.1) | 0.1 (-0.2, 0.7) | 297 (201, 410) | 9.4 (6.5, 13.2) | 0.1 (-0.3, 0.6) | 3 (2, 5) | 0.1 (0.1, 0.2) | 0.1 (-0.2, 0.6) |
| Libya | 1784 (1210, 2530) | 24.5 (17.3, 34.4) | 1 (0.4, 2.2) | 4602 (3488, 6015) | 68.4 (52.6, 88.3) | -0.1 (-0.4, 0.2) | 170 (103, 257) | 2.5 (1.5, 3.7) | 0.7 (0.2, 1.6) |
| Lithuania | 53 (39, 70) | 1.4 (1, 1.9) | -0.3 (-0.5, 0) | 344 (257, 451) | 8.1 (6.1, 10.6) | -0.5 (-0.6, -0.4) | 7 (4, 10) | 0.2 (0.1, 0.2) | -0.4 (-0.5, -0.1) |
| Luxembourg | 86 (65, 112) | 9.9 (7.6, 12.9) | 0.4 (0, 0.9) | 84 (70, 100) | 9.5 (8, 11.4) | -0.5 (-0.6, -0.4) | 9 (5, 13) | 1 (0.6, 1.4) | 0.3 (0, 0.8) |
| Madagascar | 449 (309, 624) | 2.8 (1.9, 3.9) | -0.2 (-0.4, 0.1) | 5526 (3839, 7685) | 33.2 (22.8, 46.2) | -0.2 (-0.4, 0.1) | 61 (37, 92) | 0.4 (0.3, 0.6) | -0.2 (-0.4, 0.1) |
| Malawi | 80 (57, 112) | 0.8 (0.6, 1.1) | -0.2 (-0.4, 0.1) | 977 (683, 1374) | 9.2 (6.6, 12.7) | -0.2 (-0.5, 0.1) | 11 (7, 17) | 0.1 (0.1, 0.2) | -0.2 (-0.4, 0.1) |
| Malaysia | 7140 (5271, 9410) | 22.5 (16.7, 29.5) | 0.1 (-0.2, 0.5) | 46109 (35044, 59370) | 149.7 (113.8, 191.9) | -0.3 (-0.5, -0.1) | 797 (502, 1173) | 2.6 (1.6, 3.8) | -0.1 (-0.3, 0.3) |
| Maldives | 5 (4, 6) | 1 (0.8, 1.2) | -0.1 (-0.4, 0.4) | 21 (17, 26) | 5.2 (4.2, 6.3) | -0.6 (-0.7, -0.3) | 1 (0, 1) | 0.1 (0.1, 0.2) | -0.3 (-0.5, 0.1) |
| Mali | 49 (35, 66) | 0.4 (0.3, 0.5) | 0 (-0.3, 0.4) | 584 (423, 797) | 4.6 (3.4, 6.3) | -0.1 (-0.3, 0.3) | 6 (4, 10) | 0.1 (0, 0.1) | -0.1 (-0.3, 0.3) |
| Malta | 122 (95, 151) | 19.1 (14.8, 23.5) | 0.9 (0.4, 1.5) | 145 (124, 168) | 21.4 (18.3, 24.8) | -0.4 (-0.5, -0.3) | 12 (8, 18) | 1.8 (1.2, 2.6) | 0.7 (0.2, 1.2) |
| Marshall Islands | 2 (1, 3) | 4.7 (3.2, 6.8) | 0 (-0.3, 0.5) | 25 (17, 37) | 54 (36.9, 79.1) | 0 (-0.3, 0.5) | 0 (0, 0) | 0.7 (0.4, 1.1) | 0 (-0.3, 0.5) |
| Mauritania | 17 (10, 26) | 0.6 (0.4, 1) | -0.1 (-0.4, 0.4) | 185 (112, 289) | 7 (4.3, 10.7) | -0.1 (-0.5, 0.3) | 2 (1, 4) | 0.1 (0.1, 0.1) | -0.1 (-0.4, 0.3) |
| Mauritius | 47 (36, 61) | 2.9 (2.3, 3.8) | 0.3 (0, 0.7) | 332 (262, 422) | 19.3 (15.3, 24.5) | -0.1 (-0.3, 0.2) | 6 (4, 9) | 0.4 (0.2, 0.5) | 0.2 (-0.1, 0.5) |
| Mexico | 753 (641, 884) | 0.6 (0.5, 0.7) | 0.2 (0, 0.4) | 5092 (4250, 6012) | 4.1 (3.4, 4.8) | -0.2 (-0.3, 0) | 95 (66, 129) | 0.1 (0.1, 0.1) | 0.1 (-0.1, 0.3) |
| Micronesia (Federated States of) | 4 (2, 6) | 4.4 (2.9, 6.5) | -0.1 (-0.4, 0.3) | 43 (27, 65) | 48.8 (31.1, 71.6) | -0.2 (-0.5, 0.2) | 1 (0, 1) | 0.7 (0.4, 1) | -0.1 (-0.4, 0.3) |
| Monaco | 5 (4, 6) | 7.8 (5.9, 10.1) | 0.4 (-0.1, 1) | 4 (3, 5) | 6.4 (5, 8.3) | -0.1 (-0.4, 0.2) | 0 (0, 1) | 0.7 (0.5, 1.1) | 0.4 (-0.1, 1) |
| Mongolia | 27 (20, 36) | 0.8 (0.6, 1.1) | 0.1 (-0.2, 0.6) | 301 (222, 401) | 9.3 (6.9, 12.2) | 0.1 (-0.3, 0.5) | 4 (2, 5) | 0.1 (0.1, 0.2) | 0.1 (-0.2, 0.6) |
| Montenegro | 7 (5, 9) | 0.9 (0.7, 1.1) | 0.4 (0, 0.9) | 33 (26, 41) | 3.8 (3.1, 4.8) | -0.1 (-0.3, 0.2) | 1 (1, 1) | 0.1 (0.1, 0.1) | 0.3 (0, 0.7) |
| Morocco | 3869 (2774, 5318) | 10.5 (7.6, 14.3) | 0.5 (0, 1.2) | 21936 (15900, 29998) | 60.6 (44.3, 81.7) | -0.2 (-0.4, 0.2) | 434 (272, 645) | 1.2 (0.8, 1.8) | 0.2 (-0.1, 0.8) |
| Mozambique | 59 (44, 76) | 0.4 (0.3, 0.5) | 0.2 (-0.1, 0.7) | 734 (544, 950) | 4.4 (3.3, 5.7) | 0.2 (-0.1, 0.7) | 8 (5, 12) | 0.1 (0, 0.1) | 0.2 (-0.1, 0.6) |
| Myanmar | 2027 (1586, 2594) | 3.9 (3.1, 4.9) | -0.3 (-0.5, 0) | 21815 (17006, 28039) | 41.4 (32.7, 52.7) | -0.4 (-0.6, -0.2) | 285 (191, 410) | 0.6 (0.4, 0.8) | -0.3 (-0.5, -0.1) |
| Namibia | 27 (19, 37) | 1.6 (1.1, 2.1) | 0.1 (-0.3, 0.6) | 295 (209, 410) | 16.8 (12.1, 23) | 0 (-0.3, 0.5) | 4 (2, 5) | 0.2 (0.1, 0.3) | 0 (-0.3, 0.5) |
| Nauru | 0 (0, 0) | 4.8 (3.1, 6.7) | -0.1 (-0.3, 0.2) | 3 (2, 5) | 48.9 (32.2, 69.7) | -0.1 (-0.4, 0.1) | 0 (0, 0) | 0.7 (0.4, 1.1) | -0.1 (-0.3, 0.2) |
| Nepal | 697 (512, 889) | 2.7 (2, 3.5) | -0.2 (-0.5, 0.2) | 7530 (5477, 9652) | 29.5 (21.5, 37.6) | -0.3 (-0.5, 0.1) | 97 (62, 140) | 0.4 (0.3, 0.6) | -0.2 (-0.5, 0.1) |
| Netherlands | 2089 (1530, 2781) | 7.9 (5.8, 10.4) | 0.6 (0.1, 1.1) | 1798 (1574, 2041) | 6.6 (5.9, 7.5) | -0.3 (-0.4, -0.2) | 215 (138, 323) | 0.8 (0.5, 1.2) | 0.5 (0.1, 1) |
| New Zealand | 220 (176, 273) | 3.6 (2.9, 4.5) | 0.1 (-0.2, 0.4) | 554 (507, 607) | 9 (8.3, 9.9) | -0.3 (-0.4, -0.2) | 23 (16, 33) | 0.4 (0.2, 0.5) | 0 (-0.2, 0.3) |
| Nicaragua | 59 (46, 77) | 1.1 (0.9, 1.4) | 0.7 (0.3, 1.1) | 388 (306, 486) | 7.4 (5.9, 9.2) | 0.1 (-0.2, 0.3) | 7 (5, 10) | 0.1 (0.1, 0.2) | 0.5 (0.2, 0.9) |
| Niger | 36 (25, 50) | 0.3 (0.2, 0.4) | -0.1 (-0.4, 0.3) | 438 (304, 608) | 3.5 (2.5, 4.9) | -0.1 (-0.4, 0.2) | 5 (3, 7) | 0 (0, 0.1) | -0.1 (-0.4, 0.2) |
| Nigeria | 2709 (1976, 3590) | 2.3 (1.7, 3) | 0 (-0.3, 0.4) | 31852 (23071, 42160) | 25.9 (19, 33.8) | -0.1 (-0.3, 0.3) | 369 (229, 551) | 0.3 (0.2, 0.5) | 0 (-0.3, 0.4) |
| Niue | 0 (0, 0) | 3.9 (2.8, 5.3) | 0 (-0.3, 0.5) | 1 (0, 1) | 30.2 (21.8, 40) | -0.2 (-0.4, 0.1) | 0 (0, 0) | 0.5 (0.3, 0.8) | 0 (-0.3, 0.3) |
| North Macedonia | 38 (28, 50) | 1.3 (1, 1.8) | 0.3 (0, 0.7) | 240 (185, 311) | 8.1 (6.2, 10.4) | -0.2 (-0.4, 0) | 5 (3, 7) | 0.2 (0.1, 0.2) | 0.1 (-0.1, 0.5) |
| Northern Mariana Islands | 7 (5, 9) | 11 (8.5, 13.8) | 0.3 (-0.1, 0.7) | 48 (38, 59) | 73 (59.5, 88) | 0.1 (-0.2, 0.4) | 1 (1, 1) | 1.3 (0.9, 1.9) | 0.2 (-0.1, 0.6) |
| Norway | 258 (205, 312) | 3.5 (2.8, 4.2) | 0.3 (0, 0.6) | 231 (215, 251) | 3.1 (2.9, 3.3) | -0.4 (-0.5, -0.4) | 26 (18, 37) | 0.3 (0.2, 0.5) | 0.2 (0, 0.5) |
| Oman | 130 (100, 172) | 4.1 (3.3, 5.2) | 0.8 (0.2, 1.6) | 244 (181, 332) | 8.2 (6.3, 10.5) | -0.5 (-0.7, -0.2) | 12 (8, 18) | 0.4 (0.3, 0.6) | 0.5 (0, 1.1) |
| Pakistan | 6008 (4794, 7436) | 3.9 (3.1, 4.9) | 0 (-0.2, 0.3) | 70855 (56391, 87639) | 45.1 (35.8, 56.4) | 0 (-0.2, 0.3) | 792 (535, 1135) | 0.6 (0.4, 0.8) | 0 (-0.2, 0.3) |
| Palau | 0 (0, 0) | 1 (0.7, 1.3) | 0.1 (-0.3, 0.6) | 2 (1, 3) | 7.4 (5.5, 9.7) | -0.1 (-0.4, 0.2) | 0 (0, 0) | 0.1 (0.1, 0.2) | 0 (-0.3, 0.5) |
| Palestine | 75 (60, 93) | 2.1 (1.7, 2.5) | 0.5 (0, 1.2) | 249 (205, 301) | 7.5 (6.2, 9.1) | -0.3 (-0.5, 0.1) | 8 (5, 11) | 0.2 (0.2, 0.3) | 0.3 (-0.1, 0.9) |
| Panama | 64 (47, 86) | 1.5 (1.1, 2) | 0.6 (0.2, 1.2) | 350 (261, 464) | 8.3 (6.2, 11) | 0 (-0.3, 0.4) | 8 (5, 11) | 0.2 (0.1, 0.3) | 0.4 (0, 0.9) |
| Papua New Guinea | 199 (136, 277) | 3.2 (2.2, 4.4) | 0 (-0.2, 0.4) | 2426 (1646, 3390) | 37.6 (25.9, 51.7) | 0 (-0.3, 0.4) | 29 (18, 44) | 0.5 (0.3, 0.7) | 0 (-0.3, 0.4) |
| Paraguay | 42 (31, 55) | 0.7 (0.5, 0.9) | 0.5 (0, 1.1) | 330 (242, 437) | 5.2 (3.8, 6.9) | 0.1 (-0.2, 0.6) | 5 (3, 8) | 0.1 (0.1, 0.1) | 0.4 (0, 0.9) |
| Peru | 174 (125, 238) | 0.5 (0.4, 0.7) | 0.1 (-0.2, 0.6) | 1135 (822, 1512) | 3.4 (2.5, 4.6) | -0.3 (-0.5, 0) | 22 (14, 32) | 0.1 (0, 0.1) | 0 (-0.3, 0.4) |
| Philippines | 4681 (3793, 5795) | 5 (4, 6.1) | -0.2 (-0.3, 0.1) | 47630 (38147, 58323) | 50.5 (40.6, 61.7) | -0.2 (-0.4, 0) | 628 (429, 876) | 0.7 (0.5, 1) | -0.2 (-0.4, 0.1) |
| Poland | 889 (714, 1088) | 1.7 (1.3, 2.1) | 0.6 (0.2, 0.9) | 5966 (4870, 7346) | 10.5 (8.5, 12.9) | -0.1 (-0.3, 0.1) | 115 (76, 159) | 0.2 (0.1, 0.3) | 0.3 (0.1, 0.6) |
| Portugal | 1519 (1092, 2032) | 9.9 (7.1, 13.3) | 2 (1.1, 3.2) | 2159 (1893, 2423) | 13.3 (11.7, 15) | -0.3 (-0.4, -0.2) | 155 (97, 234) | 0.9 (0.6, 1.5) | 1.5 (0.8, 2.5) |
| Puerto Rico | 142 (101, 191) | 2.7 (1.9, 3.7) | 0.4 (0, 0.9) | 495 (371, 656) | 8.7 (6.5, 11.6) | -0.3 (-0.5, -0.1) | 18 (11, 26) | 0.3 (0.2, 0.5) | 0.1 (-0.2, 0.6) |
| Qatar | 86 (59, 128) | 4.2 (3, 5.7) | 1.2 (0.4, 2.5) | 160 (110, 232) | 8.5 (6, 12) | -0.4 (-0.6, 0) | 8 (5, 13) | 0.4 (0.3, 0.7) | 0.8 (0.1, 1.8) |
| Republic of Korea | 7127 (5680, 8861) | 8.6 (6.9, 10.5) | 2.9 (1.9, 4.3) | 5411 (4722, 6117) | 6.5 (5.7, 7.3) | -0.5 (-0.6, -0.3) | 704 (466, 999) | 0.8 (0.6, 1.2) | 2.1 (1.2, 3.3) |
| Republic of Moldova | 122 (100, 149) | 2.6 (2.1, 3.1) | 0.5 (0.2, 0.8) | 997 (830, 1213) | 20.1 (16.8, 24.2) | 0.1 (-0.1, 0.3) | 16 (11, 22) | 0.3 (0.2, 0.4) | 0.3 (0.1, 0.7) |
| Romania | 1063 (830, 1343) | 4.3 (3.3, 5.5) | 3 (2, 4.2) | 6455 (5143, 7945) | 23.6 (18.8, 28.9) | 1.2 (0.7, 1.7) | 127 (86, 181) | 0.5 (0.3, 0.7) | 2.4 (1.6, 3.3) |
| Russian Federation | 2664 (2222, 3214) | 1.4 (1.2, 1.7) | 0.3 (0.1, 0.5) | 16361 (13617, 19622) | 8.1 (6.7, 9.7) | -0.2 (-0.3, -0.1) | 326 (219, 454) | 0.2 (0.1, 0.2) | 0.1 (0, 0.3) |
| Rwanda | 269 (181, 378) | 3.3 (2.3, 4.6) | -0.3 (-0.6, 0) | 3130 (2095, 4450) | 37.5 (25.5, 52.1) | -0.4 (-0.6, -0.1) | 36 (22, 56) | 0.5 (0.3, 0.7) | -0.4 (-0.6, 0) |
| Saint Kitts and Nevis | 2 (2, 3) | 3.4 (2.5, 4.3) | 0.2 (-0.1, 0.6) | 16 (12, 20) | 21.8 (16.6, 27.1) | -0.1 (-0.3, 0.2) | 0 (0, 0) | 0.5 (0.3, 0.6) | 0.2 (-0.1, 0.5) |
| Saint Lucia | 6 (5, 7) | 2.6 (2.2, 3.1) | 0.4 (0.1, 0.7) | 43 (36, 52) | 20 (16.6, 24.1) | 0.1 (-0.1, 0.3) | 1 (1, 1) | 0.4 (0.2, 0.5) | 0.2 (0, 0.5) |
| Saint Vincent and the Grenadines | 3 (2, 3) | 2 (1.7, 2.4) | 0.3 (0.1, 0.6) | 24 (21, 28) | 18.1 (15.6, 21.1) | 0.2 (0, 0.5) | 0 (0, 1) | 0.3 (0.2, 0.4) | 0.3 (0.1, 0.5) |
| Samoa | 9 (6, 12) | 5.2 (3.8, 7.2) | -0.1 (-0.4, 0.3) | 89 (65, 125) | 51.7 (37.7, 71.7) | -0.2 (-0.4, 0.2) | 1 (1, 2) | 0.7 (0.4, 1) | -0.2 (-0.4, 0.2) |
| San Marino | 8 (6, 12) | 17.1 (12.4, 23.4) | 0.4 (-0.1, 1) | 8 (5, 12) | 15.6 (9.6, 23.9) | -0.2 (-0.6, 0.2) | 1 (1, 1) | 1.6 (1, 2.5) | 0.3 (-0.1, 1) |
| Sao Tome and Principe | 1 (0, 1) | 0.4 (0.3, 0.6) | 0.2 (-0.1, 0.8) | 7 (5, 9) | 4.7 (3.6, 6.1) | 0.1 (-0.2, 0.7) | 0 (0, 0) | 0.1 (0, 0.1) | 0.2 (-0.1, 0.7) |
| Saudi Arabia | 4209 (3043, 5737) | 11.6 (8.6, 15.3) | 1.6 (0.8, 2.9) | 7924 (5917, 10500) | 24.4 (18.8, 31.1) | -0.4 (-0.6, -0.1) | 383 (242, 569) | 1.2 (0.7, 1.7) | 1 (0.4, 2) |
| Senegal | 79 (55, 112) | 0.8 (0.6, 1.1) | 0.3 (-0.1, 0.8) | 934 (642, 1318) | 9.4 (6.5, 13.2) | 0.2 (-0.2, 0.8) | 11 (7, 17) | 0.1 (0.1, 0.2) | 0.2 (-0.1, 0.7) |
| Serbia | 375 (273, 522) | 3.3 (2.4, 4.6) | 1.3 (0.6, 2.3) | 1375 (1052, 1782) | 11 (8.4, 14.3) | -0.1 (-0.4, 0.1) | 42 (26, 63) | 0.3 (0.2, 0.5) | 0.8 (0.3, 1.6) |
| Seychelles | 8 (7, 10) | 6.5 (5.4, 7.9) | 0.4 (0.1, 0.8) | 64 (53, 78) | 52.1 (43.7, 62.8) | 0.1 (-0.2, 0.3) | 1 (1, 1) | 0.8 (0.6, 1.2) | 0.3 (0, 0.6) |
| Sierra Leone | 41 (29, 56) | 0.8 (0.6, 1.1) | 0.3 (-0.1, 0.9) | 496 (354, 675) | 9.8 (7, 13.2) | 0.3 (-0.1, 0.9) | 6 (3, 8) | 0.1 (0.1, 0.2) | 0.3 (-0.1, 0.8) |
| Singapore | 5932 (4501, 7865) | 72.3 (55.3, 95.4) | -0.1 (-0.3, 0.3) | 4522 (3949, 5228) | 55.2 (48.4, 63.5) | -0.7 (-0.8, -0.7) | 541 (346, 787) | 6.6 (4.3, 9.6) | -0.1 (-0.4, 0.2) |
| Slovakia | 287 (204, 404) | 4 (2.8, 5.6) | 0.1 (-0.2, 0.7) | 843 (632, 1126) | 10.5 (7.9, 14) | -0.6 (-0.7, -0.4) | 30 (19, 46) | 0.4 (0.3, 0.6) | -0.1 (-0.4, 0.4) |
| Slovenia | 67 (47, 91) | 2.4 (1.7, 3.2) | 0.2 (-0.2, 0.8) | 190 (141, 251) | 5.7 (4.3, 7.6) | -0.6 (-0.7, -0.4) | 7 (5, 11) | 0.2 (0.1, 0.4) | 0 (-0.4, 0.5) |
| Solomon Islands | 24 (13, 38) | 5.7 (3.2, 8.7) | 0 (-0.3, 0.4) | 283 (145, 448) | 64.5 (35.4, 99.5) | 0 (-0.3, 0.4) | 3 (2, 6) | 0.8 (0.4, 1.4) | 0 (-0.3, 0.4) |
| Somalia | 354 (238, 503) | 3.6 (2.4, 5.1) | -0.1 (-0.4, 0.3) | 4603 (3109, 6571) | 44.4 (30.4, 63.6) | -0.1 (-0.4, 0.2) | 48 (29, 75) | 0.5 (0.3, 0.8) | -0.2 (-0.4, 0.2) |
| South Africa | 591 (530, 661) | 1.2 (1.1, 1.3) | -0.2 (-0.3, -0.1) | 5899 (5282, 6632) | 11.8 (10.6, 13.3) | -0.3 (-0.3, -0.2) | 85 (60, 114) | 0.2 (0.1, 0.2) | -0.2 (-0.3, -0.1) |
| South Sudan | 129 (79, 202) | 2.5 (1.5, 3.9) | -0.2 (-0.5, 0.2) | 1593 (939, 2558) | 29.2 (17.3, 46.6) | -0.2 (-0.5, 0.2) | 18 (10, 30) | 0.4 (0.2, 0.6) | -0.2 (-0.5, 0.2) |
| Spain | 7358 (5346, 9977) | 10.2 (7.4, 13.8) | 0.4 (0, 0.9) | 7058 (6241, 7895) | 9.7 (8.5, 10.9) | -0.5 (-0.6, -0.4) | 748 (463, 1107) | 1 (0.6, 1.5) | 0.3 (-0.1, 0.8) |
| Sri Lanka | 894 (638, 1214) | 3.6 (2.5, 4.9) | 0.3 (-0.1, 0.8) | 5521 (4007, 7490) | 21.2 (15.5, 28.7) | -0.2 (-0.5, 0.1) | 111 (67, 170) | 0.4 (0.3, 0.7) | 0.1 (-0.2, 0.5) |
| Sudan | 416 (252, 617) | 1.5 (0.9, 2.1) | 0 (-0.3, 0.6) | 2523 (1721, 3548) | 9.7 (6.8, 13.5) | -0.4 (-0.6, 0) | 46 (27, 72) | 0.2 (0.1, 0.3) | -0.1 (-0.4, 0.3) |
| Suriname | 17 (13, 20) | 2.7 (2.1, 3.3) | 0.4 (0.1, 0.8) | 159 (127, 196) | 25.3 (20.3, 30.9) | 0.3 (0, 0.6) | 2 (2, 3) | 0.4 (0.3, 0.5) | 0.4 (0.1, 0.7) |
| Sweden | 422 (346, 511) | 3 (2.4, 3.6) | 0.1 (-0.2, 0.3) | 445 (406, 486) | 3 (2.8, 3.3) | -0.4 (-0.4, -0.3) | 45 (31, 62) | 0.3 (0.2, 0.4) | 0 (-0.2, 0.3) |
| Switzerland | 1027 (747, 1378) | 7.5 (5.4, 10) | 0.6 (0.2, 1.3) | 911 (787, 1053) | 6.6 (5.7, 7.5) | -0.1 (-0.2, 0) | 104 (64, 153) | 0.7 (0.4, 1.1) | 0.6 (0.1, 1.2) |
| Syrian Arab Republic | 205 (150, 285) | 1.4 (1, 2) | 1.5 (0.7, 2.9) | 524 (392, 710) | 3.6 (2.7, 4.8) | -0.2 (-0.5, 0.2) | 20 (12, 30) | 0.1 (0.1, 0.2) | 1 (0.4, 2) |
| Taiwan (Province of China) | 13522 (9836, 18551) | 42.2 (30.9, 58.2) | 0.4 (0, 1) | 27902 (20907, 37180) | 77.6 (58.3, 102.8) | -0.5 (-0.6, -0.3) | 1281 (790, 1934) | 3.9 (2.4, 5.9) | 0.2 (-0.1, 0.8) |
| Tajikistan | 92 (71, 116) | 1.5 (1.2, 1.8) | 0 (-0.3, 0.3) | 1009 (784, 1269) | 15.6 (12.2, 19.3) | -0.1 (-0.3, 0.2) | 13 (8, 18) | 0.2 (0.1, 0.3) | 0 (-0.3, 0.3) |
| Thailand | 5903 (4130, 8003) | 6.4 (4.5, 8.7) | 0.4 (0, 1) | 34508 (24804, 46177) | 34.9 (25.2, 46.4) | -0.3 (-0.5, 0) | 695 (421, 1036) | 0.7 (0.4, 1.1) | 0.2 (-0.2, 0.6) |
| The Republic of Côte d'Ivoire | 144 (100, 198) | 0.9 (0.6, 1.2) | 0.2 (-0.2, 0.7) | 1758 (1218, 2411) | 10.6 (7.4, 14.3) | 0.2 (-0.2, 0.7) | 19 (12, 29) | 0.1 (0.1, 0.2) | 0.2 (-0.2, 0.7) |
| Timor-Leste | 36 (23, 49) | 3.9 (2.6, 5.4) | -0.1 (-0.4, 0.4) | 387 (241, 526) | 42.1 (27.4, 57.7) | -0.1 (-0.4, 0.3) | 5 (3, 8) | 0.6 (0.3, 0.9) | -0.1 (-0.4, 0.4) |
| Togo | 47 (33, 63) | 0.9 (0.6, 1.2) | 0.3 (-0.1, 0.9) | 565 (394, 763) | 10.5 (7.5, 14.1) | 0.3 (-0.1, 0.9) | 6 (4, 10) | 0.1 (0.1, 0.2) | 0.3 (-0.1, 0.8) |
| Tokelau | 0 (0, 0) | 3 (2.1, 4.1) | 0 (-0.3, 0.4) | 0 (0, 1) | 27.8 (20.1, 38) | -0.2 (-0.4, 0.1) | 0 (0, 0) | 0.4 (0.3, 0.6) | -0.1 (-0.4, 0.3) |
| Tonga | 2 (2, 3) | 2.7 (2, 3.7) | 0.1 (-0.2, 0.5) | 23 (17, 32) | 27.4 (20, 37.9) | 0 (-0.3, 0.4) | 0 (0, 0) | 0.4 (0.2, 0.6) | 0 (-0.2, 0.5) |
| Trinidad and Tobago | 22 (16, 30) | 1.3 (0.9, 1.8) | 0.4 (0, 1) | 177 (130, 236) | 10.4 (7.6, 13.9) | 0.1 (-0.2, 0.5) | 3 (2, 4) | 0.2 (0.1, 0.3) | 0.3 (-0.1, 0.7) |
| Tunisia | 3772 (2594, 5263) | 28.4 (19.6, 39.4) | 1.4 (0.5, 2.6) | 7031 (5022, 9702) | 52.8 (37.7, 72.7) | -0.3 (-0.5, 0.1) | 361 (220, 551) | 2.7 (1.7, 4.2) | 1 (0.3, 1.9) |
| Turkey | 5994 (4608, 7689) | 6.5 (5, 8.3) | 1 (0.4, 1.8) | 12906 (10106, 16062) | 14 (11, 17.4) | -0.5 (-0.6, -0.3) | 594 (385, 846) | 0.6 (0.4, 0.9) | 0.5 (0.1, 1.1) |
| Turkmenistan | 74 (58, 95) | 1.5 (1.2, 1.9) | 0.7 (0.3, 1.2) | 696 (565, 862) | 14.4 (11.7, 17.7) | 0.4 (0.1, 0.7) | 9 (6, 12) | 0.2 (0.1, 0.3) | 0.6 (0.2, 1) |
| Tuvalu | 0 (0, 1) | 4 (2.7, 6.4) | -0.1 (-0.4, 0.2) | 5 (3, 8) | 44.1 (29.8, 69.1) | -0.2 (-0.4, 0.1) | 0 (0, 0) | 0.6 (0.3, 1) | -0.2 (-0.4, 0.2) |
| Uganda | 1182 (884, 1560) | 5.7 (4.3, 7.5) | -0.1 (-0.3, 0.3) | 14069 (10449, 18777) | 65.9 (49.3, 85.8) | -0.1 (-0.4, 0.2) | 156 (101, 228) | 0.8 (0.5, 1.2) | -0.1 (-0.4, 0.2) |
| Ukraine | 3317 (2675, 4060) | 6 (4.9, 7.3) | 1.5 (0.9, 2.4) | 10571 (8502, 13110) | 17.6 (14.2, 21.6) | 0.7 (0.3, 1.3) | 347 (230, 485) | 0.6 (0.4, 0.8) | 1.3 (0.7, 2.1) |
| United Arab Emirates | 270 (171, 403) | 2.5 (1.7, 3.4) | 0.1 (-0.2, 0.6) | 1407 (889, 2302) | 13 (8.9, 19.5) | -0.3 (-0.5, 0) | 28 (16, 45) | 0.3 (0.2, 0.4) | 0 (-0.3, 0.4) |
| United Kingdom | 6896 (5688, 8276) | 7.3 (6, 8.7) | 0.1 (-0.1, 0.3) | 6960 (6660, 7254) | 7.3 (7, 7.6) | -0.3 (-0.3, -0.2) | 707 (488, 977) | 0.7 (0.5, 1) | 0 (-0.1, 0.2) |
| United Republic of Tanzania | 1110 (732, 1610) | 3.4 (2.3, 4.9) | -0.1 (-0.4, 0.2) | 13007 (8547, 19088) | 38.6 (25.7, 55.6) | -0.2 (-0.4, 0.1) | 150 (89, 236) | 0.5 (0.3, 0.8) | -0.2 (-0.4, 0.1) |
| United States of America | 15817 (13379, 18626) | 3.6 (3.1, 4.3) | 0 (-0.2, 0.2) | 30684 (29349, 32087) | 6.6 (6.4, 6.9) | -0.4 (-0.4, -0.3) | 1681 (1126, 2279) | 0.4 (0.2, 0.5) | -0.1 (-0.2, 0.1) |
| United States Virgin Islands | 5 (4, 6) | 2.9 (2.3, 3.7) | 0.3 (0, 0.7) | 32 (26, 39) | 19.7 (15.8, 24.1) | 0 (-0.2, 0.3) | 1 (0, 1) | 0.4 (0.3, 0.6) | 0.2 (-0.1, 0.6) |
| Uruguay | 103 (75, 136) | 2.4 (1.8, 3.2) | 0.1 (-0.2, 0.6) | 444 (393, 502) | 10.2 (9.1, 11.5) | -0.3 (-0.4, -0.2) | 13 (8, 19) | 0.3 (0.2, 0.4) | 0 (-0.3, 0.4) |
| Uzbekistan | 587 (474, 715) | 1.9 (1.6, 2.3) | 0.8 (0.3, 1.4) | 5510 (4509, 6618) | 18.2 (14.9, 21.8) | 0.5 (0.1, 1) | 71 (49, 99) | 0.3 (0.2, 0.4) | 0.8 (0.3, 1.4) |
| Vanuatu | 8 (5, 11) | 3.9 (2.7, 5.6) | 0.1 (-0.3, 0.6) | 94 (63, 134) | 45.5 (31, 64.7) | 0.1 (-0.3, 0.6) | 1 (1, 2) | 0.6 (0.4, 0.9) | 0.1 (-0.3, 0.6) |
| Venezuela (Bolivarian Republic of) | 380 (276, 513) | 1.3 (0.9, 1.7) | 1 (0.4, 1.7) | 2522 (1850, 3300) | 8.4 (6.2, 11) | 0.3 (0, 0.8) | 48 (31, 71) | 0.2 (0.1, 0.2) | 0.7 (0.3, 1.3) |
| Viet Nam | 12330 (9236, 16364) | 11.3 (8.6, 14.9) | 0.2 (-0.2, 0.7) | 91964 (69559, 120065) | 85 (64.7, 109.2) | -0.2 (-0.4, 0.1) | 1486 (968, 2120) | 1.4 (0.9, 2) | 0 (-0.3, 0.5) |
| Yemen | 287 (187, 421) | 1.5 (1, 2.1) | 0 (-0.3, 0.5) | 2143 (1485, 3017) | 11.5 (8.3, 16.2) | -0.3 (-0.5, 0.1) | 34 (20, 53) | 0.2 (0.1, 0.3) | -0.1 (-0.4, 0.3) |
| Zambia | 376 (265, 510) | 3.8 (2.7, 5.1) | -0.1 (-0.4, 0.2) | 4503 (3165, 6118) | 43.9 (31.4, 59.5) | -0.2 (-0.4, 0.1) | 50 (31, 76) | 0.5 (0.3, 0.8) | -0.2 (-0.4, 0.1) |
| Zimbabwe | 182 (136, 239) | 1.9 (1.4, 2.5) | 0.2 (-0.1, 0.6) | 2166 (1621, 2880) | 22.3 (16.7, 29.2) | 0.2 (-0.1, 0.6) | 25 (16, 36) | 0.3 (0.2, 0.4) | 0.2 (-0.1, 0.5) |

UI, uncertainty interval; YLDs, years lived with disability; YLLs, years of life lost.

Supplementary Table 9 The new counts and incidence rates for NPC in 2035 for 21 GBD regions by Global Burden of Disease.

|  | Counts | ASR per 100,000 population (95%UI) |
| --- | --- | --- |
| Global | 272980 (187195 to 358765) | 2.4（2.2 to 2.7) |
| Andean Latin America | 143 (122 to 165) | 0.2 (0.2 to 0.2) |
| Australasia | 356 (313 to 399) | 0.6 (0.5 to 0.8) |
| Caribbean | 439 (418 to 461) | 0.7 (0.7 to 0.8) |
| Central Asia | 504 (422 to 586) | 0.5 (0.3 to 0.6) |
| Central Europe | 1146 (539 to 1754) | 0.7 (0.3 to 1.1) |
| Central Latin America | 758 (702 to 814) | 0.2 (0.2 to 0.3) |
| Central Sub-Saharan Africa | 509 (427 to 590) | 0.5 (0.4 to 0.5) |
| East Asia | 185828 (95853 to 275804) | 7.9 (2.5 to 13.3) |
| Eastern Europe | 1513 (968 to 2059) | 0.5 (0.3 to 0.7) |
| Eastern Sub-Saharan Africa | 3872 (3153 to 4592) | 1.1 (1 to 1.2) |
| High-income Asia Pacific | 6959 (5145 to 8774) | 1.7 (0.8 to 2.5) |
| High-income North America | 4132 (3705 to 4560) | 0.7 (0.5 to 0.8) |
| North Africa and Middle East | 11327 (9704 to 12950) | 1.5 (1.2 to 1.9) |
| Oceania | 162 (126 to 198) | 1.3 (1.2 to 1.4) |
| South Asia | 22137 (15875 to 28399) | 0.8 (0.6 to 1.1) |
| Southeast Asia | 19932 (16022 to 23842) | 2.1 (1.7 to 2.6) |
| Southern Latin America | 320 (230 to 410) | 0.3 (0.2 to 0.5) |
| Southern Sub-Saharan Africa | 332 (162 to 501) | 0.3 (-0.2 to 0.8) |
| Tropical Latin America | 873 (638 to 1109) | 0.3 (0.1 to 0.4) |
| Western Europe | 9368 (7225 to 11512) | 1.2 (1.1 to 1.3) |
| Western Sub-Saharan Africa | 1872 (1341 to 2404) | 0.6 (0.5 to 0.6) |

UI=uncertainty interval.

Supplementary Table 10 The new counts and incidence rates for NPC in 2035 for 204 countries.

|  | Counts | ASR per 100,000 population (95%UI) |
| --- | --- | --- |
| Afghanistan | 170 (111 to 229) | 0.5 (0.4 to 0.6) |
| Albania | 21 (-4 to 47) | 0.4 (0.3 to 0.6) |
| Algeria | 3108 (2293 to 3923) | 3.7 (3.3 to 4) |
| American Samoa | 1 (1 to 1) | 1.7 (1.2 to 2.1) |
| Andorra | 3 (2 to 3) | 1.3 (0.6 to 2.1) |
| Angola | 125 (98 to 153) | 0.5 (0.5 to 0.6) |
| Antigua and Barbuda | 1 (1 to 1) | 0.5 (0.4 to 0.6) |
| Argentina | 127 (111 to 143) | 0.3 (0.2 to 0.5) |
| Armenia | 10 (8 to 13) | 0.3 (0.2 to 0.3) |
| Australia | 304 (264 to 344) | 0.6 (0.5 to 0.8) |
| Austria | 86 (65 to 107) | 0.6 (0.4 to 0.8) |
| Azerbaijan | 22 (9 to 35) | 0.2 (0.1 to 0.3) |
| Bahamas | 3 (3 to 4) | 0.7 (0.6 to 0.7) |
| Bahrain | 18 (12 to 24) | 0.6 (0.3 to 0.9) |
| Bangladesh | 1932 (1246 to 2619) | 0.9 (0.2 to 1.5) |
| Barbados | 5 (4 to 5) | 0.9 (0.8 to 1) |
| Belarus | 81 (63 to 98) | 0.5 (0.4 to 0.6) |
| Belgium | 218 (99 to 337) | 1.2 (0.6 to 1.9) |
| Belize | 2 (1 to 3) | 0.4 (0.2 to 0.6) |
| Benin | 29 (23 to 34) | 0.3 (0.3 to 0.4) |
| Bermuda | 1 (1 to 1) | 1.1 (1 to 1.2) |
| Bhutan | 7 (6 to 9) | 0.9 (0.6 to 1.1) |
| Bolivia (Plurinational State of) | 32 (29 to 35) | 0.2 (0.2 to 0.3) |
| Bosnia and Herzegovina | 13 (3 to 22) | 0.2 (0.1 to 0.4) |
| Botswana | 10 (8 to 11) | 0.4 (-0.1 to 0.8) |
| Brazil | 855 (620 to 1090) | 0.3 (0.1 to 0.4) |
| Brunei Darussalam | 35 (20 to 51) | 5.4 (3.9 to 6.8) |
| Bulgaria | 91 (69 to 114) | 0.9 (0.7 to 1.2) |
| Burkina Faso | 54 (32 to 76) | 0.4 (0.3 to 0.4) |
| Burundi | 133 (86 to 181) | 1.4 (0.9 to 1.9) |
| Cabo Verde | 3 (2 to 3) | 0.3 (0.1 to 0.6) |
| Cambodia | 392 (337 to 448) | 2 (1.5 to 2.6) |
| Cameroon | 221 (177 to 265) | 0.8 (0.2 to 1.4) |
| Canada | 825 (757 to 893) | 1.2 (1.1 to 1.4) |
| Central African Republic | 24 (21 to 27) | 0.6 (0.5 to 0.6) |
| Chad | 36 (30 to 42) | 0.3 (0.1 to 0.5) |
| Chile | 82 (54 to 109) | 0.3 (0.1 to 0.5) |
| China | 181819 (92691 to 270947) | 8 (2.5 to 13.5) |
| Colombia | 148 (124 to 172) | 0.2 (0.1 to 0.3) |
| Comoros | 8 (6 to 10) | 1.1 (0.8 to 1.5) |
| Congo | 27 (16 to 39) | 0.4 (0.3 to 0.6) |
| Cook Islands | 0 (0 to 0) | 0.3 (-0.1 to 0.7) |
| Costa Rica | 51 (46 to 55) | 0.8 (0.7 to 1) |
| Croatia | 37 (26 to 48) | 0.6 (0.4 to 0.8) |
| Cuba | 180 (164 to 196) | 0.9 (0.8 to 1) |
| Cyprus | 17 (3 to 30) | 1 (0.8 to 1.2) |
| Czechia | 63 (53 to 73) | 0.3 (0.3 to 0.4) |
| Democratic People's Republic of Korea | 687 (635 to 738) | 1.6 (1.4 to 1.7) |
| Democratic Republic of the Congo | 312 (261 to 363) | 0.4 (0.4 to 0.5) |
| Denmark | 70 (62 to 79) | 0.8 (0.6 to 0.9) |
| Djibouti | 16 (13 to 19) | 1.3 (1.1 to 1.5) |
| Dominica | 1 (1 to 1) | 0.6 (0.3 to 1) |
| Dominican Republic | 59 (48 to 70) | 0.2 (-0.4 to 0.8) |
| Ecuador | 40 (35 to 45) | 0.2 (0.2 to 0.2) |
| Egypt | 206 (176 to 236) | 0.2 (0.2 to 0.2) |
| El Salvador | 18 (14 to 21) | 0.2 (0.2 to 0.3) |
| Equatorial Guinea | 4 (2 to 7) | 0.4 (-0.3 to 1.1) |
| Eritrea | 70 (65 to 74) | 1.3 (0.8 to 1.7) |
| Estonia | 9 (3 to 15) | 0.5 (0.2 to 0.8) |
| Eswatini | 6 (2 to 10) | 0.8 (0.7 to 1) |
| Ethiopia | 771 (428 to 1113) | 0.9 (0.4 to 1.5) |
| Fiji | 4 (3 to 5) | 0.4 (0.3 to 0.5) |
| Finland | 52 (46 to 57) | 0.6 (0.5 to 0.6) |
| France | 1728 (1497 to 1959) | 1.6 (1.1 to 2) |
| Gabon | 9 (7 to 10) | 0.5 (0.4 to 0.5) |
| Gambia | 4 (3 to 4) | 0.2 (0.2 to 0.3) |
| Georgia | 17 (6 to 28) | 0.3 (0.1 to 0.5) |
| Germany | 1408 (1212 to 1605) | 0.9 (0.4 to 1.4) |
| Ghana | 28 (19 to 36) | 0.2 (0 to 0.3) |
| Greece | 309 (153 to 465) | 1.7 (0.7 to 2.8) |
| Greenland | 4 (3 to 5) | 3.6 (0.7 to 6.5) |
| Grenada | 1 (1 to 1) | 0.6 (0.5 to 0.8) |
| Guam | 7 (6 to 8) | 2.7 (2.4 to 3) |
| Guatemala | 36 (9 to 63) | 0.3 (0.1 to 0.4) |
| Guinea | 25 (24 to 27) | 0.2 (0.1 to 0.3) |
| Guinea-Bissau | 6 (5 to 7) | 0.4 (0.2 to 0.6) |
| Guyana | 3 (3 to 3) | 0.3 (0.3 to 0.4) |
| Haiti | 87 (67 to 107) | 0.8 (0.7 to 0.8) |
| Honduras | 15 (11 to 19) | 0.2 (0.1 to 0.2) |
| Hungary | 136 (-18 to 290) | 1 (-0.1 to 2.1) |
| Iceland | 5 (4 to 5) | 0.8 (0.7 to 0.9) |
| India | 17146 (11333 to 22958) | 0.8 (0.5 to 1.1) |
| Indonesia | 4249 (4123 to 4375) | 1.3 (1 to 1.6) |
| Iran (Islamic Republic of) | 462 (380 to 545) | 0.4 (0.3 to 0.5) |
| Iraq | 198 (137 to 258) | 0.5 (0.2 to 0.8) |
| Ireland | 78 (66 to 89) | 1 (0.8 to 1.2) |
| Israel | 88 (72 to 104) | 0.8 (-0.1 to 1.6) |
| Italy | 1530 (1015 to 2046) | 1.4 (0.6 to 2.2) |
| Jamaica | 27 (21 to 33) | 0.8 (0.6 to 1.1) |
| Japan | 4242 (2537 to 5946) | 1.3 (0.4 to 2.2) |
| Jordan | 175 (86 to 264) | 1 (0.7 to 1.3) |
| Kazakhstan | 104 (68 to 139) | 0.5 (0.4 to 0.5) |
| Kenya | 785 (592 to 978) | 1.6 (1 to 2.3) |
| Kiribati | 2 (1 to 2) | 1.4 (0.9 to 1.9) |
| Kuwait | 41 (11 to 72) | 0.7 (-0.1 to 1.4) |
| Kyrgyzstan | 28 (22 to 34) | 0.4 (0.3 to 0.5) |
| Lao People's Democratic Republic | 111 (90 to 133) | 1.5 (0.9 to 2.1) |
| Latvia | 11 (-2 to 23) | 0.4 (0 to 0.7) |
| Lebanon | 106 (65 to 147) | 1.6 (1.5 to 1.8) |
| Lesotho | 16 (13 to 19) | 0.9 (0.2 to 1.5) |
| Liberia | 13 (9 to 18) | 0.3 (0.3 to 0.3) |
| Libya | 560 (118 to 1001) | 4.8 (3.2 to 6.4) |
| Lithuania | 9 (4 to 15) | 0.2 (0.1 to 0.4) |
| Luxembourg | 18 (13 to 23) | 1.6 (1.2 to 2) |
| Madagascar | 228 (185 to 272) | 0.9 (0.8 to 1) |
| Malawi | 45 (25 to 64) | 0.3 (0.2 to 0.4) |
| Malaysia | 3801 (2446 to 5156) | 10 (4.5 to 15.4) |
| Maldives | 2 (2 to 3) | 0.3 (0 to 0.7) |
| Mali | 23 (14 to 33) | 0.1 (0 to 0.2) |
| Malta | 26 (23 to 30) | 3.5 (2.9 to 4.1) |
| Marshall Islands | 1 (1 to 1) | 1.8 (1.7 to 2) |
| Mauritania | 7 (5 to 9) | 0.2 (0.2 to 0.3) |
| Mauritius | 18 (15 to 21) | 0.8 (0.7 to 1) |
| Mexico | 328 (265 to 392) | 0.2 (0.2 to 0.2) |
| Micronesia (Federated States of) | 2 (1 to 2) | 1.6 (1.3 to 1.9) |
| Monaco | 1 (0 to 1) | 1 (0.6 to 1.3) |
| Mongolia | 14 (10 to 18) | 0.3 (0.1 to 0.5) |
| Montenegro | 2 (2 to 2) | 0.2 (0.2 to 0.2) |
| Morocco | 1535 (1275 to 1795) | 3.3 (2.5 to 4) |
| Mozambique | 30 (22 to 37) | 0.1 (0.1 to 0.2) |
| Myanmar | 807 (620 to 995) | 1.3 (0.9 to 1.7) |
| Namibia | 12 (9 to 14) | 0.6 (0.5 to 0.7) |
| Nauru | 0 (0 to 0) | 1.5 (0.4 to 2.6) |
| Nepal | 359 (285 to 432) | 1.1 (0.6 to 1.5) |
| Netherlands | 428 (382 to 473) | 1.4 (1.2 to 1.6) |
| New Zealand | 52 (40 to 63) | 0.7 (0.4 to 0.9) |
| Nicaragua | 24 (17 to 30) | 0.4 (0.3 to 0.4) |
| Niger | 16 (11 to 22) | 0.1 (0.1 to 0.1) |
| Nigeria | 1244 (813 to 1676) | 0.9 (0.9 to 0.9) |
| Niue | 0 (0 to 0) | 1.1 (1.1 to 1.2) |
| North Macedonia | 12 (10 to 14) | 0.4 (0.3 to 0.4) |
| Northern Mariana Islands | 3 (2 to 3) | 3.1 (2.6 to 3.7) |
| Norway | 49 (43 to 54) | 0.5 (0.2 to 0.7) |
| Oman | 30 (25 to 35) | 0.8 (0.7 to 0.9) |
| Pakistan | 2615 (2428 to 2803) | 1.2 (0.3 to 2) |
| Palau | 0 (0 to 0) | 0.3 (0.3 to 0.3) |
| Palestine | 26 (19 to 33) | 0.6 (0.4 to 0.8) |
| Panama | 25 (18 to 32) | 0.4 (0.3 to 0.5) |
| Papua New Guinea | 117 (97 to 136) | 1.3 (1.2 to 1.4) |
| Paraguay | 16 (14 to 18) | 0.2 (0.1 to 0.2) |
| Peru | 64 (56 to 72) | 0.1 (0.1 to 0.2) |
| Philippines | 2180 (1632 to 2728) | 1.7 (1.3 to 2.2) |
| Poland | 265 (-38 to 567) | 0.4 (-0.2 to 1) |
| Portugal | 340 (313 to 368) | 1.8 (1.3 to 2.4) |
| Puerto Rico | 40 (36 to 45) | 0.6 (0.5 to 0.6) |
| Qatar | 28 (18 to 39) | 0.8 (0.6 to 1) |
| Republic of Korea | 1581 (1391 to 1772) | 1.7 (1.4 to 2) |
| Republic of Moldova | 28 (-11 to 67) | 0.7 (0.5 to 1) |
| Romania | 324 (6 to 642) | 1.2 (0.1 to 2.4) |
| Russian Federation | 703 (600 to 806) | 0.4 (0.2 to 0.5) |
| Rwanda | 93 (43 to 143) | 1.1 (0 to 2.2) |
| Saint Kitts and Nevis | 2 (1 to 3) | 1 (0.8 to 1.2) |
| Saint Lucia | 3 (2 to 3) | 0.9 (0.8 to 1) |
| Saint Vincent and the Grenadines | 1 (1 to 1) | 0.8 (0.7 to 0.9) |
| Samoa | 4 (3 to 4) | 1.7 (1.3 to 2) |
| San Marino | 2 (2 to 2) | 2.9 (2.7 to 3.1) |
| Sao Tome and Principe | 0 (0 to 0) | 0.2 (0.1 to 0.2) |
| Saudi Arabia | 1372 (981 to 1764) | 2.5 (2.1 to 2.8) |
| Senegal | 37 (31 to 43) | 0.3 (0.2 to 0.4) |
| Serbia | 98 (86 to 111) | 0.8 (0.7 to 0.9) |
| Seychelles | 3 (3 to 4) | 2 (1.6 to 2.4) |
| Sierra Leone | 23 (17 to 28) | 0.3 (0.2 to 0.4) |
| Singapore | 1156 (866 to 1447) | 10.8 (7.2 to 14.4) |
| Slovakia | 57 (49 to 66) | 0.8 (0.4 to 1.2) |
| Slovenia | 13 (12 to 14) | 0.4 (0.3 to 0.5) |
| Solomon Islands | 12 (10 to 13) | 2.1 (1.9 to 2.3) |
| Somalia | 181 (136 to 227) | 1.2 (1.1 to 1.3) |
| South Africa | 203 (35 to 371) | 0.2 (-0.4 to 0.8) |
| South Sudan | 58 (42 to 73) | 0.8 (0.7 to 0.9) |
| Spain | 1452 (1306 to 1598) | 1.5 (1.4 to 1.7) |
| Sri Lanka | 308 (250 to 367) | 1 (0.9 to 1.1) |
| Sudan | 172 (139 to 205) | 0.5 (0.4 to 0.6) |
| Suriname | 8 (7 to 9) | 0.9 (0.6 to 1.2) |
| Sweden | 69 (55 to 83) | 0.5 (0.4 to 0.6) |
| Switzerland | 188 (57 to 319) | 1.3 (0.9 to 1.6) |
| Syrian Arab Republic | 51 (38 to 64) | 0.3 (0.3 to 0.4) |
| Taiwan (Province of China) | 2972 (2350 to 3595) | 6.9 (6 to 7.7) |
| Tajikistan | 46 (25 to 67) | 0.6 (0.5 to 0.6) |
| Thailand | 2016 (1814 to 2218) | 1.6 (1.4 to 1.7) |
| The Republic of Côte d'Ivoire | 66 (61 to 71) | 0.2 (0 to 0.5) |
| Timor-Leste | 20 (13 to 28) | 1.9 (0.8 to 3) |
| Togo | 24 (16 to 33) | 0.4 (0.3 to 0.4) |
| Tokelau | 0 (0 to 0) | 0.9 (0.8 to 1) |
| Tonga | 1 (1 to 1) | 1 (0.8 to 1.1) |
| Trinidad and Tobago | 9 (8 to 10) | 0.4 (0.3 to 0.5) |
| Tunisia | 957 (717 to 1198) | 5.9 (5.5 to 6.3) |
| Turkey | 1423 (1307 to 1538) | 1.2 (1 to 1.4) |
| Turkmenistan | 27 (23 to 32) | 0.5 (0.4 to 0.7) |
| Tuvalu | 0 (0 to 0) | 1.5 (1.1 to 1.9) |
| Uganda | 539 (442 to 637) | 2.1 (1 to 3.1) |
| Ukraine | 849 (596 to 1101) | 1.5 (1 to 1.9) |
| United Arab Emirates | 119 (76 to 161) | 0.6 (0.4 to 0.8) |
| United Kingdom | 1263 (1143 to 1383) | 1.1 (1 to 1.3) |
| United Republic of Tanzania | 572 (401 to 743) | 1.1 (1 to 1.2) |
| United States of America | 3318 (2901 to 3736) | 0.6 (0.5 to 0.8) |
| United States Virgin Islands | 2 (1 to 2) | 0.9 (0.7 to 1) |
| Uruguay | 25 (18 to 31) | 0.5 (0.4 to 0.7) |
| Uzbekistan | 235 (209 to 261) | 0.7 (0.6 to 0.9) |
| Vanuatu | 4 (3 to 6) | 1.5 (1.4 to 1.6) |
| Venezuela (Bolivarian Republic of) | 153 (138 to 169) | 0.4 (0.4 to 0.5) |
| Viet Nam | 5254 (4369 to 6140) | 3.1 (2.9 to 3.4) |
| Yemen | 126 (102 to 150) | 0.5 (0.4 to 0.6) |
| Zambia | 199 (146 to 252) | 1.2 (0.4 to 2.1) |
| Zimbabwe | 82 (71 to 93) | 0.7 (0.5 to 1) |

Supplementary Table 11 SDI quintile in 2019.

| SDI_quintile | lower_bound | upper_bound |
| --- | --- | --- |
| Low SDI | 0 | 0.454743 |
| Low-middle SDI | 0.454743 | 0.607679 |
| Middle SDI | 0.607679 | 0.689504 |
| High-middle SDI | 0.689504 | 0.805129 |
| High SDI | 0.805129 | 1 |

SDI=Socio-demographic Index.

| Sequela | Health state name | Health state lay description | Disability Weight |
| --- | --- | --- | --- |
| Diagnosis and primary therapy phase of nasopharynx cancer | Cancer, diagnosis and primary therapy | has pain, nausea, fatigue, weight loss and high anxiety. | 0.288 (0.193-0.399) |
| Metastatic phase of nasopharynx cancer | Cancer, metastatic | has severe pain, extreme fatigue, weight loss and high anxiety. | 0.451 (0.307-0.6) |
| Terminal phase of nasopharynx cancer | Terminal phase, with medication (for cancers, end-stage kidney/liver disease) | has lost a lot of weight and regularly uses strong medication to avoid constant pain. The person has no appetite, feels nauseous, and needs to spend most of the day in bed. | 0.54 (0.377-0.687) |
| Controlled phase of nasopharynx cancer | Generic uncomplicated disease: worry and daily medication | has a chronic disease that requires medication every day and causes some worry but minimal interference with daily activities. | 0.049 (0.031-0.072) |

Supplementary Table 12 GBD 2019 sequelae, health states, health state lay descriptions, and disability weights for Nasopharyngeal Cancer.
